# A Blue Emissive Heavy Atom‐Free Multiresonant Thermally Activated Delayed Fluorescent Emitter Shows Ultra‐Fast Reverse Intersystem Crossing

**DOI:** 10.1002/anie.9957319

**Published:** 2026-07-13

**Authors:** Wenrui Wang, Hassan Hafeez, Sen Wu, Aidan P. McKay, David B. Cordes, Ifor D. W. Samuel, Eli Zysman‐Colman

**Affiliations:** ^1^ Organic Semiconductor Centre, EaStCHEM School of Chemistry University of St Andrews St Andrews UK; ^2^ Organic Semiconductor Centre, SUPA School of Physics and Astronomy University of St Andrews St Andrews UK

**Keywords:** carbonyl‐boron hybrid, efficiency roll‐off, heavy atom‐free, MR‐TADF, OLED

## Abstract

One of the key factors affecting the stability and efficiency roll‐off of organic light‐emitting diodes is the rate constant of reverse intersystem crossing (*k*_RISC_), which is itself influenced by both the magnitude of the singlet‐triplet energy gap (Δ*E*_ST_) and spin‐orbit coupling (SOC) matrix elements. Most MR‐TADF emitters possess relatively large Δ*E*_ST_ and small SOC. With the goal of designing an MR‐TADF compound having a small Δ*E*_ST_ and strong SOC that emits in the deep blue region, here we report a heavy atom‐free boron‐nitrogen‐carbonyl hybrid MR‐TADF emitter, **DDOBDiKTa**, which emits at *λ*_PL_ of 438 nm in toluene with a narrow full width at half maximum (FWHM) of 33 nm. **DDOBDiKTa** has a *Φ*_PL_ of 70% and a Δ*E*_ST_ of 0.05 eV in 5 wt% doped films in 26DCzPPy. Remarkably, without any heavy atoms, this emitter shows fast prompt, *τ*_p_, and delayed, *τ*_d_, fluorescence lifetimes of 2.3 ns and 1.48 µs, respectively, leading to a *k*_RISC_ of 1.98 ×10^6^ s^−1^ in 26DCzPPy. This is one of the fastest *k*_RISC_ values among reported MR‐TADF emitters that have no heavy atoms. The OLED showed EQE_max_/EQE_10000_ of 23.0%/10%, demonstrating unusually mild efficiency roll‐off for a blue device.

## Graphical abstract

The extended π‐system and low‐lying n‐π∗ transition endow **DDOBDiKTa** with a synergistic combination of strong spin‐orbit coupling, small reorganization energy and small Δ*E*_ST_, enabling this blue boron‐nitrogen‐carbonyl hybrid emitter to achieve a fast *k*_RISC_ of 6.34 × 10^6^ s^−1^ together with OLEDs showing small efficiency roll‐off, with EQE_max_/EQE_10000_ of 23.0%/10%, respectively.

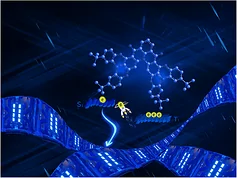

## 1 Introduction

The low maximum external quantum efficiency (EQE_max_) of fluorescent organic light‐emitting diodes (OLEDs) and the absence of stable blue phosphorescent OLEDs have driven an intense effort to identify new generations of materials and devices that address these deficiencies. TADF emitters have attracted significant attention due to their ability to harvest both singlet and triplet excitons to produce light in electroluminescent devices [1, 2].

Conventional donor–acceptor (D–A) TADF materials rely on a strongly twisted architecture to enable the required spatial separation of the HOMO and LUMO that governs the magnitude of the singlet‐triplet energy gap (Δ*E*_ST_), when this separation is sufficiently large, thermally promoted reverse intersystem crossing (RISC) of dark triplets to emissive singlets is enabled, resulting in up to 100% internal quantum efficiency in the device. However, the photoluminescence (PL) spectra of conventional D–A type TADF emitters are broad, reflected in the large full‐width‐at‐half‐maximum (FWHM) that is normally greater than 70 nm. This leads to poor color purity in the device and occurs because of the relatively large structural relaxation in the excited state associated with its long‐range charge transfer (LRCT) character. Multiresonant TADF (MR‐TADF) compounds provide a solution to this issue. These p‐ and n‐doped polycyclic aromatic hydrocarbons, first introduced by Hatakeyama and coworkers [3], typically are rigid structures that emit from short‐range charge transfer (SRCT) excited‐states, resulting in narrowband PL spectra and TADF. However, most MR‐TADF emitters have a moderately large Δ*E*_ST_ (typically around 150 meV) [4, 5], and this engenders a relatively slow *k*_RISC_, which produces a large accumulation of triplet excitons at high current densities in the device that leads to severe efficiency roll‐off and poor device stability.

According to Fermi's golden rule, *k*_RISC_ is dependent on both the spin‐orbit coupling (SOC) between singlet and triplet excited states and the Δ*E*_ST_. To accelerate *k*_RISC_ in MR‐TADF compounds, one of the most popular strategies is to embed heavy p‐block atoms within the structure, exploiting the heavy atom effect to strengthen the SOC between singlet and triplet states [6]. Park et al. first explored this strategy in a study of three emitters **CzBO**, **CzBS**, and **CzBSe** that contain embedded oxygen, sulfur, and selenium atoms, respectively. With an increase in the atomic number (*Z*), the SOC is enhanced and the *k*_RISC_ accelerates significantly from 0.9 × 10^4^ s^−1^ for **CzBO** to 22 × 10^4^ s^−1^ for **CzBS** and 18,000 × 10^4^ s^−1^ for **CzBSe**. The device EQE_max_ correspondingly increased from 13.4% to 23.1% and 23.9%, while the EQEs at 1000 cd m^−2^ (EQE_1000_) were 3.5%, 15.0%, and 20.0%, respectively, demonstrating progressively attenuated efficiency roll‐off [7]. Subsequently, many examples have been reported and summarized [8, 9]. Very few of these examples emit at the targeted blue region to meet the BT. 2020 color point (CIE_y_ = 0.04), and despite the rather milder efficiency roll‐off of devices using this class of MR‐TADF emitter, device stability is either not addressed or remains poor. This is likely a result of the relatively weak C─S and C─Se bonds contained within many of these compounds that serve as points of degradation.

Another strategy to enhance SOC would be to design compounds that have low‐lying n‐π* triplet excited states as these, according to EI Sayed's rule, can also enhance the SOC with a SRCT S_1_ state [10]. Suitably designed hybrid carbonyl‐ and boron‐based MR‐TADF emitters may respond to this design strategy. There are to date very few examples of this class of MR‐TADF emitter, some of which have reported *k*_RISC_ on the order of 10^5^ s^−1^. One of the first examples of these hybrid MR‐TADF emitters is **DOBDiKTa**, reported by our group, which emits at 445 nm in toluene and has a *k*_RISC_ of 8.8 × 10^4^ s^−1^, a value that is 3.5 times faster than that of **DiKTa** (*k*_RISC_ = 2.5 × 10^4^ s^−1^). The moderate *k*_RISC_ of **DOBDiKTa** was attributed to its relatively large Δ*E*_ST_ of 0.21 eV in toluene. Given its *Φ*_PL_ of 75%, the device EQE_max_ was 17.4% at the CIE coordinates were (0.14, 0.12), making it, at that time, the bluest device employing a ketone‐containing MR‐TADF emitter [11]. **h‐BNCO‐1**, a derivative of **BNCZ** having fused carbonyl groups, emits at *λ*_PL_ of 516 nm (FWHM of 28 nm) in toluene and has a miniscule Δ*E*_ST_ of 0.03 eV, resulting in a *k*_RISC_ of 1.79 × 10^5^ s^−1^. As a result, the device EQE_max_ was high and the efficiency roll‐off was small (EQE_max/1000_ of 40.1/34.6%) [12]. The monocarbonyl compound **TCZBAO** (*λ*_PL_ = 507 nm) has a blue‐shifted emission in toluene compared to ***h*‐BNCO‐1**, but has a larger Δ*E*_ST_ of 0.10 eV and slower *k*_RISC_ of 1.6 × 10^5^ s^−1^. Thus, the devices showed EQE_max/1000_ of 36.1/29.2% [13]. Cheng et al. reported the green emitter ***h*‐BNCO‐2** and the red emitter ***h*‐BNCO‐3**, respectively, emitting at *λ*_PL_ of 506/582 nm and having small Δ*E*_ST_ of 0.06/0.07 eV in toluene. Compared to the EQE_max/100/1000_ of 26.4/15.6/4.7% for the unsensitized device with ***h*‐BNCO‐3**, the device with ***h*‐BNCO‐2** exhibited milder efficiency roll‐off (EQE_max/100/1000_ of 31.3/28.9/19.7%) due to the faster *k*_RISC_ of 2.99 × 10^5^ s^−1^ of ***h*‐BNCO‐2** than 1.28 × 10^5^ s^−1^ of ***h*‐BNCO‐3** [14]. **BNO**, **BNDO**, and **BNTO** are three structurally related emitters having progressively a greater number of fused ring systems. These three compounds emit at *λ*_PL_ of 477, 489, and 483 nm and have small Δ*E*_ST_ of 0.05, 0.07, and 0.07 eV in toluene, respectively. **BNDO** shows relatively faster *k*_RISC_ of 3.39 × 10^5^ s^−1^, 1.3 times that of **BNTO** (2.65 × 10^5^ s^−1^) and 3.9 times that of **BNO** (8.66 × 10^4^ s^−1^). The corresponding OLED with **BNDO** showed an EQE_max/100/1000_ of 19.0%/13.3%/10.4% [15]. **CO‐BCzBN** and **CO‐DABNA** are two conformationally locked hybrid MR‐TADF compounds with a carbonyl group positioned *para* to one of the nitrogen atoms within the MR‐TADF core. Compared to their parent compounds **BCz‐BN** and **t‐DABNA** (*λ*_PL_ = 467 and 444 nm, respectively), **CO‐BCzBN** and **CO‐DABNA** have a narrower, blue‐shifted emission at *λ*_PL_ of 453 and 436 nm (FWHM of 16 and 18 nm) in toluene yet the same Δ*E*_ST_ of 0.22 eV in 1 wt% doped film in PhCzBCz. The devices with **CO‐BCzBN** and **CO‐DABNA** showed EQE_max/100/1000_ of 35.1%/18.0%/7.0% and 8.3/2.0/‐%, respectively. The severe efficiency roll‐off is caused by the slow *k*_RISC_ (ca. 10^4^ s^−1^). Notably, the simple late‐stage, one‐step carbonylation reaction broadened the horizon for the introduction of carbonyl groups in MR‐TADF compounds [16]. This survey reveals the beneficial role of the incorporation of carbonyl groups in accelerating RISC, and that there is only a single example of a boron‐carbonyl hybrid MR‐TADF compound emitting in the targeted blue (CIE_y_ < 0.1) region; however, the device with **CO‐DABNA** suffers from severe efficiency roll‐off, showing an EQE_100_ of only 2.0%.

Examples of blue‐emitting heavy atom‐free boron‐based MR‐TADF compounds with extended π‐frameworks that also show fast *k*_RISC_ (i.e., *k*_RISC_ > 10^5^ s^−1^) do exist. Examples such as ***v*‐DABNA**, ***f*‐DOABNA**, **DOB2‐DABNA‐A‐NP**, and **DOB2‐DABNA‐B‐NP** that emit at *λ*_PL_ of 469, 445, 446, and 471 nm (FWHM of 18, 18, 29, and 28 nm) have *k*_RISC_ of 2.0 × 10^5^, 2.3 × 10^6^ s^−1^, 1.2 × 10^6^, and 3.8 × 10^5^ s^−1^, respectively, correlated broadly with their small Δ*E*_ST_ of 0.017, 0.08, 0.018, and 0.0065 eV [17, 18, 19]. All these emitters have in common extended π‐frameworks. The OLEDs showed EQE_max_ of 34.4%, 19.9%, 24.1%, and 29.1%, respectively, while the EQE_1000_ were 26.0%, 6.7%, 21.6%, and 24.0%. The tetraboron emitter **tBO‐4B** (*λ*_PL_ = 449 nm in toluene) likewise has a negligible Δ*E*_ST_ of 1 meV, fast *k*_RISC_ of 4.23 × 10^6^ s^−1^. OLEDs with this emitter show outstanding performance with EQE_max/1000_ of 39.6%/31.5% [20]. A synergistic strategy of π‐extension and heavy‐atom incorporation was invoked by Lin et al. for **DBDSe** [21]. This compound emits at 453 nm and has Δ*E*_ST_ and *k*_RISC_ of 0.17 eV and 3.0 × 10^6^ s^−1^. The device showed EQE_max/1000_ of 40.5%/28.2% at CIE coordinates of (0.140, 0.059).

**FIGURE 1:**
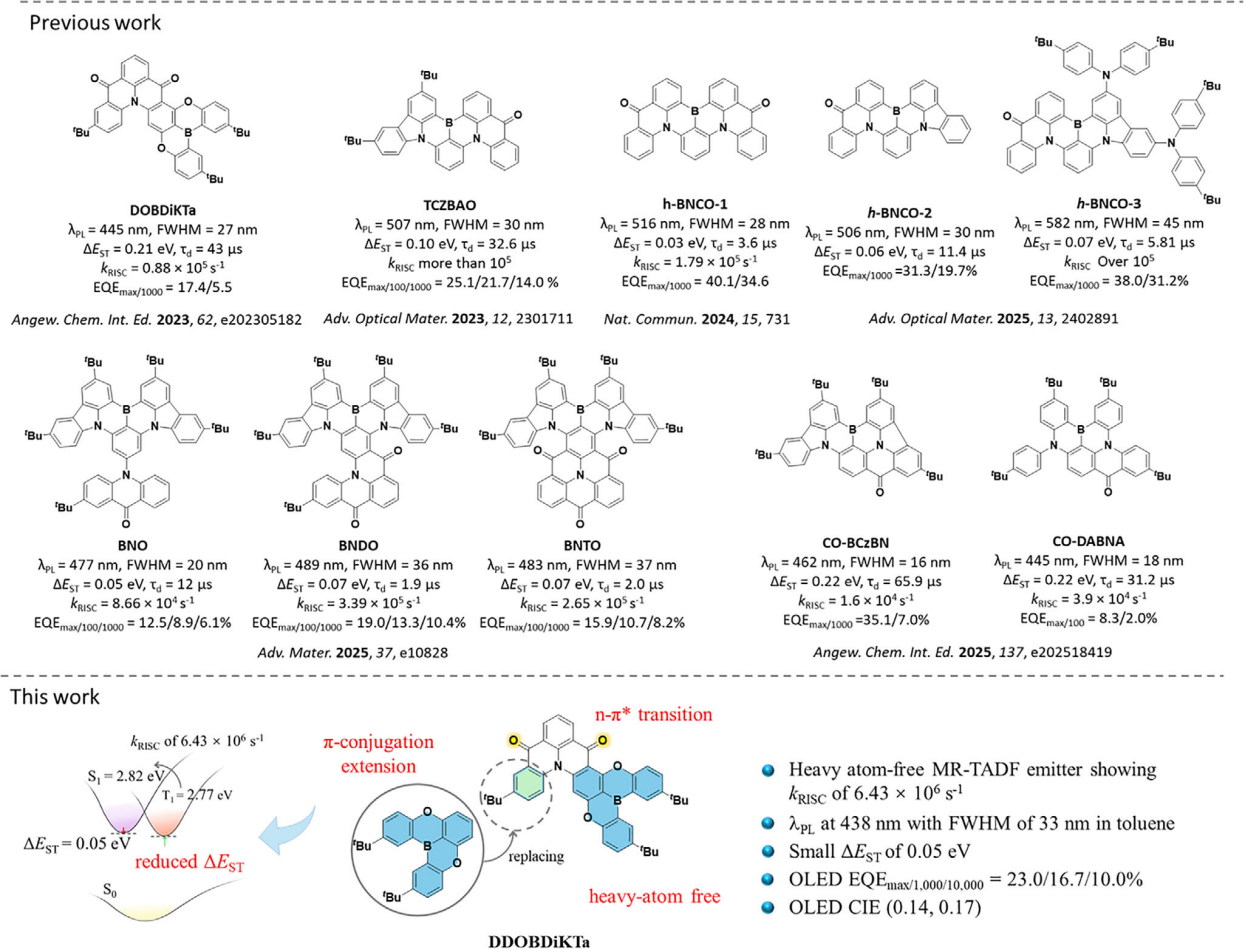
Examples of boron‐, carbonyl‐ hybrid MR‐TADF emitters and a summary of the emitter design in this work.

Herein, we present an emitter design strategy that synergistically combines hybrid carbonyl‐ and boron‐based n‐dopants with an extended π‐framework within a single MR‐TADF compound. **DDOBDiKTa** shows narrowband blue emission and fast RISC (Figure 1). The PL spectrum of **DDOBDiKTa** peaks at *λ*_PL_ of 438 nm and has a small FWHM of 33 nm in toluene, blue‐shifted compared to the unsymmetric analog **DOBDiKTa** (*λ*_PL_ = 445 nm) [11]. The PL spectrum of the 1.5 wt% doped film of **DDOBDiKTa** in a 1:1 mCP:PPT cohost is red‐shifted to 451 nm. Due in part to the very small Δ*E*_ST_ of 0.05 eV, the *k*_RISC_ is exceptionally fast for a MR‐TADF emitter at 3.22 × 10^6^ s^−1^. The 5 wt% doped film in 26DCzPPy also shows fast RISC (*k*_RISC_ = 1.98 × 10^6^ s^−1^). In fact, to the best of our knowledge, **DDOBDiKTa** shows one of the fastest *k*_RISC_ of MR‐TADF emitters whose structure does not contain heavy atoms. With a *Φ*_PL_ of 70%, the resulting EQE_max​_ is relatively lower than that of some of the examples cited above. However, the OLEDs exhibited quite mild efficiency roll‐off, achieving an EQE_max_ of 23.0% and an EQE at 10,000 cd m^−2^ of 10.0%; notably, just a handful of MR‐TADF OLEDs reach this high luminance.

## 2 Results and Discussion

### 2.1 Synthesis and Chemical Characterization

**FIGURE 2:**
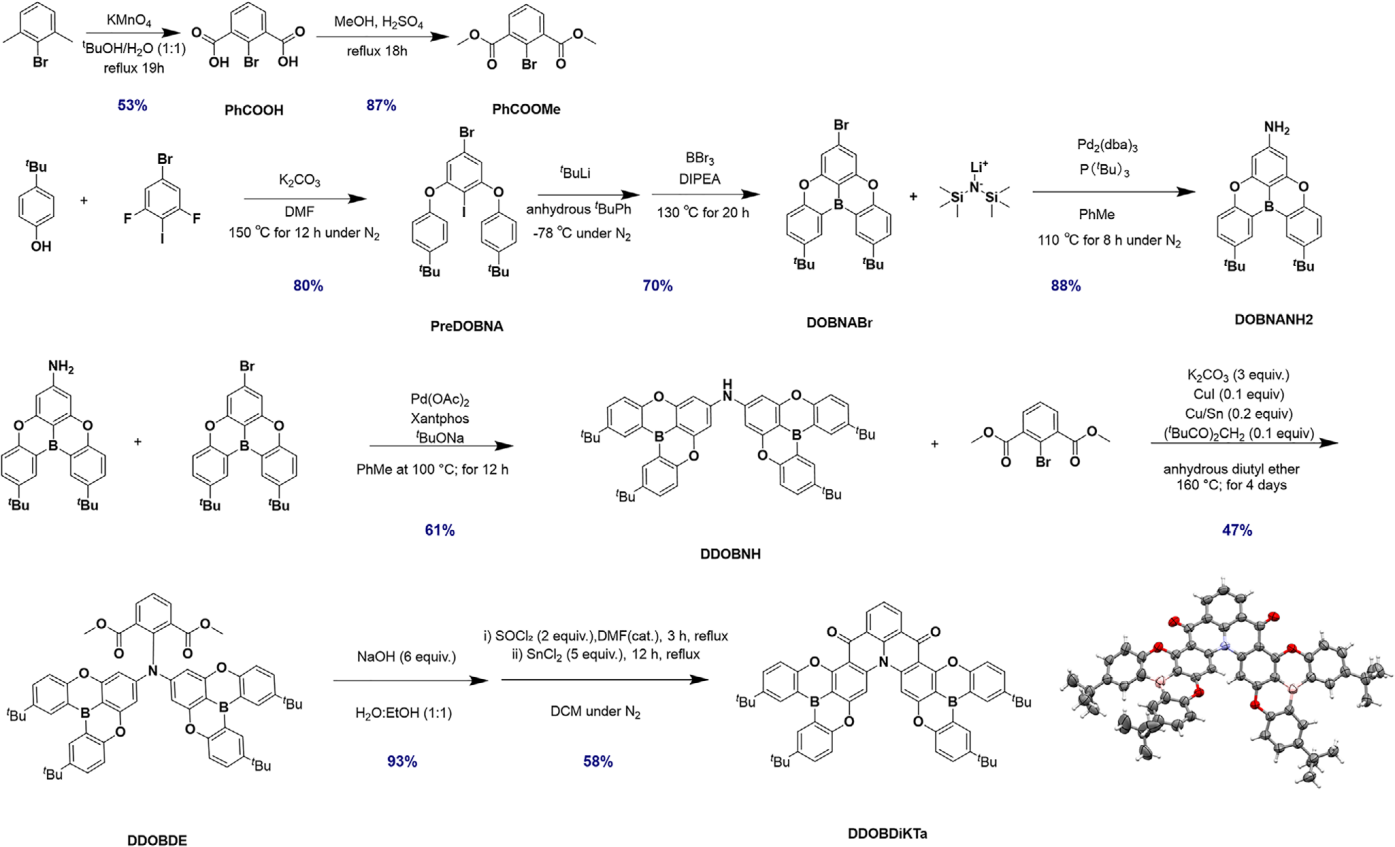
Synthetic route to **DDOBDiKTa**, including a view of a single independent molecule from its crystal structure of (ellipsoids drawn at 50% probability and minor part of disorder omitted for clarity).

The synthetic route to **DDOBDiKTa** is shown in Figure 2. Dimethyl 2‐bromoisophthalate was obtained by a sequential oxidation and esterification of 2‐bromo‐1,3‐dimethylbenzene. **DOBNABr** was synthesized via a sequence of nucleophilic aromatic substitution and borylation reactions. The key intermediate **DOBNANH_2_** was obtained from the known compound **DOBNABr** using lithium bis(trimethylsily)amide (LiHMDS) in a yield of 88%. This was then elaborated in a four‐step sequence to **DDOBDiKTa** in 16% overall yield (see Figures S16–S24 for compound characterization). Thermal gravimetric analysis (TGA) measured under nitrogen reveals that **DDOBDiKTa** has a high thermal stability with a 5 wt% loss temperature of 487°C (Figure S25).

Crystals of **DDOBDiKTa** suitable for x‐ray diffraction were obtained following slow evaporation of an *ortho*‐dichlorobenzene/methanol solution. The structure contains three independent molecules of **DDOBDiKTa**, (Figure S36), which all exhibit a slightly distorted molecular conformation with dihedral angles of 44.47(12)°, 48.79(12)°, and 44.11(13)° across the DiKTa moiety, respectively (Figure S37). The molecules stack together into an interlaced ABB, head‐to‐tail‐to‐tail, unit (Figure S38) with distances (centroid⋯centroid) of 3.399–3.723 Å between neighboring **DOBNA** moieties. The ABB units form additional, more offset, stacking interactions with adjacent ABB units, giving repeating ABB·BBA patterns along the [01‐1] direction through both π⋯π (centroid⋯centroid 3.678–3.897 Å) and C═O⋯π (centroid⋯centroid 3.083 Å) interactions (Figure S39).

### 2.2 Theoretical Modelling

**FIGURE 3:**
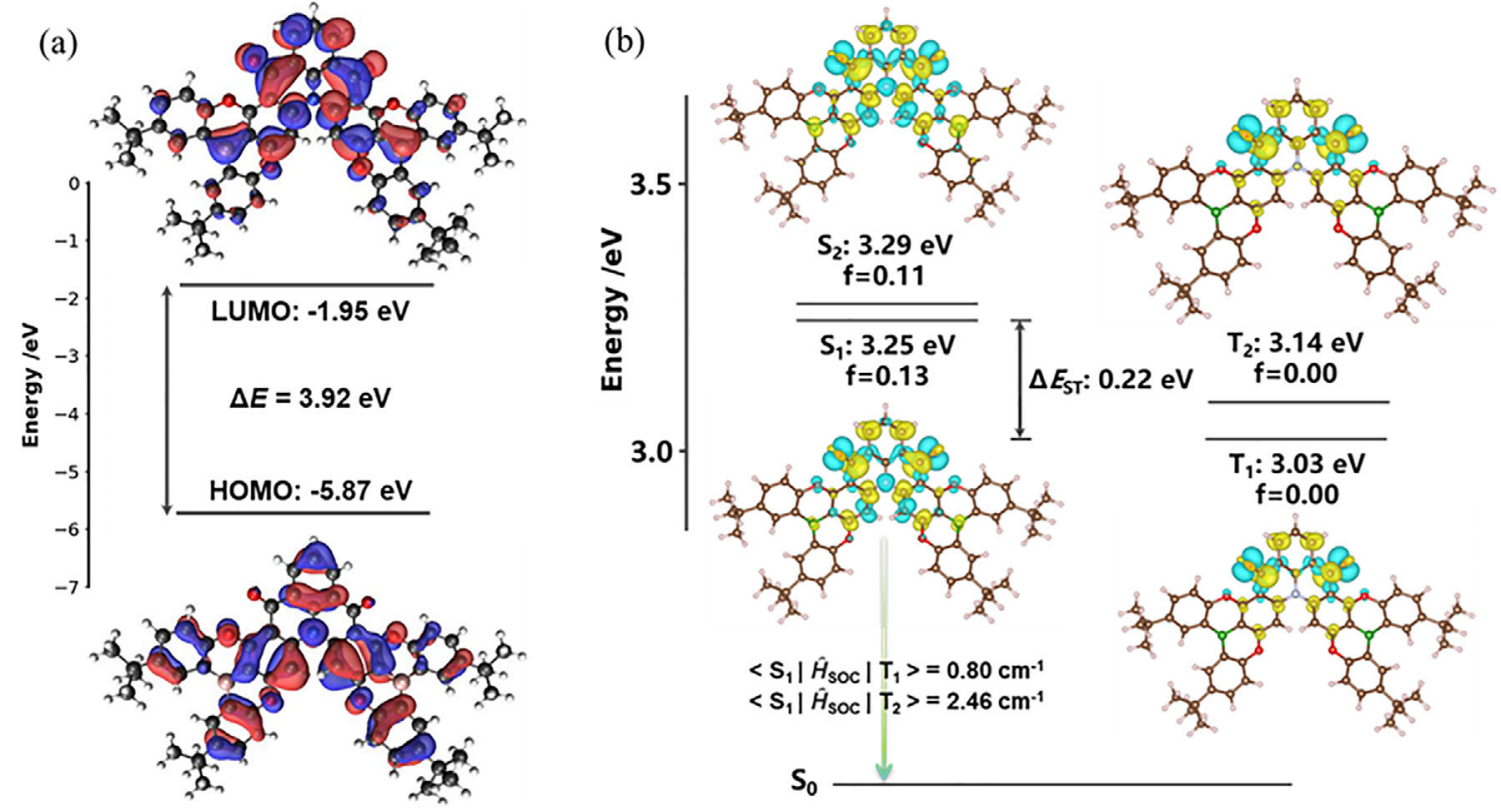
Distribution for FMOs of DDOBDiKTa, calculated at the (a) PBE0/6‐31G(d,p) level and (b) difference density plots of S_1_ and T_1_ excited states, calculated at the SCS‐ADC(2)/cc‐pVDZ level for DDOBDiKTa (SOCME values based on the optimized T_1_ geometry in the gas phase at PBE0/6‐31G(d,p)).

A combination of DFT and wavefunction‐based calculations provides insight into the structural and optoelectronic properties of this compound (see Supporting Information for details). As shown in Figure 3, the highest occupied molecular orbital (HOMO) is distributed over the whole skeleton, while the lowest unoccupied molecular orbital (LUMO) is mainly located on the central **DiKTa** unit and extends to the boron atoms, aligning with the more stabilized LUMO of **DiKTa** compared to **DOBNA**. Indeed, the calculated HOMO and LUMO levels of **DDOBDiKTa** are −5.87 and −1.95 eV, respectively, which are deeper than those of **DOBNA** (HOMO: −5.68 eV, LUMO: −1.71 eV), shallower than those of **DiKTa** (HOMO: −6.20 eV, LUMO: −2.23 eV) and **DOBDiKTa** (HOMO: −5.95 eV, LUMO: −2.03 eV) [11]. In the singlet excited state, the electron density is localized on the **DiKTa** unit and shows the expected alternating increasing and decreasing distribution that reflects their SRCT character. The nature of the triplet state is n–π* local excitation on the carbonyl fragment. The S_1_ and T_1_ energies are calculated to be 3.25 and 3.03 eV, respectively, thus the Δ*E*_ST_ is 0.22 eV, smaller than **DiKTa** (0.26 eV) and slightly higher than that of **DOBNA** (0.21 eV) and **DOBDiKTa** (0.19 eV). The measured Δ*E*_ST_ is much smaller (vide infra) than expected based on the coupled cluster calculations (see also Figure S28a); however, when the state energies were computed at the RI‐wPBEPP86/cc‐pVDZ level in the gas phase, the predicted Δ*E*_ST_ of 0.07 eV is closer to the measured values, but this comes at the expense of the S_1_ and T_1_ levels being unrealistically overestimated (S_1_: 3.51 eV; T_1_: 3.58 eV). A large oscillator strength is correlated to a fast‐radiative rate constant and, thus, a high *Φ*_PL_. The oscillator strength for the S_0_‐S_1_ transition is 0.13, implying a more moderate *Φ*_PL_ compared to a computed value of 0.30 for **DOBDiKTa** [11]. The calculated spin‐orbit coupling matrix element (SOCME) based on the T_1_ optimized geometry of **DDOBDiKTa** between S_1_ and T_1_ is 0.79 cm^−1^ (Figure S28b), while the SOCME values between S_1_ and the six next higher‐lying triplet excited states range from 1.14 to 2.52 cm^−1^. Especially, the larger <S_1_|*Ĥ*_SOC_|T_2_> value of 2.52 cm^−1^ is attributed to an n‐π* transition localized on the carbonyl groups. These closely lying intermediate triplet states (T_3‐7_) may participate in the RISC process between T_1_ and S_1_ mediated by spin‐vibronic coupling.

The energies of the frontier molecular orbitals were inferred from electrochemical measurements in degassed DCM. The electrochemical data, reported versus SCE, are summarized in Table S1. As shown in Figure S27, both the first oxidation and reduction waves are irreversible, with associated peak potentials *E*_ox_ and *E*_red_ from the differential pulse voltammogram (DPV) of 1.70 and −1.56 eV, respectively. The corresponding HOMO and LUMO levels are −6.04 and −2.78 eV, respectively, meaning that the HOMO‐LUMO energy gap, Δ*E*_H‐L_ is 3.26 eV. The HOMO is shallower while the LUMO is deeper compared to the calculated results. **DDOBDiKTa** has a larger Δ*E*_H‐L_ compared to **DOBDiKTa** (HOMO: −5.78 eV, LUMO: −2.80 eV, Δ*E*_H‐L_: 2.98 eV), indicating a likely blue‐shifted emission.

### 2.3 Photophysical Investigation

**FIGURE 4:**
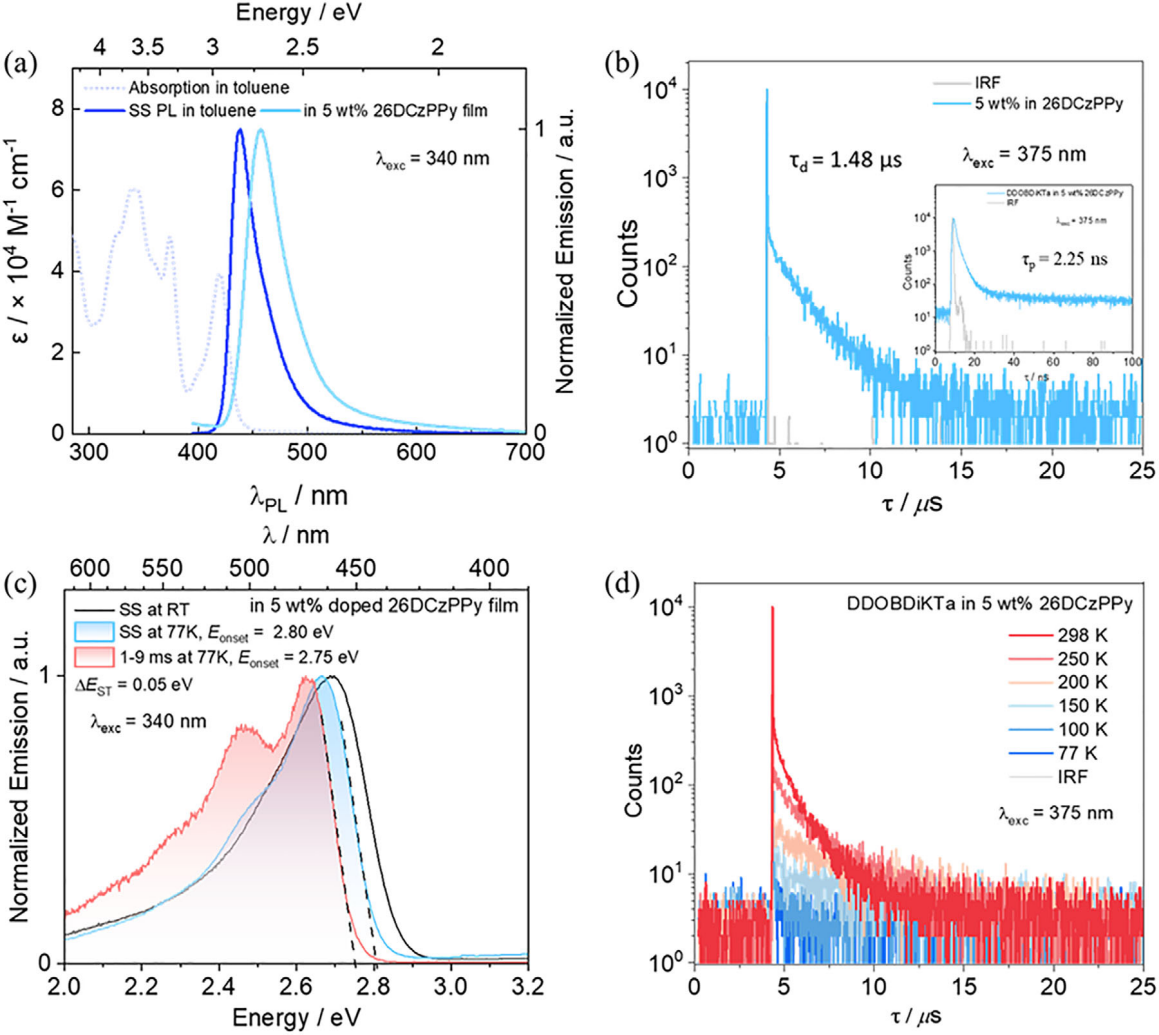
a) UV–vis absorption and photoluminescence spectra in dilute toluene (10^−5^ M) and 5 wt% doped film in 26DCzPPy, *λ*_exc_ = 340 nm; b) Time‐resolved PL decay of 5 wt% doped film in 26DCzPPy (*λ*_exc_ = 375 nm) at room temperature. c) Prompt PL and delayed emission spectra measured in 5 wt% doped film in 26DCzPPy at 77 K (*λ*_exc_ = 340 nm). d) Temperature‐dependent TRPL decays in 5 wt% doped film in 26DCzPPy (*λ*_exc_ = 375 nm).

Photophysical measurements were first conducted in toluene solution (10^−5^ M) to elucidate the photophysical properties of monomolecular **DDOBDiKTa**. As shown in Figure 4a, the absorption spectrum of **DDOBDiKTa** has three distinct intense bands peaking at *λ*_abs_ of 340, 374, and 420 nm, with molar absorptivity values (*ε*) of 6.03 × 10^4^, 4.85 × 10^4^, and 3.94 × 10^4 ^M^−1^ cm^−1^, respectively. The broad absorption band at 340 nm is assigned to a family of π‐π* transitions. The absorption band at 374 nm is assigned to the SRCT transition localized on the **DOBNA** moieties based on a cross‐comparison with the SRCT band of **DOBNA** (*λ*_PL_ = 383 nm). The lowest energy absorption band at 420 nm is the SRCT transition localized on the **DiKTa** moiety, given that the SRCT band of **DiKTa** is at 433 nm [11].

Under an excitation at 340 nm, **DDOBDiKTa** emits in the deep‐blue at *λ*_PL_ of 438 nm and having a narrow FWHM of 33 nm. The corresponding CIE coordinates are (0.15, 0.05). Due to its rigid structure, **DDOBDiKTa** shows a small Stokes shift of 18 nm (0.12 eV), indicating a relatively weak structural relaxation in the excited state compared to the ground state (Figure S34a). There is only a small degree of positive solvatochromism in the PL spectra, reflecting the SRCT character of the emissive excited state that identifies **DDOBDiKTa** as MR‐TADF (Figure S33). The S_1_ and T_1_ energy levels are 2.75 and 2.69 eV, measured from the onsets of the steady‐state PL and time‐gated phosphorescence at 77 K in 2‐MeTHF in Figure S30b, respectively. The corresponding Δ*E*_ST_ is 0.06 eV, which is very small for MR‐TADF emitters, and thus fast RISC is expected for this compound. Time‐resolved PL measurements in degassed toluene are shown in Figure S29b. The transient decays show an obvious quenching of the delayed component of the PL in air. The prompt and delayed PL lifetimes, *τ*_p_ and *τ*_d_, are 0.77 ns and 1.54 µs in degassed toluene solution, both shorter than that of **DOBDiKTa** (*τ*_p_ and *τ*_d_, 1.9 ns and 11 µs). The exciton is quenched in air where the *Φ*_PL_ decreases from 30% to 9%. From these measurements, the *k*_RISC_ in toluene for **DDOBDiKTa** is 6.43× 10^6^ s^−1,^ which is one‐to‐two orders of magnitude faster than that measured for the other reported boron/carbonyl hybrid emitters in Figure 1.

Given the promising photophysical properties in toluene, we next explored the photophysical properties of **DDOBDiKTa** doped as thin films in two different host matrices; a 1:1 cohost mixture of 1,3‐bis(*N*‐carbazolyl)benzene and 2,8‐bis(diphenylphosphoryl)‐dibenzo‐[b,d]thiophene (*m*CP:PPT), and 2,6‐bis(3‐(carbazol‐9‐yl)phenyl)pyridine (26DCzPPy). These host matrices were chosen as they provide balanced charge transport due to their bipolar character and have suitably high triplet energies [22]. Compared to the film in 26DCzPPy, the film of the 1.5 wt% doped film of **DDOBDiKTa** in the *m*CP:PPT (1:1) cohost has a higher *Φ*_PL_ of 73%. A concentration screen revealed that 1.5 wt% doping for the cohost and 5 wt% doping in 26DCzPPy produced the highest *Φ*_PL_ values of 73% and 70% as shown in Table S3 for *m*CP:PPT and Table S4 for 26DCzPPy, respectively. **DDOBDiKTa** shows blue emission at 451 nm with a FWHM of 36 nm in the cohost film (Figure S29a), while the PL is slightly redshifted to 457 nm for the 5 wt% doped film in 26CzPPy. These thin film emission spectra are red‐shifted compared to that in toluene, likely due to a combination of interactions between emitter and hosts, and the host having a higher polarity than toluene (Figures 4a and S29a). The S_1_ and T_1_ energies obtained from the high‐energy onsets of the SS PL and time‐gated phosphorescence at 77 K are 2.82 and 2.77 eV for doped cohost film (Figure S30a), and 2.80 and 2.75 eV for the doped film in 26DCzPPy (Figure 4c). Regardless of host matrix choice, the estimated Δ*E*_ST_ is 0.05 eV, similar to that measured in 2‐MeTHF (0.06 eV). However, we note an unexpected large discrepancy between the calculated (at the ADC(2) level) and measured Δ*E*_ST_. We thus recalculated the Δ *E*_ST_ of **DDOBDiKTa** and all reported hybrid MR‐TADF emitters at both the ADC(2) and CC2 levels. The results are summarized in Figure S28a. It can be seen that for most of the compounds, regardless of the level of theory, the computed Δ*E*_ST_ matches well the experimental results; however, the Δ *E*_ST_ of symmetric compounds like **DDOBDiKTa** and **h‐BNCO‐1** and **BNTO** are predicted to be much larger than their measured values. So, the discrepancy between theory and experiment seems to arise due to the higher symmetry of these structures. Time‐resolved PL measurements reveal biexponential decay kinetics, with associated prompt, *τ*_p_, and delayed, *τ*_d_, lifetimes of 1.9 ns and 1.88 µs for the film in the cohost, and 2.3 ns and 1.48 µs for the film in 26DCzPPy, respectively. Thus, the exciton dynamics do not seem to be sensitive to the nature of these two hosts. The excitonic kinetics data are summarized in Table 1. A very fast *k*_RISC_ of 3.22 ×10^6^ s^−1^ was determined for **DDOBDiKTa**; one of the fastest *k*_RISC_ values among all the reported MR‐TADF emitters that do not contain any heavy atom (see Figure S35 and Table S6). The fast *k*_RISC_ originates from a synergy of strong SOC, small reorganization energy, *λ*, of 29.9 meV (Figure S28c) between S_1_ and T_1_ and very small Δ*E*_ST_ resulting from an expansion of the π‐system compared to **DOBDiKTa**. Temperature‐dependent TRPL measurements of the 5 wt% doped film in 26DCzPPy reveal the expected TADF behavior (Figure 4d). Given the fast *k*_RISC_, we hypothesized that T_2_ may mediate RISC as there is a stronger computed SOC between T_2_ and S_1_. However, the measured activation energy for RISC obtained from an Arrhenius analysis (Figure S34b) is 58 meV, which is very similar to the measured Δ*E*_ST_. This strongly suggests direct T_1_ to S_1_ RISC.

**TABLE 1:** Photophysical data.

Compound	Medium	*λ*_Abs_ ^c^_/_nm	λ_PL_ ^d^/nm	FWHM^e^/nm	*E*_S1_ ^f^/eV	*E*_T1_ ^f^_/_eV	*∆E*_ST_ ^h^/eV	*Φ*_PL_ ^i^/%	*τ*_p_ ^j^/ns	*τ*_d_ ^j^/µs	k_ISC_ ^k^/s^−1^	k_RISC_ ^k^/s^−1^	k_s_r_ ^k^/s^−1^	k_s_nr_ ^k^/s^−1^
**DDOBDiKTa**	PhMe.^a^	340	438	33	2.75	2.69	0.06	30	0.77	1.54	1.26×10^9^	6.43×10^6^	3.67×10^7^	4.68×10^5^
	mCP:PPT(1:1)^b^	∖	451	36	2.82	2.77	0.05	73	1.9	1.88	4.58×10^8^	3.22×10^6^	5.98×10^7^	1.60×10^5^
	26DCzPPy^b^	∖	457	41	2.80	2.75	0.05	70	2.3	1.48	3.50×10^8^	1.98×10^6^	9.41×10^7^	2.57×10^5^

^a^ In PhMe solutions (10^−5^ M).

^b^ Measured in thin films consisting of either 1.5 wt% emitter in mCP:PPT (1:1) or 5 wt% emitter in 26DCzPPy.

^c^ Lowest energy absorbance peak.

^d^ Steady‐state emission maximum at 300 K. *λ*_exc_ = 340 nm.

^e^ Full width at half maximum of the emission peak.

^f^ S_1_ and T_1_ energies were obtained from the onsets of the respective prompt fluorescence SS PL and phosphorescence spectra (delay: 1 ms; gate time: 9 ms) at 77 K. *λ*_exc_ = 375 nm.

^g^ Solution samples for Δ*E*_ST_ measurements were prepared in 2‐MeTHF (10^−6^ M).

^h^ Δ*E*_ST_ = *E*(S_1_)—*E*(T_1_).

^i^ Relative *Φ*_PL_ in solutions were measured by a comparative method using quinine sulfate as a standard (*Φ*_r_ = 54.6% in 1 N H_2_SO_4_), while absolute *Φ*_PL_ of the thin films were measured using an integrating sphere.

^j^ Prompt and delayed lifetimes were obtained by TCSPC. λ_exc_ = 375 nm.

^k^ Intersystem and reverse intersystem crossing rates were calculated using the steady‐state approximation method as described in Table S5.

### 2.4 Organic Light‐Emitting Diodes

**FIGURE 5:**
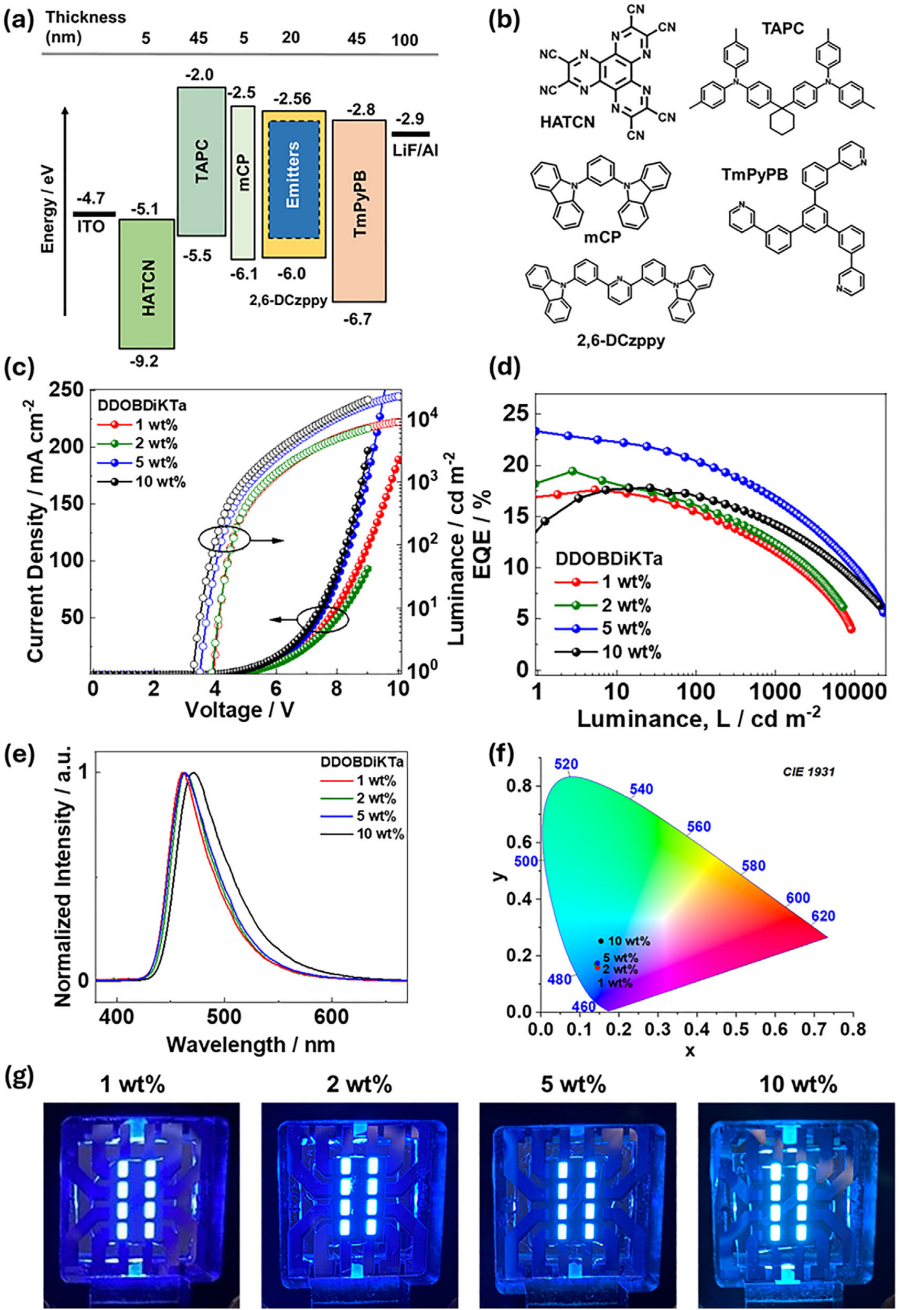
a) Schematic of the OLED stack; b) chemical structures of the materials used in the device; c) current density‐voltage‐luminance (JVL) characteristics; d) EQE versus luminance; e) electroluminescence spectra; f) CIE coordinates of the devices; g) photographs of OLEDs with 1–10 wt% of **DDOBDiKTa** as an emitter.

The promising photophysical properties of the **DDOBDiKTa** encouraged the fabrication of OLEDs. The device architecture and the chemical structures of the organic layers are shown in Figure 5a,b, respectively. The OLED structure consisted of: indium tin oxide (ITO)/1,4,5,8,9,11‐hexaazatriphenylenehexacarbonitrile (HATCN, 5 nm)/1,1‐bis[(di‐4‐tolylamino)phenyl]cyclohexane (TAPC, 45 nm)/1,3‐bis(N‐carbazolyl)benzene (mCP, 5 nm)/emission layer (EML) consisting of 26DCzPPy as host (20 nm) and **DDOBDiKTa** as the emitter dopant/1,3,5‐tris(3‐pyridyl‐3‐phenyl)benzene (TmPyPB, 45 nm)/lithium fluoride (LiF, 1 nm)/aluminum (Al, 100 nm). Here, HATCN was used as the hole injection layer, TAPC as the hole transport layer, mCP as an exciton blocking layer, TmPyPB as electron transport layer, and LiF to reduce the work function of the top Al electrode. OLEDs were made with various concentrations of the emitter (1, 2, 5, and 10 wt%). Figure 5c shows the current density‐voltage‐luminance (JVL) characteristics of the devices. OLEDs with low concentrations of emitter (1 and 2 wt%) showed a turn‐on voltage of around 4 V while it was less (3.3–3.5 V) for high concentrations (5 and 10 wt%). The current density of OLEDs at a similar voltage was higher for OLEDs with high concentrations of the emitter than for devices with low emitter concentration. Similarly, higher luminance was demonstrated by the high concentration devices at similar voltages. This improved charge injection, higher luminance and lower turn‐on voltage with an increase in concentration (> 2 wt%) of the **DDOBDiKTa** emitter indicates that the charges were injected directly onto the emitter in addition to the host. This increased charge injection (high current density) contributes to higher efficiency by enhanced recombination. Figure 5d and Table 2 show the EQE values of the OLEDs with various concentrations of the emitter. The device with 1 wt% of emitter dopant showed moderate EQE_max/100//1000_ of 17.6/15.3/11.5% at *λ*_EL_ of 462 nm. The full‐width half maximum (FWHM) of the OLED is 45 nm and CIE (x,y) coordinates are (0.144, 0.158) close to the deep‐blue region. With an increase in concentration to 2 wt%, an increase in efficiency was also measured: EQE_max/100//1000_ of 19.4/16.1/12.3%. The *λ*_EL_ of the 2 wt% OLEDs is very similar (463 nm), FWHM is 45 nm and CIE (x, y) coordinates are (0.141, 0.170).

**TABLE 2:** Device performance of OLEDs with 1, 2, 5, and 10 wt% of **DDOBDiKTa**.

Conc./wt%	EQE_max_/%	EQE_100/1000/10,000_/%	*L*_max_/cd m^−2^	*λ*_EL_/nm	FWHM/nm	CIE/x, y
**1**	**17.6**	**15.3/11.5/NA**	**9080**	**462**	**45**	**0.144, 0.158**
**2**	**19.4**	**16.1/12.3/NA**	**7151**	**463**	**45**	**0.141, 0.170**
**5**	**23.0**	**20.4/16.7/10.0**	**23,200**	**463**	**49**	**0.144, 0.174**
**10**	**17.8**	**16.9/14.2/8.8**	**20,523**	**472**	**55**	**0.154, 0.252**

The 5 wt% OLEDs showed the best efficiencies among all devices with an impressive EQE_max_ of 23.0% and a mild roll‐off with EQE_100/1000/10,000_ of 20.4/16.7/10.0%. The OLEDs were measured up to a maximum brightness (*L*_max_) of 23,200 cd m^−2^ where the EQE at this brightness was 5.6%. The remarkable EQE_max_ of devices containing 5 wt% of the **DDOBDiKTa** emitter can be attributed to a combination of adequate *Φ*_PL_ of 70% (Table 1) and the favorable balanced charge injection characteristics observed by the superior JVL trends of these devices. The improved charge injection into the EML also decreases the accumulation of charges at the EML/blocking layer interface, thus reducing the undesirable processes such as singlet‐polaron and exciton‐polaron quenching [23]. The mild roll‐off is attributed to the combined effect of fast decay kinetics, with short delayed *τ*_d_ (1.48 µs) in addition to the very fast *k*_RISC_ of 1.98 ×10^6^ s^−1^. These desirable properties contribute to a reduction in singlet and triplet population, which consequently decreases the probability of annihilation processes such as singlet‐triplet, triplet‐triplet, and triplet‐polaron, leading to a less‐pronounced efficiency roll‐off in the OLEDs [2]. This aligns with a high figure‐of‐merit (FOM) reported for TADF emitters where these photophysical properties were correlated with low efficiency roll‐off in OLEDs [24]. We estimated the FOM for **DDOBDiKTa** emitter compared to reported emitters with similar designs using the relation $\;\frac{4k_{r}^{S}k_{RISC}}{3k_{r}^{S}+4k_{ISC}}$ (Table 3). The *λ*_EL_ (463 nm) of the 5 wt% OLEDs was similar to that of devices with 2 wt% however, FWHM (49 nm) was slightly broader (by 4 nm). The lack of *λ*_EL_ shift and slightly wider FWHM indicates broadening of the recombination zone. This also aligns with the claim of enhanced charge injection into the EML, which improves the charge distribution in the EML, hence positively contributing to the device efficiency. The CIE (*x*, *y*) coordinates of the 5 wt% OLEDs are (0.144, 0.174), similar to the devices at 1 and 2 wt% doping, again close to the deep‐blue region. OLEDs with 10 wt% of **DDOBDiKTa** showed a slightly lower EQE_max_ of 17.8%; however, the EQE_100/1000_ of 16.9%/14.2% was still higher than the device with low doping ratio of emitter (1 and 2 wt%), indicating less‐pronounced efficiency roll‐off at higher concentrations. The *λ*_EL_ of the devices is at 472 nm, FWHM is 55 nm and CIE (*x*,*y*) coordinates are (0.154, 0.252). Figure 5g presents the photographs of the OLEDs using (1–10 wt%) of **DDOBDiKTa** emitter.

**TABLE 3:** Photophysical properties of **DDOBDiKTa** compared with reported emitters to analyze the FOM for efficiency roll‐off.

Emitter	*λ*_PL/_nm	EQE_max_/EQE_100_/EQE_1000_ [%]	*k*_r_ [10^7^]	*k*_ISC_ [10^7^]	*k*_RISC_ [10^3^]	$\frac{\mathrm{EQ}E_{1000}}{\mathrm{EQ}E_{\max}}$[%]	FOM $\left(\frac{4k_{r}^{S}k_{\mathrm{RISC}}}{3k_{r}^{S}+4k_{\mathrm{ISC}}}\right)$
DABNA ‐2 [25]	469	20.2/13.4/NA	14.1	1.01	14.8	NA	18.0
v‐DABNA [17]	467	34.4/32.8/26.0	22	2.3	200	75.5	234
3tPAB [26]	460	19.3/12/4	13.9	10.6	57.4	20.7	37.9
DOBDiKTa [11]	460	17.4/11.8/5.5	6.92	31.5	88.2	31.6	16.6
**DDOBDiKTa**	457	23.0/20.4/16.7	9.41	35.0	1980	72.6	442

## 3 Conclusions

Here, we introduced an emitter design strategy that synergistically incorporates a π‐extended structure containing boron‐ and carbonyl‐ n‐dopants and nitrogen‐ and oxygen‐ p‐dopants. Through their judicious placement, the compound has a very small Δ*E*_ST_ while SOC is enhanced. **DDOBDiKTa** thus shows outstanding photophysical properties, emitting brightly in the deep blue region with CIE coordinates of (0.15, 0.05) in toluene. **DDOBDiKTa** has a high *Φ*_PL_ of 70% and a small Δ*E*_ST_ of 0.05 eV in 5 wt% doped 26DCzPPy film, resulting in *k*_RISC_ of 1.98 ×10^6^ s^−1^, which is one of the fastest *k*_RISC_ values among all the reported MR‐TADF emitters that do not contain heavy atoms. The OLED showed an EQE_max_ of 23.0% and very low efficiency roll‐off, where the EQE remained at 10.0% at 10,000 cd m^−2^. Notably, this cooperative design strategy enables narrowband blue emission accompanied by an exceptionally fast *k*_RISC_, demonstrating a promising molecular engineering paradigm for high‐performance blue MR‐TADF emitters.

## Author Contributions

**Wenrui Wang**: investigation, writing – original draft preparation, review and editing. **Hassan Hafeez**: investigation for devices, writing – review and editing. **Sen Wu**: conceptualization, writing – review and editing. **Aidan P. McKay**: x‐ray crystallography, writing – review and editing. **David B. Cordes**: x‐ray crystallography, supervision, writing – review and editing. **Ifor D. W. Samuel**: project management, supervision, resources, writing – review and editing. **Eli Zysman‐Colman**: project management, supervision, resources, writing – review and editing.

## Conflicts of Interest

The authors declare no conflicts of interest.

## Supporting information




^1^H NMR and ^13^C NMR spectra, GCMS, HRMS, TGA, CV and reverse phase and gel permeation (GPC) HPLC; supplementary computational data and coordinates; Supplementary photophysical and OLED data; equations for kinetics parameters calculation; summary table of *k*_RISC_ values for reported MR‐TADF emitters without heavy atoms; single crystal x‐ray crystallography experimental.**Supporting File 1**: anie73565‐sup‐0001‐SuppMat.pdf.

**Supporting File 2**: anie73565‐sup‐0002‐cif.zip.

## Data Availability

The research data supporting this publication can be accessed at https://doi.org/10.17630/ec438717‐8696‐4379‐a40c‐15aea8efe452. CCDC 2497380 contains the full crystallographic data and can be obtained free of charge from The Cambridge Crystallographic Data Centre via www.ccdc.cam.ac.uk/structures.
