# Supplementary material for: A Blue Emissive Heavy Atom‐Free Multiresonant Thermally Activated Delayed Fluorescent Emitter Shows Ultra‐Fast Reverse Intersystem Crossing
Source: Angew Chem Int Ed Engl. 2026 Jul 13;65(38):e9957319. doi: 10.1002/anie.9957319 (PMC13573146; doi:10.1002/anie.9957319)
Supplement: Supplementary file 1 [file ANIE-65-e9957319-s002.pdf]

# Supplementary Information

# A Blue Emissive Heavy Atom-Free Multiresonant Thermally Activated Delayed Fluorescent Emitter Shows Ultra-fast Reverse Intersystem Crossing

*Wenrui Wang* [a], *Hassan Hafeez* [b], *Sen Wu* [a], *Aidan P. McKay* [a], *David B. Cordes* [a], *Ifor D. W. Samuel* *[b] *and Eli Zysman-Colman* *[a]

[a] Organic Semiconductor Centre, EaStCHEM School of Chemistry, University of St Andrews, St Andrews, UK, KY16 9ST.

[b] Organic Semiconductor Centre, SUPA School of Physics and Astronomy, University of St Andrews, North Haugh, St Andrews, UK, KY16 9SS.

E-mail: eli.zysman-colman@st-andrews.ac.uk, idws@st-andrews.ac.uk,

# Table of Contents

Research methods .................................................................................................... S3

OLED fabrication .................................................................................................... S7

Synthesis .................................................................................................... S8

Photophysical characterization and computations ....................................................S30

Literature study .................................................................................................... S34

X-ray Crystal Structure Figures .................................................................. S37

References .................................................................................................... S39

**Supporting Information**

## 1. Research methods

***General information:*** All reagents and solvents were obtained from commercial sources and used as received. Air-sensitive reactions were performed under a nitrogen atmosphere using Schlenk techniques, no special precautions were taken to exclude air or moisture during work-up and crystallisation. Anhydrous DCM and toluene were obtained from an MBraun SPS5 solvent purification system. Flash column chromatography was carried out using a Teledyne ISCO CombiFlash® NextGen 300+ system with RediSep® Normal-Phase Silica columns in sizes of 4 - 80 g silica packing. Analytical thin-layer-chromatography (TLC) was performed with silica plates with aluminum backings (250 µm with F-254 indicator). TLC visualization was accomplished using a 254/365 nm UV lamp. HPLC analysis was conducted on a Shimadzu LC-40 HPLC system. HPLC traces were performed using a Shim-pack GIST 3 µm/5 µm C18 reverse phase analytical column. GCMS analysis was conducted using a Shimadzu QP2010SE GC-MS equipped with a Shimadzu SH-Rtx-1 column (30 m x 0.25 mm). $^{1}$H and $^{13}$C NMR spectra were recorded on a Bruker Advance spectrometer (400 MHz or 500 MHz for $^{1}$H, 126 MHz for $^{13}$C). The following abbreviations have been used for multiplicity assignments: "s" for singlet, "d" for doublet, "t" for triplet, "dd" for doublet of doublets, "m" for multiplet. $^{1}$H and $^{13}$C NMR spectra were referenced residual solvent peaks with respect to TMS ($\delta$ = 0 ppm). Melting points were measured using open-ended capillaries on an Electrothermal 1101D Mel-Temp apparatus and are uncorrected. High-resolution mass spectrometry (HRMS) was performed by the EPSRC National Mass Spectrometry Service Centre (NMSSC) at the University of Edinburgh Mass Spectrometry Facility. Elemental analyses were performed by Dr. Joe Casillo at the School of Geosciences at the University of Edinburgh.

***Quantum chemical calculations.*** Density functional theory (DFT) and time-dependent DFT calculations using the Tamm-Dancoff approximation (TDA-DFT) were performed using the Gaussian 16 revision C.01 package.[1] The ground-state optimization was carried out using DFT using the PBE0[2] functional, the 6-31G(d,p) basis set,[3] and in conjunction with the D3(BJ)[4] dispersion correction scheme in the gas phase, starting from a structure drawn and optimized using Chem3D[5]. The vertical excited-state calculations were performed using Time-Dependent DFT within the TDA-DFT,[6] with the same functional and basis set as for the ground-state geometry optimization in the gas phase. Spin-orbit coupling matrix elements SOCME were calculated based on the optimized ground state geometry using PySOC.[7] The molecular orbital

distributions were visualized with Gaussview 6.0.[5] The RMSD of the $S_0$ and $S_1$ states were visualized using the VMD program.[8] Wavefunction calculations were performed using the Turbomole 7.5 package[9, 10] using the resolution of the identity approximation.[11] The spin-component scaling (SCS) modification was applied to all calculations.[12] The ground-state optimization was carried out using SCS-ADC(2)[13, 14] with the cc-pVDZ basis set,[15] starting from the DFT optimized ground state. Note that the ground-state optimized geometry from ADC(2) reduces to MP2.[16, 17] Vertical excited state calculations using SCS-ADC(2) with cc-pVDZ basis set were performed on the SCS-ADC(2) ground state optimized geometry with the first two singlet states[18] and first two triplet states[19, 20] calculated. Calculations were submitted and processed using the Digichem software package (version 6),[21, 22] which incorporates a number of publicly available software libraries, including: cclib[23] for parsing of result files, VMD[8]/Tachyon[24] for 3D rendering, Matplotlib for drawing of graphs[25], Open Babel[26]/Pybe[27] file interconversion.

***Photophysical measurements.*** Optically dilute solutions of concentrations on the order of $10^{-5}$ or $10^{-6}$ M were prepared in spectroscopic toluene for absorption and photoluminescence (PL) spectral analysis. Absorption spectra were recorded at room temperature on a Shimadzu UV-1800 double beam spectrophotometer with a 1 cm quartz cuvette. Molar absorptivity determination was verified by linear regression analysis of values obtained from five independent solutions at varying concentrations ranging from $5.3\times10^{-6}$ to $2.2\times10^{-5}$ M with absorbance ranging from 0.317 to 1.356.

For emission studies, steady-state PL, excitation spectra and time-resolved PL measurements were recorded at room temperature using an Edinburgh Instruments FS5 fluorimeter. Samples were excited at 340 nm for steady-state PL measurements and 375 nm for time-resolved PL decays. Aerated solution-state samples were prepared by bubbling solutions with compressed air for 5 minutes and spectra were measured using the cuvette for absorption analysis. Degassed solutions were prepared via three freeze-pump-thaw cycles and spectra were measured using a home-made Schlenk quartz cuvette. Photoluminescence quantum yields ($\Phi_{PL}$) for solutions were determined using the optically dilute method[28] in which four sample solutions with absorbances of ca. 0.092, 0.076, 0.057 and 0.046 at 360 nm were used. The Beer-Lambert law was found to remain linear across the concentrations of the solutions. For each sample, linearity between absorption and emission intensity was verified through linear regression analysis with the Pearson regression factor ($R^2$) for the linear fit of the data set surpassing 0.9. Individual

relative quantum yield values were calculated for each solution and the values reported represent the slope obtained from the linear fit of these results. The $\Phi_{PL}$ was determined using the equation $\Phi_{PL} = (\Phi_r * \frac{A_r}{A_s} * \frac{I_s}{I_r} * \frac{n_s^2}{n_r^2})$, where A stands for the absorbance at the excitation wavelength ($\lambda_{exc}$: 360 nm), I is the integrated area under the corrected emission curve and n is the refractive index of the solvent with the subscripts "s" and "r" representing sample and reference respectively. $\Phi_r$ is the absolute PL quantum yield of the external reference, quinine sulfate ($\Phi_r$ = 54.6% in 1 *N* $H_2SO_4$).[29]

For solid-state film measurements, the 1.5 wt% doped films of emitters in a host matrix, 98.5% w/w (9.85 mg) of host was dissolved in 0.9 mL of solvent and to this, 1.5% w/w (0.15 mg emitter in 100 μL of solvent) of emitter was added. Films with different concentration 1, 2, 5, 10 wt% were also prepared in the same way for the concentration screening. Thin films were then spin-coated onto a sapphire substrate using a spin speed of 1500 rpm for 60 s and annealed at 60 °C for 60 s. An integrating sphere (Edinburgh Instruments FS5, SC30 module) was employed for the photoluminescence quantum yield measurements of thin film samples. The $\Phi_{PL}$ of the films were then measured in air and in $N_2$ by purging the integrating sphere with $N_2$ gas flow for 2 min. The photophysical properties of the film samples were measured using an Edinburgh Instruments FS5 fluorimeter. Time-resolved PL measurements of the thin films were carried out using the time-correlated single-photon counting (TCSPC) technique. The samples were excited at 375 nm by a pulsed laser and were kept in a vacuum of $< 8 \times 10^{-4}$ mbar.

The singlet and triplet state energies in 2-MeTHF glass and in doped film were determined from the onset values of the steady-state PL (SS PL) and delayed emission spectra at 77 K. The singlet-triplet energy gap ($\Delta E_{ST}$) was estimated from the difference in energy of the onsets of these spectra. For SS PL, samples were excited by a xenon lamp emitting at 340 nm. For phosphorescence spectra, samples were excited by a xenon flashlamp emitting at 340 nm (EI FS5, SC-70). Phosphorescence spectra were measured with a time-gated window of 1-10 ms with xenon flashlamp operating at 100 Hz.

***Fitting of time-resolved photoluminescence measurements**:* Time-resolved PL measurements were fitted to a sum of exponentials decay model, with chi-squared ($\chi^2$) values between 1 and 2, using the Fluorcalc software. Each component of the decay is assigned a weight, ($w_i$), which is the contribution of the emission from each component to the total emission.

The average lifetime was then calculated using the following:

- Two exponential decay model:

$$\tau_{AVG} = \tau_1 w_1 + \tau_2 w_2$$

with weights defined as $w_1 = \frac{A1\tau_1}{A1\tau_1 + A2\tau_2}$ and $w_2 = \frac{A2\tau_2}{A1\tau_1 + A2\tau_2}$ where A1 and A2 are the preexponential-factors of each component.

- Three exponential decay model:

$$\tau_{AVG} = \tau_1 w_1 + \tau_2 w_2 + \tau_3 w_3$$

with weights defined as $w_1 = \frac{A1\tau_1}{A1\tau_1 + A2\tau_2 + A3\tau_3}$ , $w_2 = \frac{A2\tau_2}{A1\tau_1 + A2\tau_2 + A3\tau_3}$ and $w_3 = \frac{A3\tau_3}{A1\tau_1 + A2\tau_2 + A3\tau_3}$ where A1, A2 and A3 are the preexponential-factors of each component.

***Electrochemistry measurements.*** Cyclic Voltammetry (CV) analysis was performed on an Electrochemical Analyzer potentiostat model 620E from CH Instruments at a sweep rate of 100 mV/s. Differential pulse voltammetry (DPV) was conducted with an increment potential of 0.004 V and a pulse amplitude, width, and period of 50 mV, 0.05, and 0.5 s, respectively. Samples were prepared as DCM solutions, which were degassed by sparging with DCM-saturated argon gas for 5 minutes prior to measurements. All measurements were performed using 0.1 M DCM solution of tetra-*n*-butylammonium hexafluorophosphate ([$^n$Bu$_4$N]PF$_6$). An Ag/Ag$^+$ electrode was used as the reference electrode, while glassy carbon electrode and a platinum wire were used as the working electrode and counter electrode, respectively. The redox potentials are reported relative to a saturated calomel electrode (SCE) with a ferrocenium/ferrocene (Fc/Fc$^+$) redox couple as the internal standard (0.46 V vs SCE).[30] The redox potentials are reported relative to a saturated calomel electrode (SCE) with a ferrocene/ferrocenium (Fc/Fc$^+$) redox couple as the internal standard (0.46 V vs SCE). The HOMO and LUMO energies were determined using the relation $E_{HOMO}/_{LUMO} = -(E_{ox} / E_{red} + 4.8)$ eV, where $E_{ox}$ and $E_{red}$ are from DPV peak value relative to Fc/Fc$^+$.

***X-ray Crystallography.*** X-ray diffraction data for **DDOBDiKTa** were collected at 100 K using a Rigaku XtaLAB Synergy-S diffractometer equipped with a HyPix-Arc 100° Hybrid Photon Counting (HPC) detector, PhotonJet-S microfocus sealed-tube X-ray source [Mo Kα radiation

($\lambda$ = 0.71073 Å)], and MAX optics. Data were collected (using a calculated strategy) and processed (including correction for Lorentz, polarization and absorption) using CrysAlisPro.[31] The structure was solved by dual-space methods (SHELXT[32]) and refined by full-matrix least-squares against $F^2$ (SHELXL-2019/3[33]). Non-hydrogen atoms were refined anisotropically, and hydrogen atoms were refined using a riding model. The partial occupancy water solvate had its occupancy fixed at 0.5 after its occupancy refined close to 0.5. Two $^t$Bu groups on one independent molecule showed (rotational) positional disorder and were modelled in two parts with C-Me distances restrained. Two $^t$Bu-benzo groups at one end of one independent molecule showed continuous disorder with the fused benzo groups flexing out of plane. These were modelled in two parts with geometry of both $C_6$ rings and $^t$Bu groups restrained and thermal restraints on all parts. The structure showed a region of highly disordered solvent (993 Å$^3$) and the SQUEEZE[34] routine implemented in PLATON[35] was used to remove the contribution to the diffraction pattern of the unordered electron density in the void spaces. All calculations except SQUEEZE were performed using the Olex2[36] interface. Selected crystallographic data: $C_{60}H_{53.33}B_2NO_{6.17}$, $M$ = 908.65, triclinic, $a$ = 16.8370(3), $b$ = 21.7387(4), $c$ = 22.6741(4) Å, $\alpha$ = 77.0374(15), $\beta$ = 76.1074(17), $\gamma$ = 88.0263(14) °, $U$ = 7849.4(2) Å$^3$, $T$ = 100 K, space group $P\bar{1}$ (no. 2), $Z$ = 6, 144152 reflections measured, 30725 unique ($R_{int}$ = 0.0591), which were used in all calculations. The final $R_1$ [$I > 2\sigma(I)$] was 0.0672 and $wR_2$ (all data) was 0.2118.

**OLED Fabrication and Characterization**

Bottom-emitting OLEDs were fabricated using pre-patterned indium tin oxide (ITO) on a glass substrate. The substrate was cleaned with acetone then isopropanol for 30 min each, using an ultrasonication bath set at 60 °C. The OLEDs were fabricated using a thermal evaporator (Angstrom Engineering, USA) under a high vacuum with a base pressure of around $3\times10^{-7}$ mbar. The substrate was loaded into the thermal evaporator using a custom-made air-tight metal container to avoid dust particles and contamination. All organic layers were deposited on the ITO anode with evaporation rates between 0.3-0.6 Å/s. This was followed by lithium fluoride (LiF) and aluminium (Al) deposition at the rates of 0.05 Å/s and 2 Å/s, respectively. The fabricated OLEDs were encapsulated in $N_2$ atmosphere using glass that had been cleaned in the same way as the substrate. A UV-curable epoxy resin (NOA 68) was used to ensure an air-tight attachment of the encapsulation with the substrate. The active (emission) area of the OLEDs was 2 mm$^2$. The fabricated OLEDs were measured for their current-voltage-luminance characteristics using a Keithley 2400 power source meter and a custom-made photodiode system connected to a

Keithley 2000 multimeter. The electroluminescence spectra of the devices were analyzed using a CCD (Andor DV420-BV) spectrometer.

## 2. Synthesis section

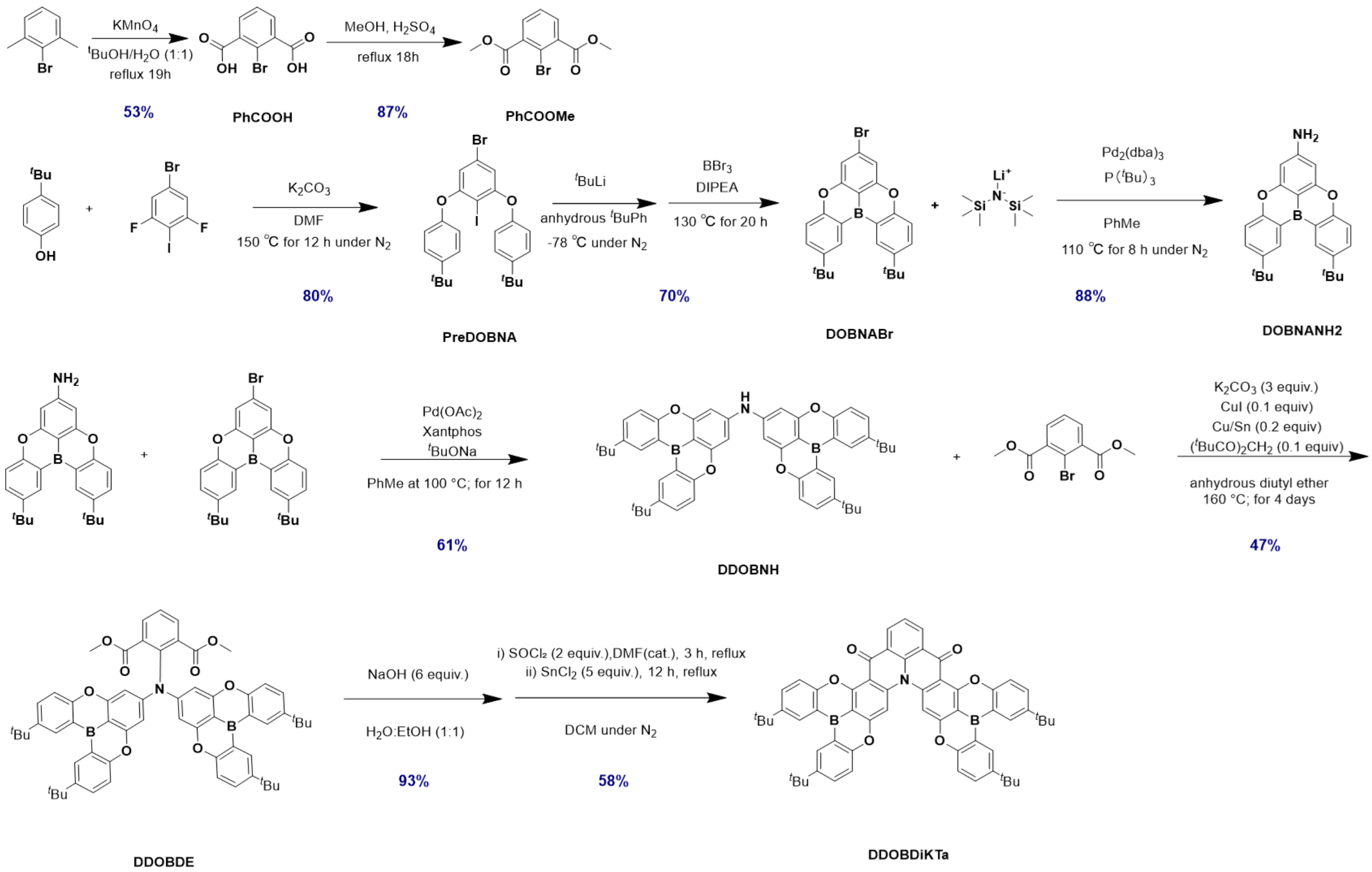


Figure **S1**. Synthetic route of **DDOBDiKTa**

### 2-bromoisophthalic acid (PhCOOH)

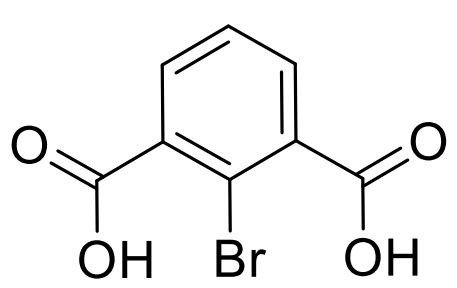


To a 100 mL mixture of 1:1 $^t$BuOH: $H_2O$ was added **2-bromo-m-xylene** (9.40 g, 50.79 mmol, 1.0 equiv.). $KMnO_4$ (33.71 g, 213.3 mmol, 4.2 equiv.) and 17 g of Celite were then added and the mixture was stirred at reflux for 19 h. After cooling to RT, the reaction mixture was filtered over Celite and concentrated to 1/3 volume under reduced pressure. 20 mL of concentrated HCl was then carefully added until the product precipitated as a white solid. The solids were filtered and dried overnight in a vacuum desiccator to obtain a white powder. **Yield** 53%. **Mp**: 222-223 °C (lit. Mp:[37] 223-224 °C). **R*f***: 0.42 (EA: hexane = 2:1). **$^1$H NMR** (500 MHz, methanol-$d_4$) δ 7.75 (d, $J$ = 7.6

Hz, 2H), 7.52 (t, $J = 7.7$ Hz, 1H). **$^{13}$C NMR** (126 MHz, methanol-$d_4$) δ 168.54, 136.55, 131.14, 127.13, 117.03.

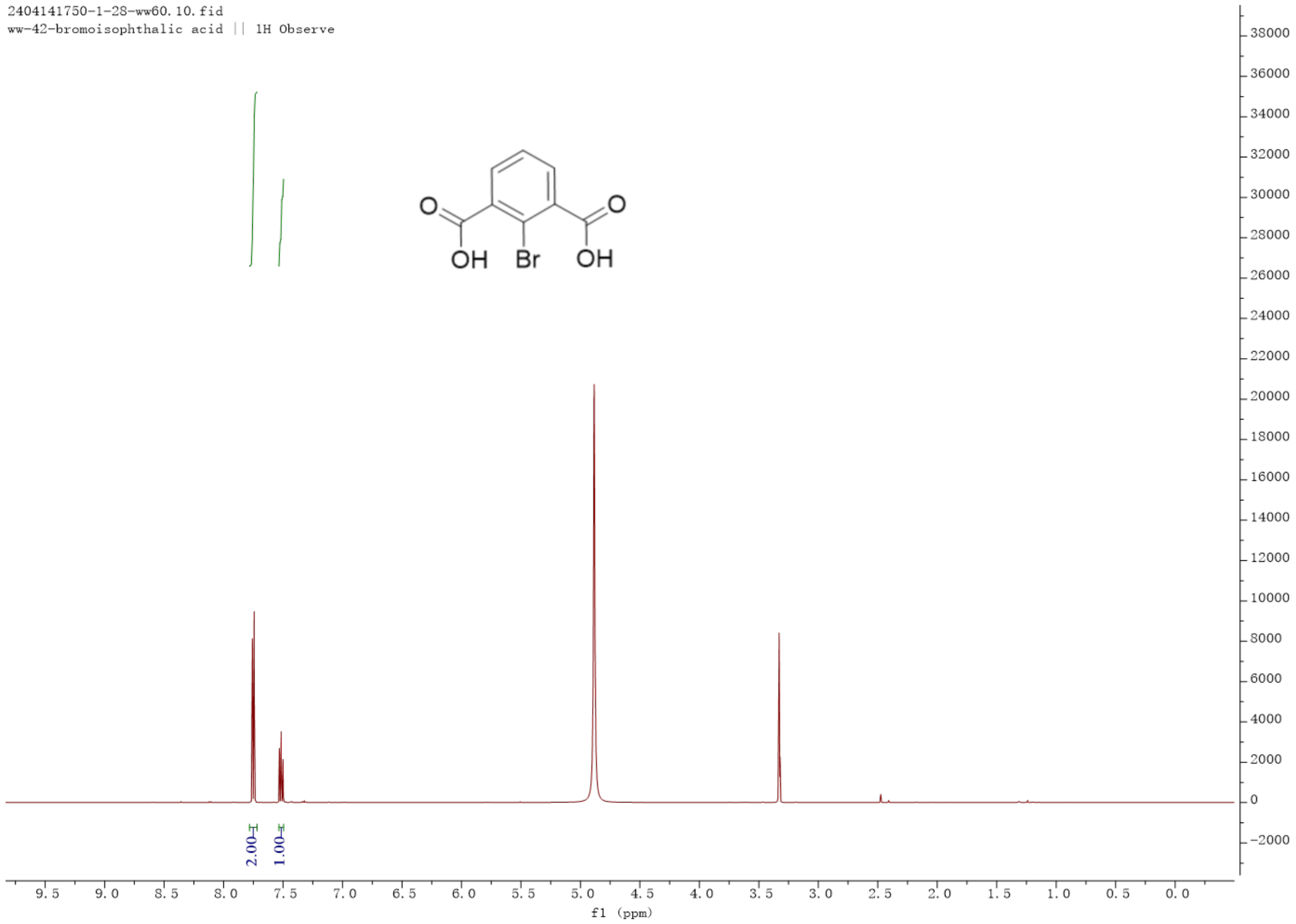


Figure **S2**. $^1$H NMR spectrum of **2-bromoisophthalic acid** in methanol-$d_4$

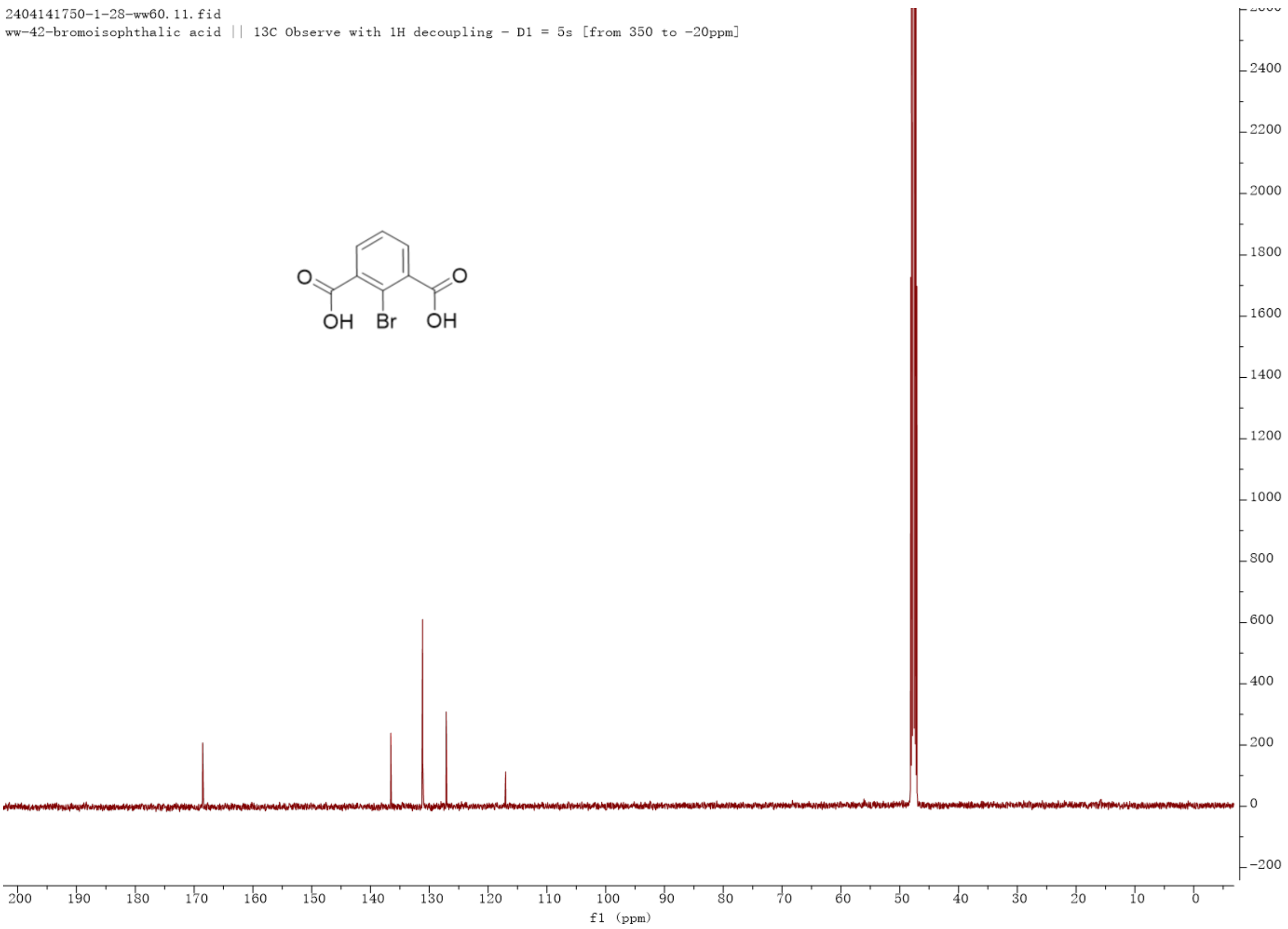


Figure **S3**. ${}^{13}$C NMR spectrum of **2-bromoisophthalic acid** in methanol-$d_4$

**Dimethyl 2-bromoisophthalate (PhCOOMe)**

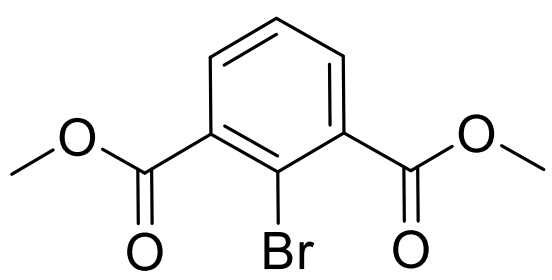


To a flask containing 30 mL of methanol was added **2-bromoisophthalic acid** (3.00 g, 12.24 mmol, 1.0 equiv.). 1 mL of concentrated $H_2SO_4$ was then carefully added and the reaction mixture was stirred at reflux for 16 h. After cooling to RT, the reaction was diluted with 25 mL of $H_2O$ and extracted with 2× 100 mL of DCM. The organic phase was washed with 30 mL of aqueous solution of sat. $NaHCO_3$ and 30 mL of brine. The organics were dried over anhydrous $Na_2SO_4$, filtered, and the solvent evaporated under reduced pressure to afford a yellow oily product in a yield of 87%. **R*f***: 0.35 (EA: hexane = 1:4). **${}^{1}$H NMR** (500 MHz, chloroform-*d*) δ 7.71 (d, $J$ = 7.7 Hz, 2H), 7.42 (t, $J$ = 7.7 Hz, 1H), 3.95 (s, 6H). **${}^{13}$C NMR** (126 MHz, chloroform-*d*) δ 166.95, 135.43, 132.28, 127.11, 119.13, 52.78. **GC-MS** (retention time: 6.3 min): Calculated: 271.97 ($C_{10}H_9BrO_4$); Found: 272.

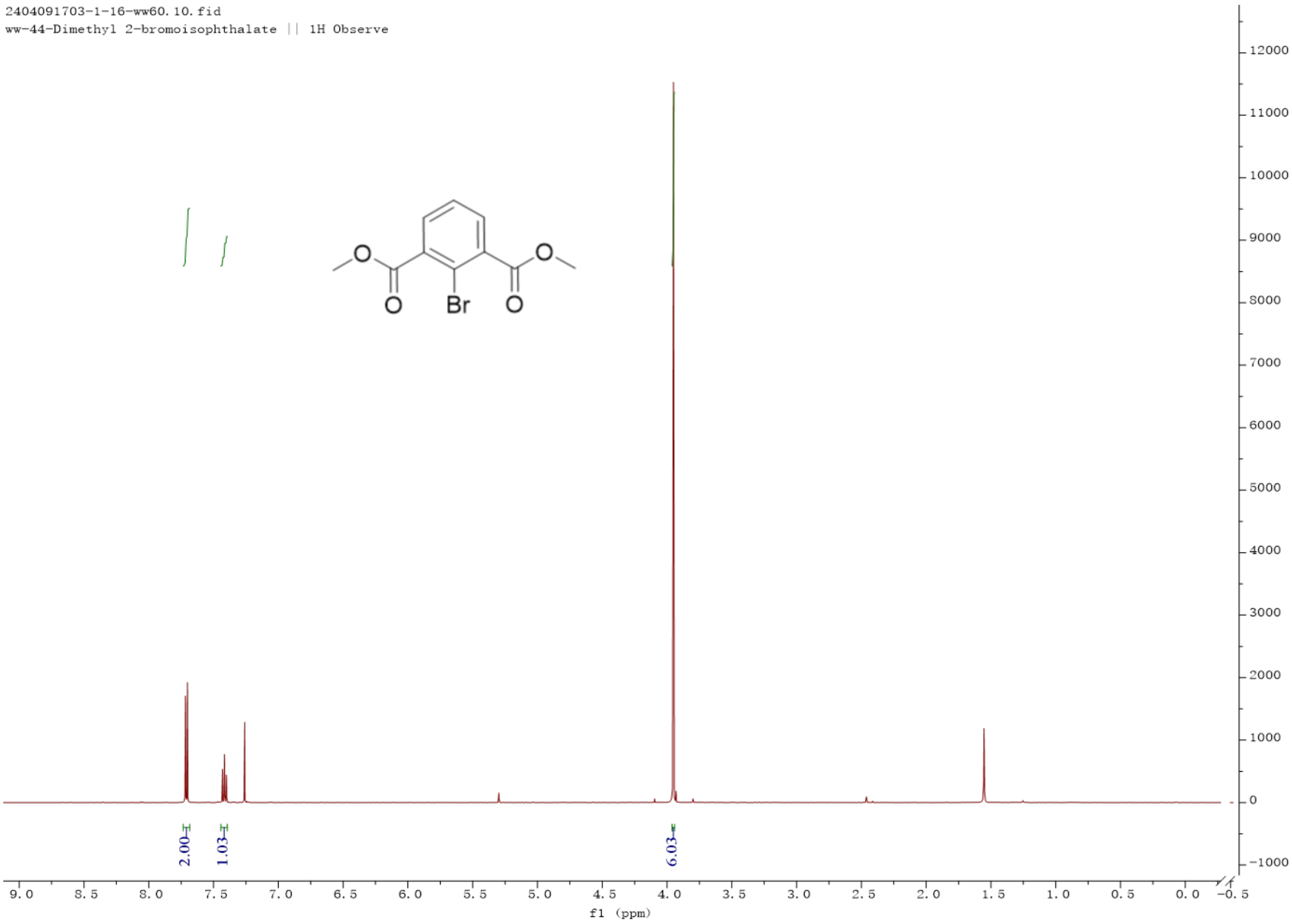


Figure **S4**. $^{1}$H NMR spectrum of **Dimethyl 2-bromoisophthalate** in $CDCl_3$

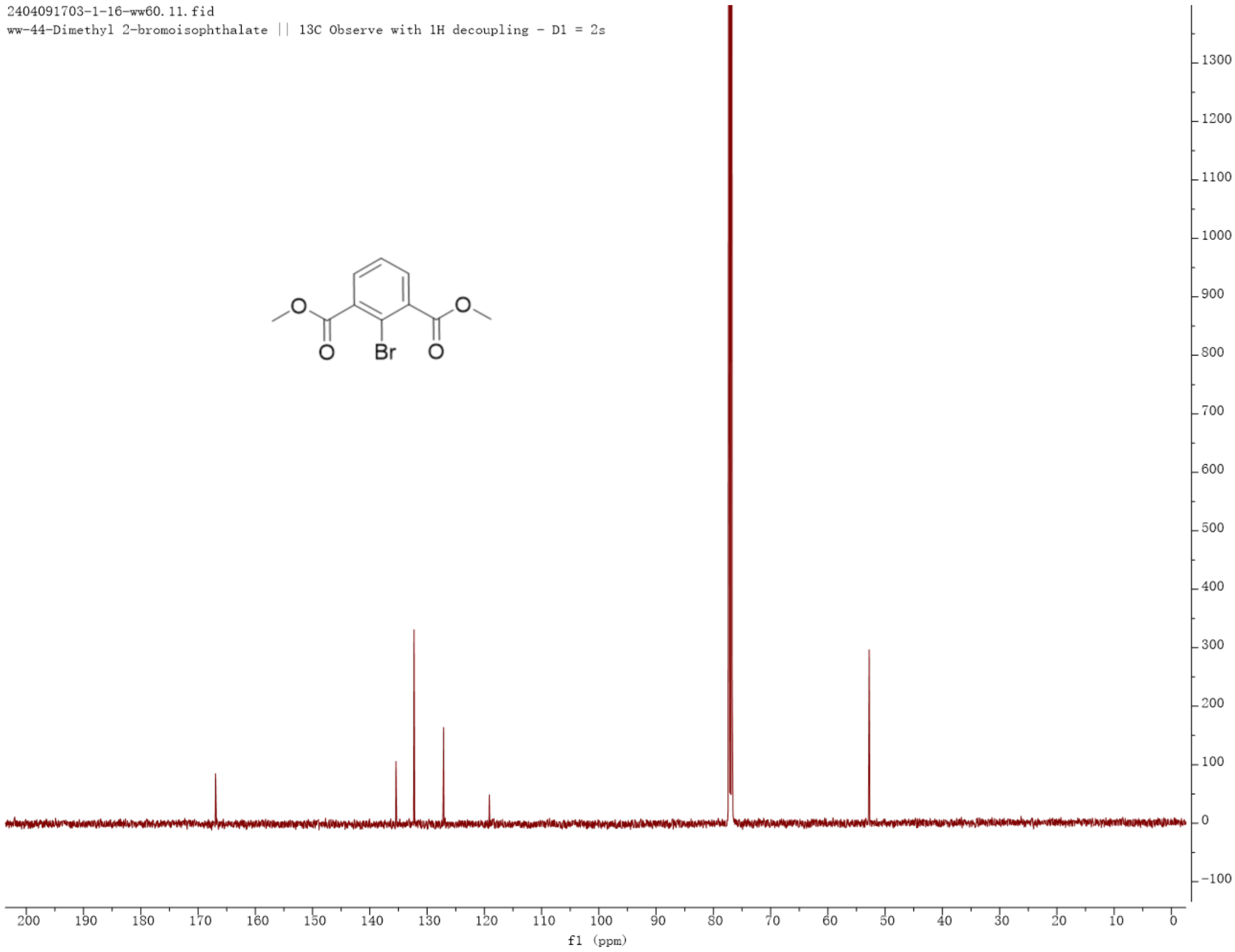


Figure **S5**. $^{13}$C NMR spectrum of **Dimethyl 2-bromoisophthalate** in $CDCl_3$

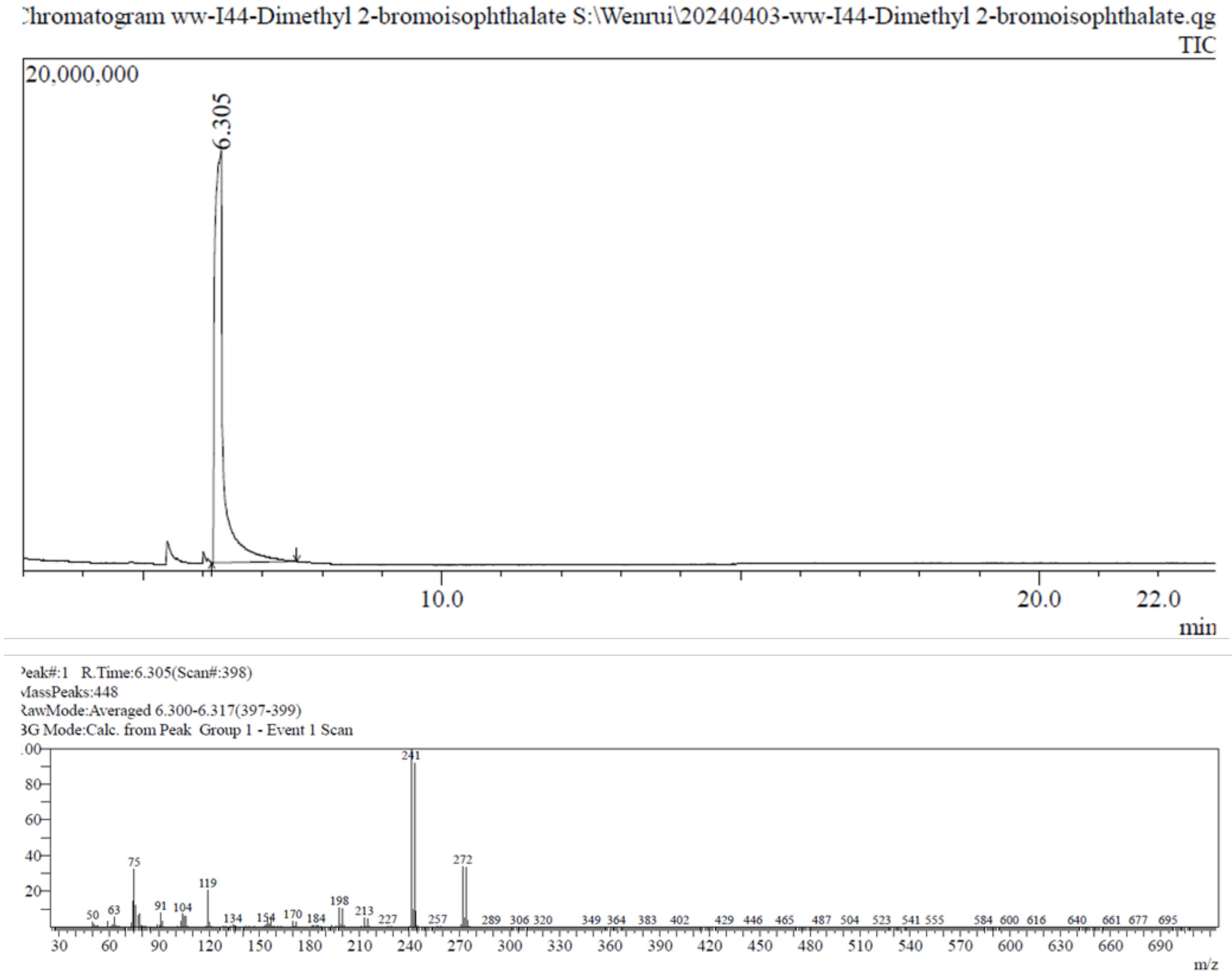


Figure **S6**. GC-MS spectrum of **Dimethyl 2-bromoisophthalate**

**4,4'-((5-bromo-2-iodo-1,3-phenylene)bis(oxy))bis(*tert*-butylbenzene) (PreDOBNA)**

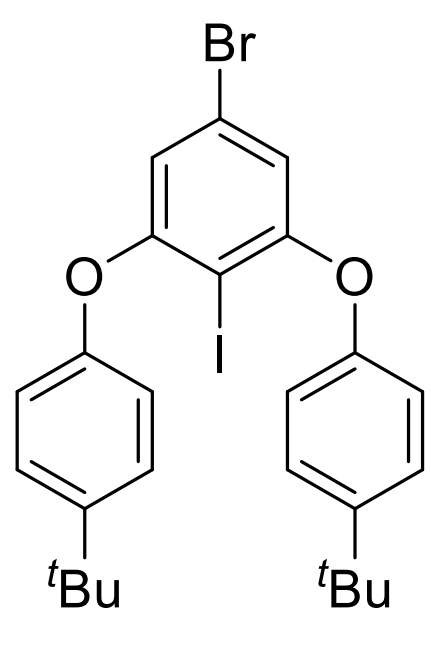


4-*tert*-butylphenol (22.71 g, 151.2 mmol, 1.0 equiv.), 5-bromo-1,3-difluoro-2-iodobenzene (16.07 g, 50.4 mmol, 0.33 equiv.), $K_2CO_3$ (20.90 g, 151.2 mmol, 1.0 equiv.) and 140 mL of DMF were added to a 250 mL Schlenk tube under nitrogen. The reaction was heated to 150 °C and stirred for 12 h. After cooling down, the solvent and all volatiles were removed under reduced pressure. The residue was washed with 100 mL of $CH_3OH$ and 100 mL of water to afford 23.0 g of white powder product in a yield of 80%. **Mp**: 158-159 ℃. **R*f***: 0.35 (DCM: hexane = 1:9). **$^1$H NMR** (400 MHz, dichloromethane-*d*$_2$) δ 7.50–7.43 (m, 4H), 7.05–6.99 (m, 4H), 6.71 (s, 2H), 1.38 (s, 18H). **$^{13}$C NMR** (126 MHz, chloroform-*d*) δ 159.51, 153.40, 147.53, 126.93, 122.81, 118.99, 115.24, 34.47, 31.49. **GC-MS** (retention time: 13.8 min): Calculated: 579.32 ($C_{26}H_{28}BrIO_2$); Found: 580.

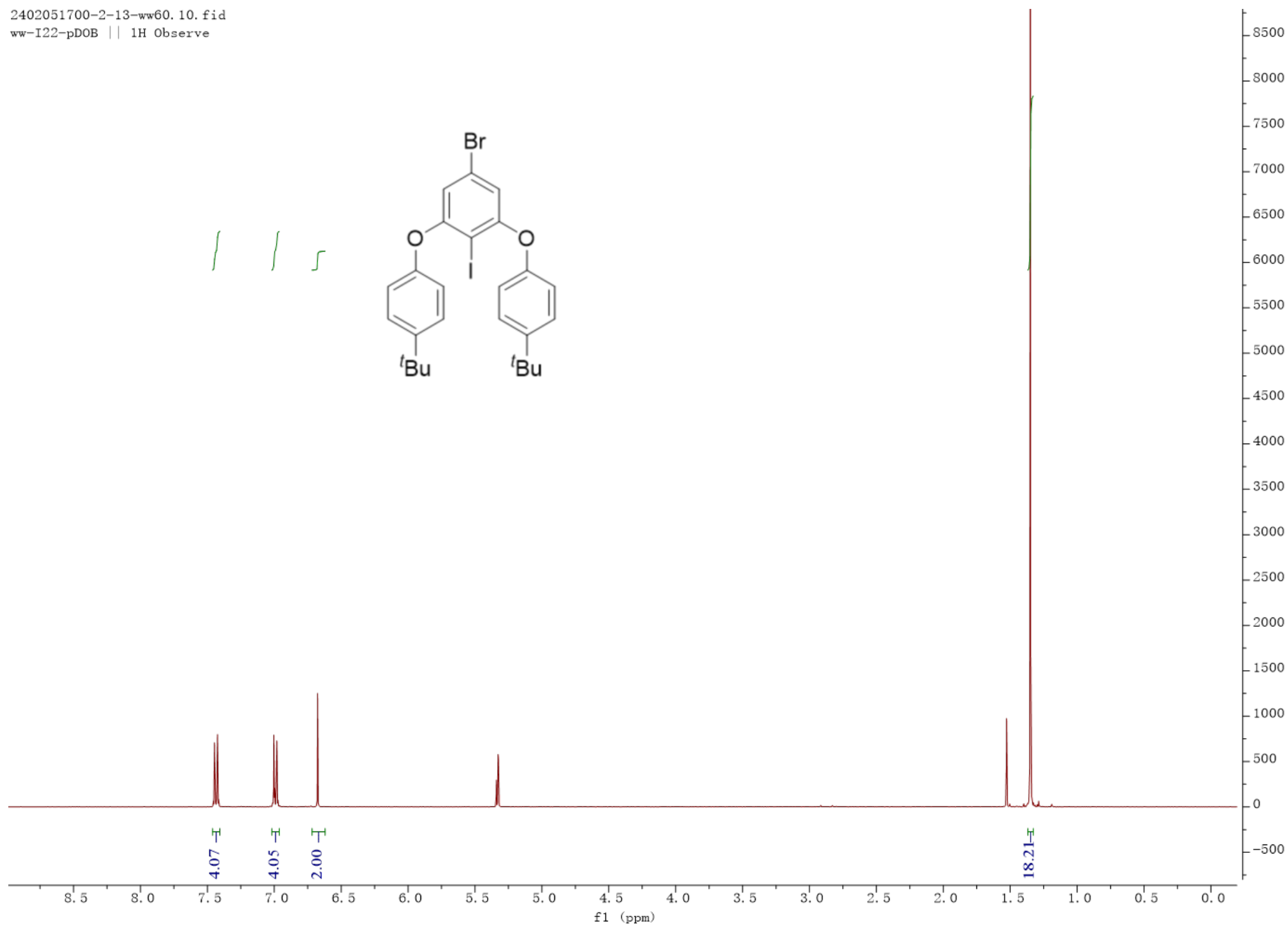


Figure **S7**. $^{1}H$ NMR spectrum of **PreDOBNA** in $CD_2Cl_2$

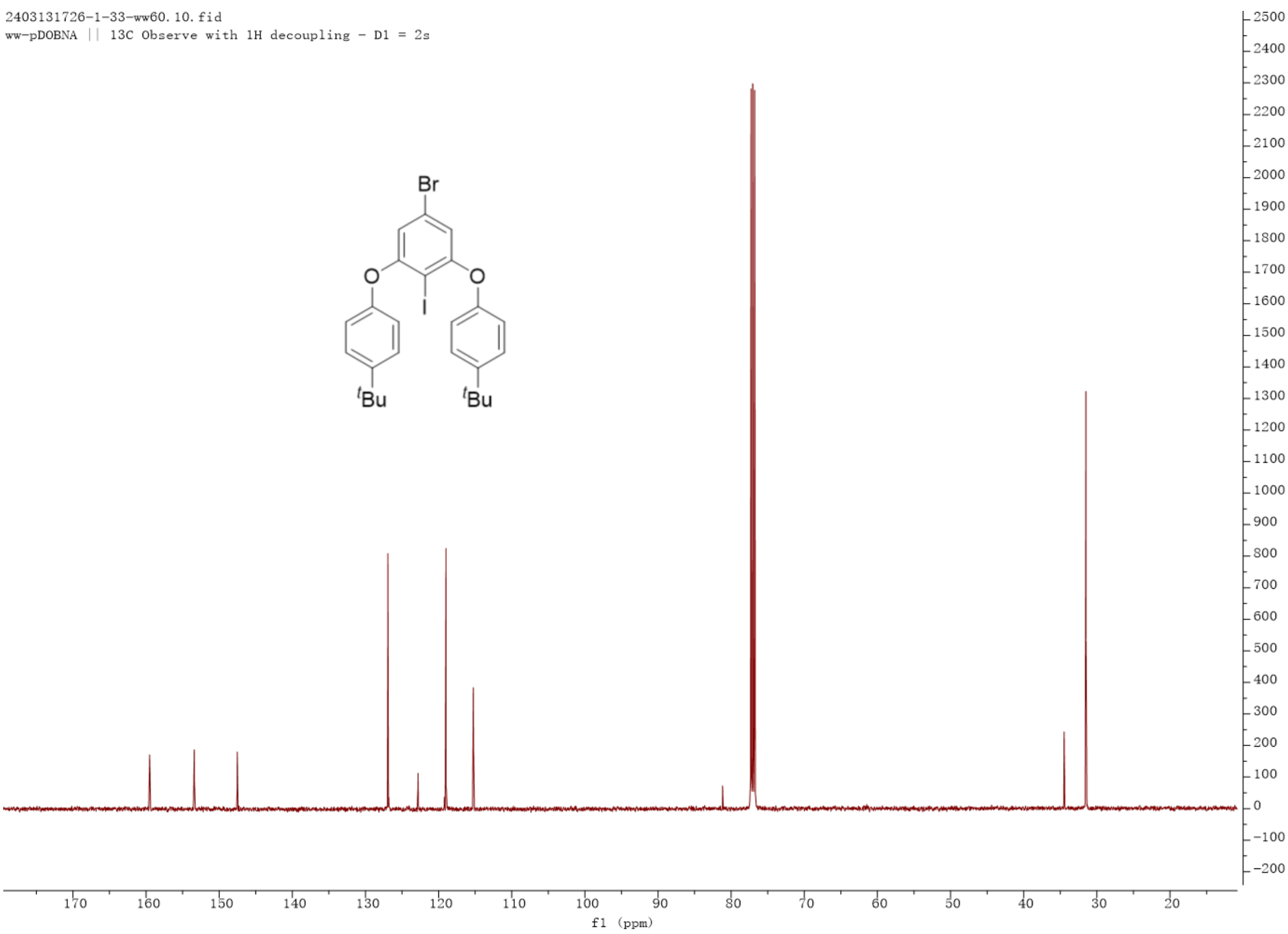


Figure **S8**. $^{13}C$ NMR spectrum of **PreDOBNA** in $CDCl_3$

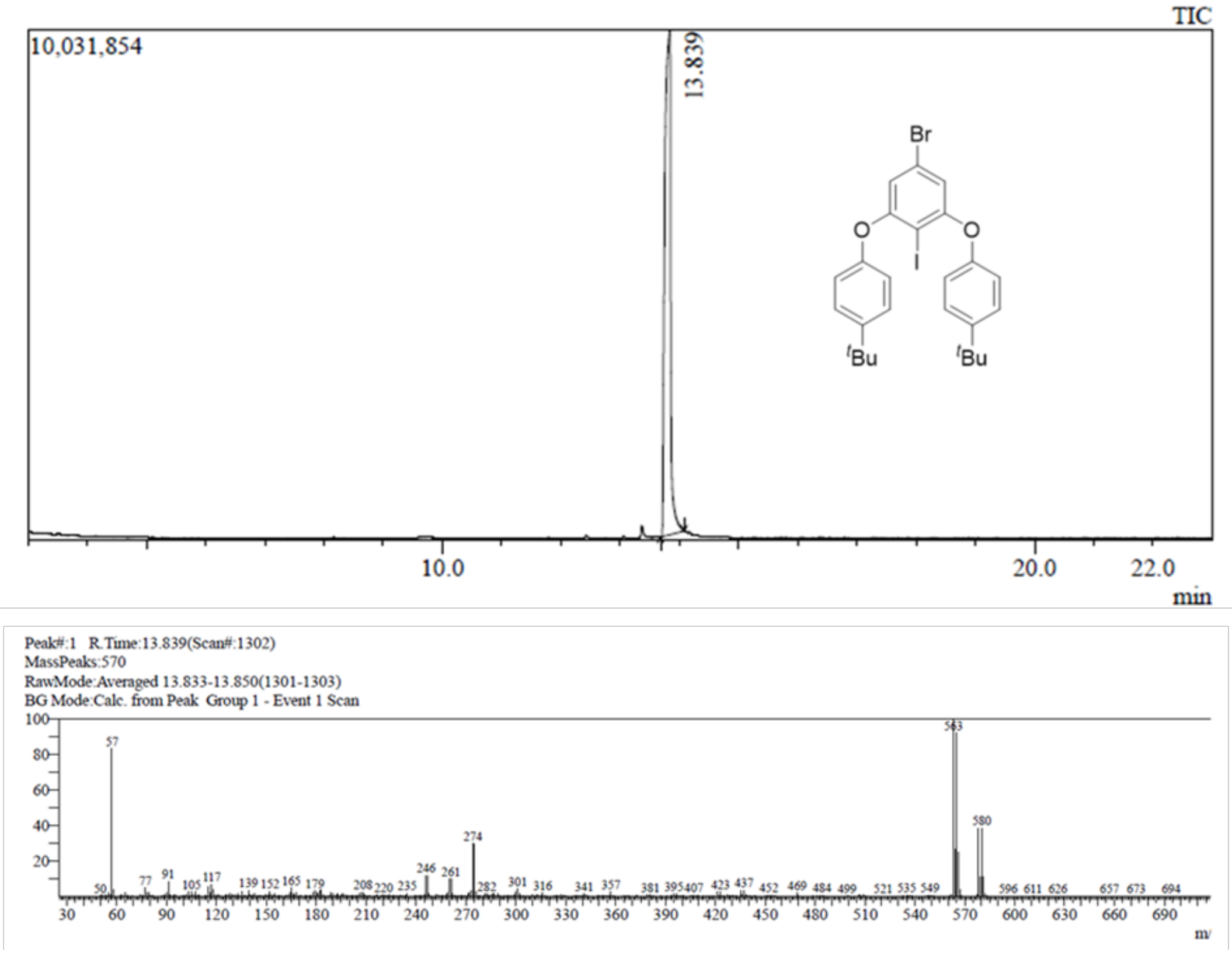


Figure **S9**. GC-MS spectrum of **PreDOBNA**

**7-bromo-2,12-di-*tert*-butyl-5,9-dioxa-13b-boranaphtho[3,2,1-de]anthracene (DOBNABr)**

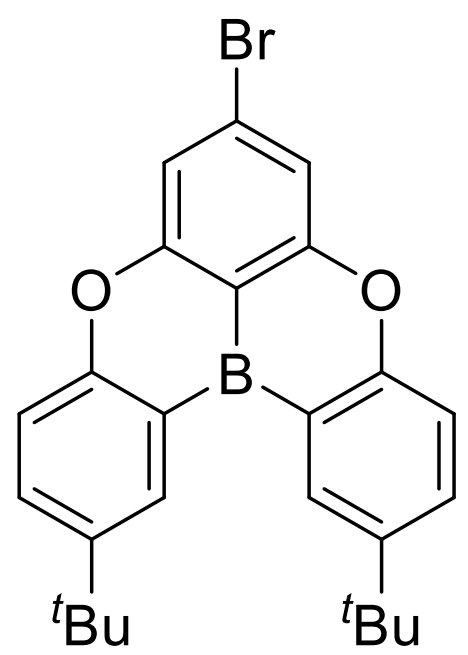


A solution of *tert*-butyllithium in pentane (3.24 mL, 1.7 M, 5.5 mmol, 1.1 equiv.) was slowly added to a solution of **PreDOBNA** (2.90 g, 5.00 mmol, 1.0 equiv.) in anhydrous *tert*-butylbenzene (20 mL) at -78 °C under a nitrogen atmosphere. After stirring the reaction at -78 °C for 1 h, the mixture was allowed to warm to room temperature and stirred for 2 h. The solution was again cooled to -78 °C, and boron tribromide (0.57 mL, 6.00 mmol, 1.2 equiv.) was then added slowly with stirring. After 1 h, the mixture was allowed to warm to room temperature and stirred for 30 min, before heating to 45 °C for 1 h. The reaction was cooled to 0 °C and *N,N*-diisopropylethylamine (1.29 g, 10.00 mmol, 2.0 equiv.) was added. The reaction mixture was allowed to warm to room temperature and stirred for 30 min, then heated to 135 °C for 20 h. Upon cooling to room temperature, the reaction was quenched with 20 mL of methanol. The reaction was concentrated under reduced pressure. The crude product was purified by flash column chromatography (eluent: petroleum ether) to obtain 1.6 g of white powder product with yield of 70%. **Mp:** 218-220 °C. **R*f*:** 0.65 (DCM: hexane = 1:9). $^{1}$**H NMR** (400 MHz, dichloromethane-$d_2$) δ 8.79 (d, *J* = 2.5 Hz, 2H), 7.86 (dd, *J* = 8.8, 2.6 Hz, 2H), 7.53

(d, $J$ = 8.8 Hz, 2H), 7.42 (s, 2H), 1.48 (s, 18H). **$^{13}$C NMR** (126 MHz, chloroform-$d$) δ 158.43, 157.78, 145.43, 131.75, 130.23, 128.02, 118.03, 111.97, 34.59, 31.54. **GC-MS** (retention time: 14.6 min): Calculated: 460.12 ($C_{26}H_{26}BBrO_2$); Found: 460.

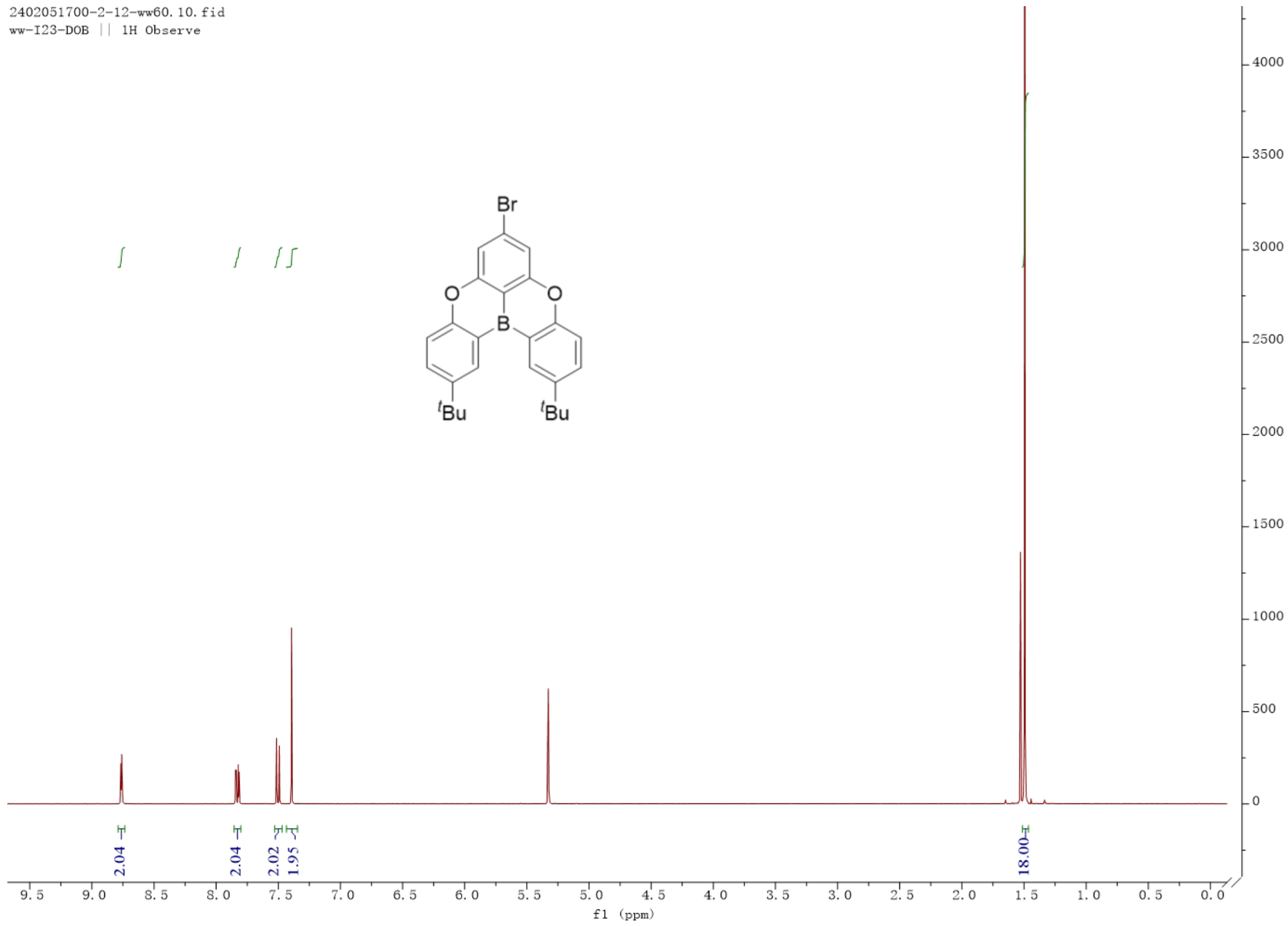


Figure **S10**. $^1$H NMR spectrum of **DOBNABr** in $CD_2Cl_2$

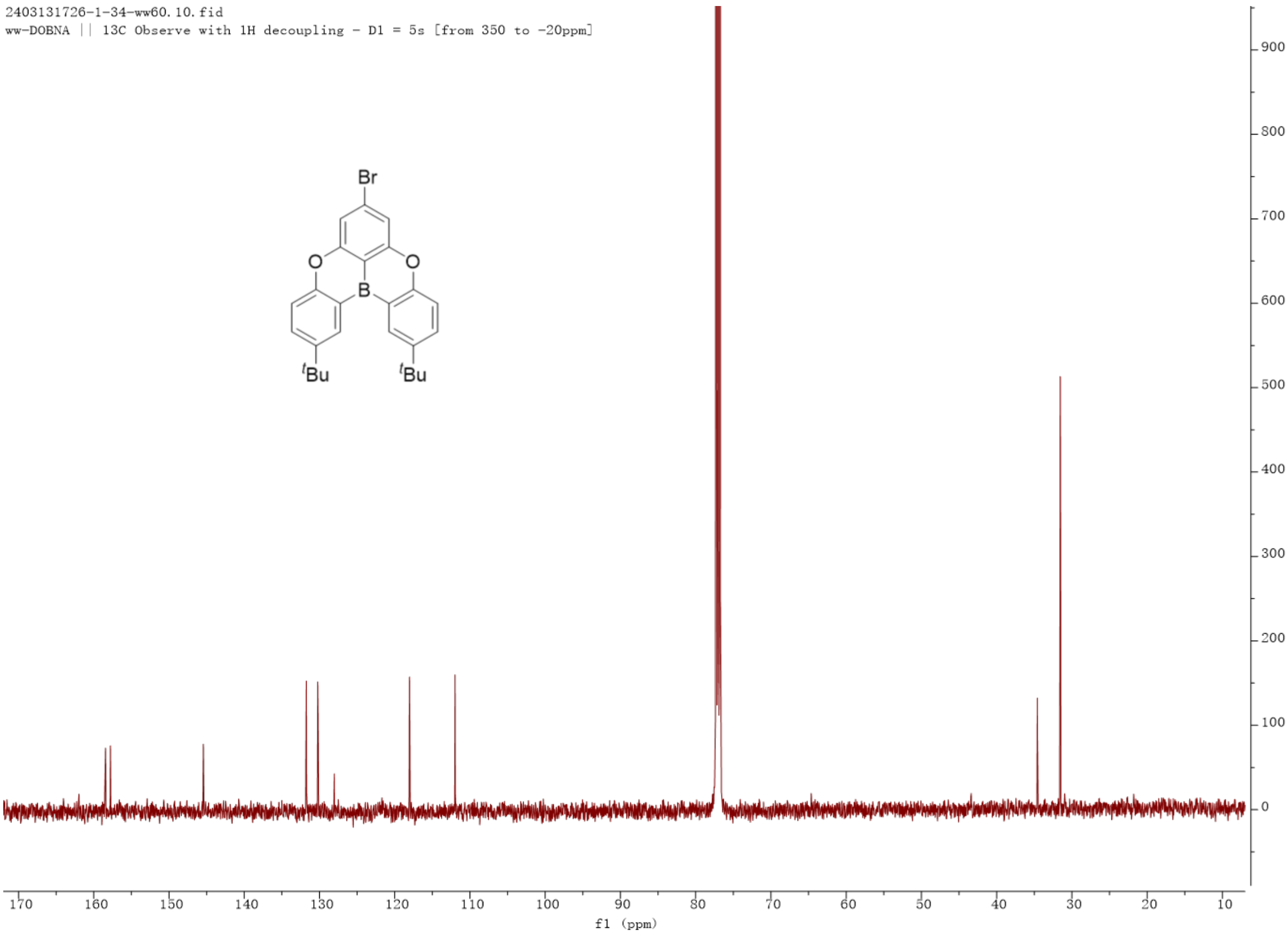


Figure **S11**. $^{13}$C NMR spectrum of **DOBNABr** in $CDCl_3$

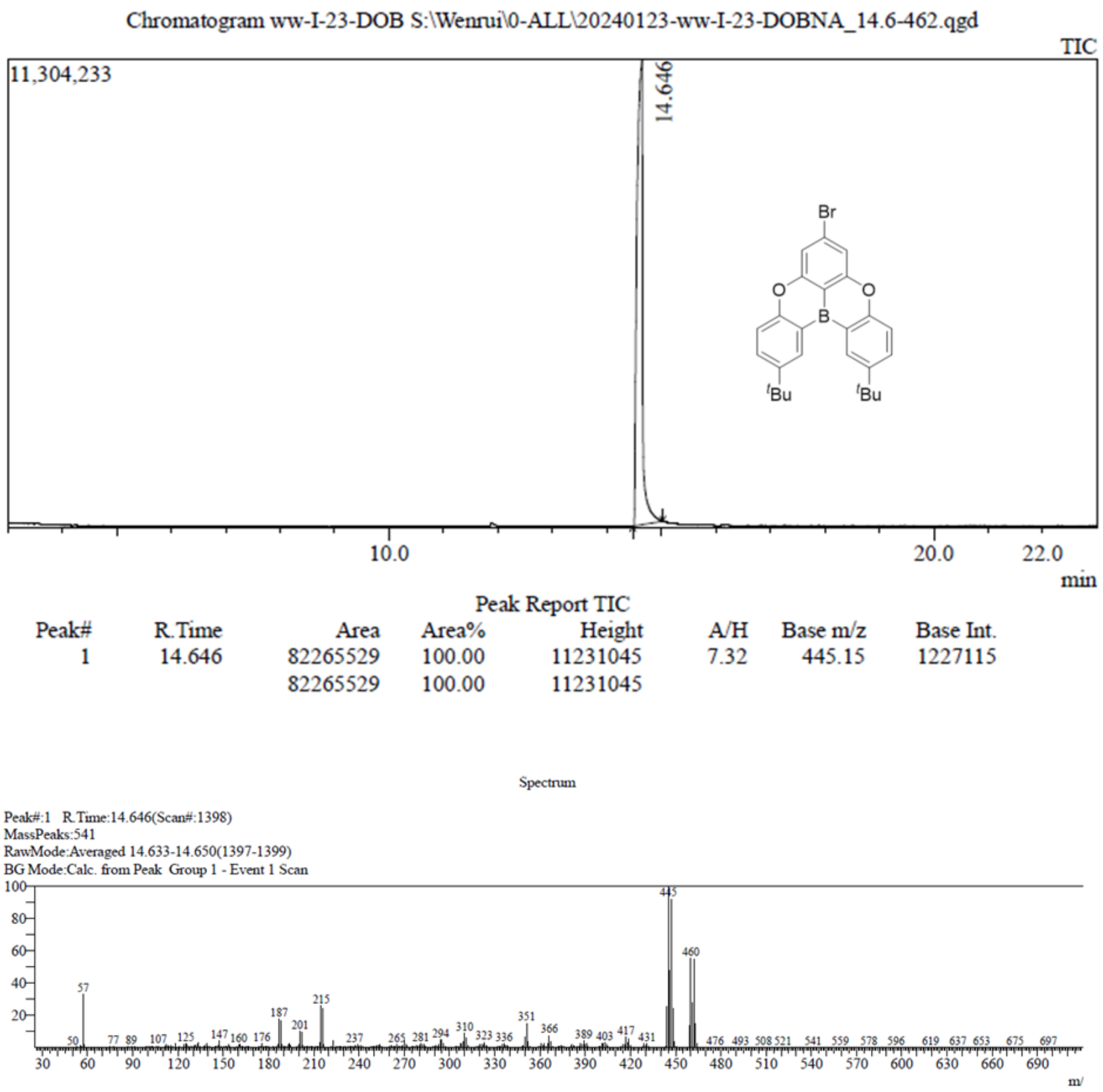


Figure **S12**. GC-MS spectrum of **DOBNABr**

**2,12-di-*tert*-butyl-5,9-dioxa-13b-boranaphtho[3,2,1-de]anthracen-7-amine(DOB-NANH2)**

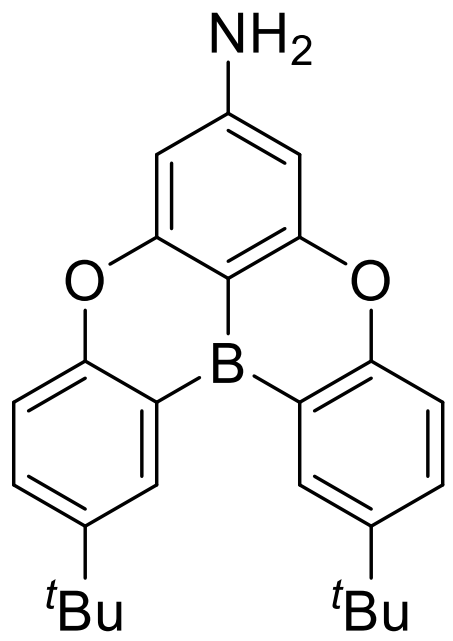


**DOBNABr** (2.00 g, 4.336 mmol, 1.0 equiv.), lithium bis(trimethylsilyl)amide (1.09 g, 6.505 mmol, 1.5 equiv.), tris(dibenzylideneacetone)dipalladium (0) (99.3 mg, 0.1084 mmol, 0.025 equiv.), tri-*tert*-butylphosphane (21.9 mg, 0.1084 mmol, 0.025 equiv.) and 30 mL toluene were added to a 100 mL Schlenk tube under nitrogen. The reaction was heated at 110 °C for 8 h. After cooling to room temperature, the mixture was extracted with 50 mL of brine and 50 mL of DCM. The crude mixture was purified by silica gel flash column chromatography (eluent: ethyl acetate: hexane = 1:4) to afford the desired 1.32 g product as yellow powder in a yield of 88%. **Mp**: 211-212 °C. **$R_f$:** 0.11 (EA: hexane = 1:4). **$^1$H NMR** (500 MHz, chloroform-*d*) δ 8.69 (d, *J* = 2.5 Hz, 2H), 7.70 (dd, *J* = 8.7, 2.5 Hz, 2H), 7.41 (d, *J* = 8.7 Hz, 2H), 6.46 (s, 2H), 4.22 (s, 2H), 1.48 (s, 18H). **$^{13}$C NMR** (126 MHz, chloroform-*d*) δ 159.27, 158.53, 152.93, 144.68, 130.49, 130.10, 117.61, 94.67, 34.51, 31.60. **GC-MS** (retention time: 15.1 min): Calculated: 397.22 ($C_{26}H_{28}BNO_2$); Found: 397.

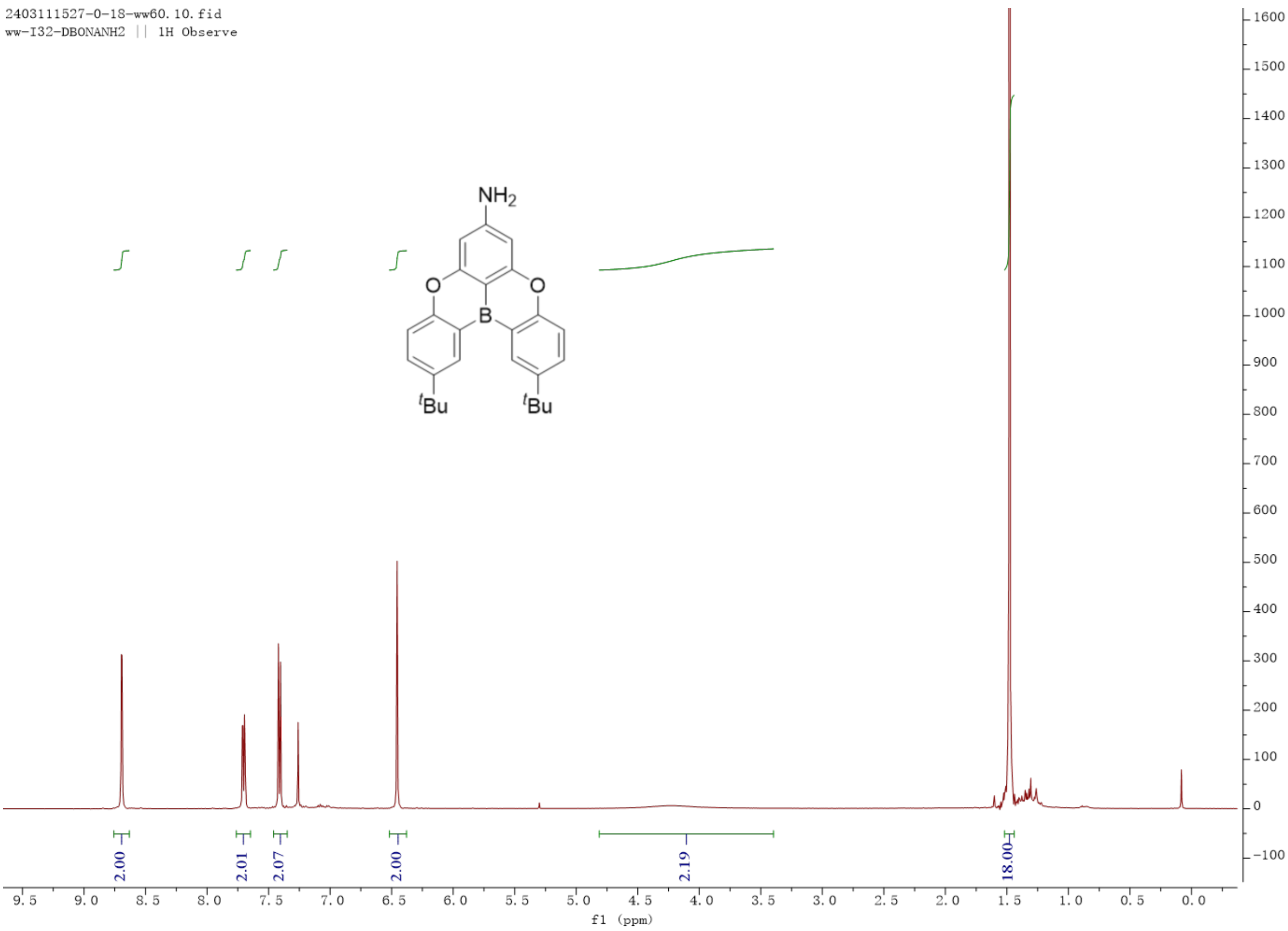


Figure **S13**. $^1$H NMR spectrum of **DOBNANH2** in $CDCl_3$

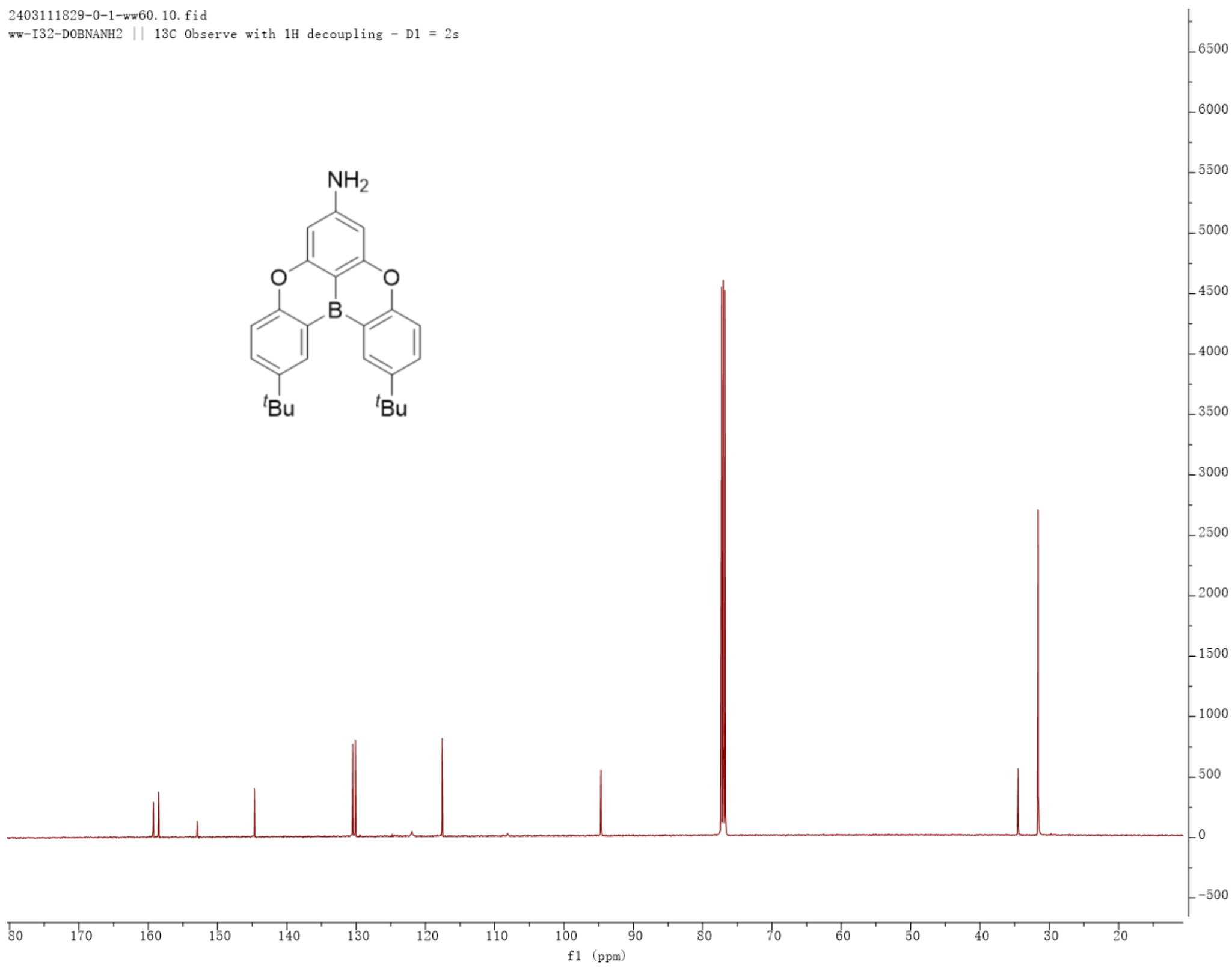


Figure **S14**. $^{13}$C NMR spectrum of **DOBNANH2** in $CDCl_3$

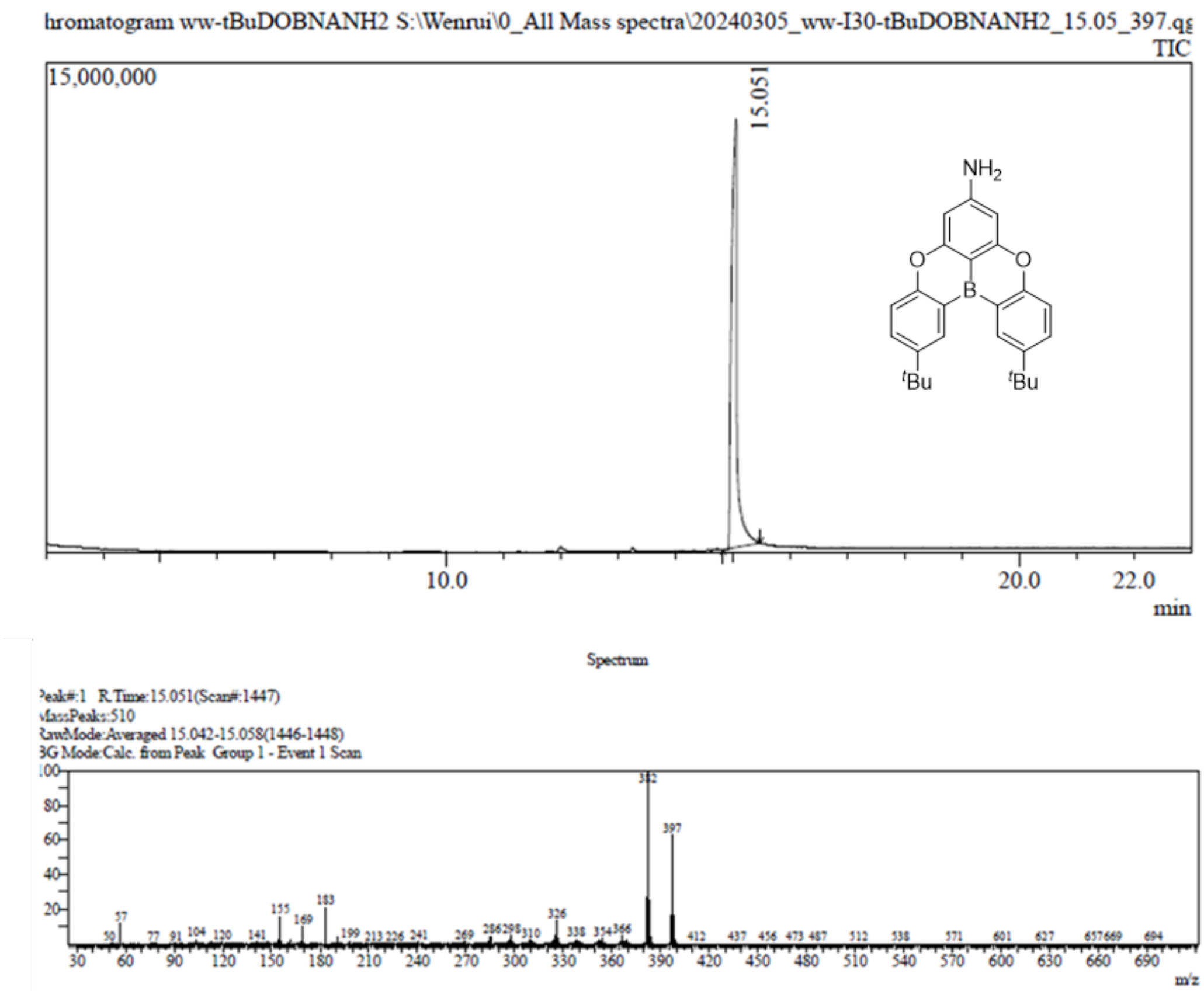


Figure **S15**. GC-MS spectrum of **DOBNANH2**

**bis(2,12-di-*tert*-butyl-5,9-dioxa-13b-boranaphtho[3,2,1-de]anthracen-7-yl)amine (DDOBNH)**

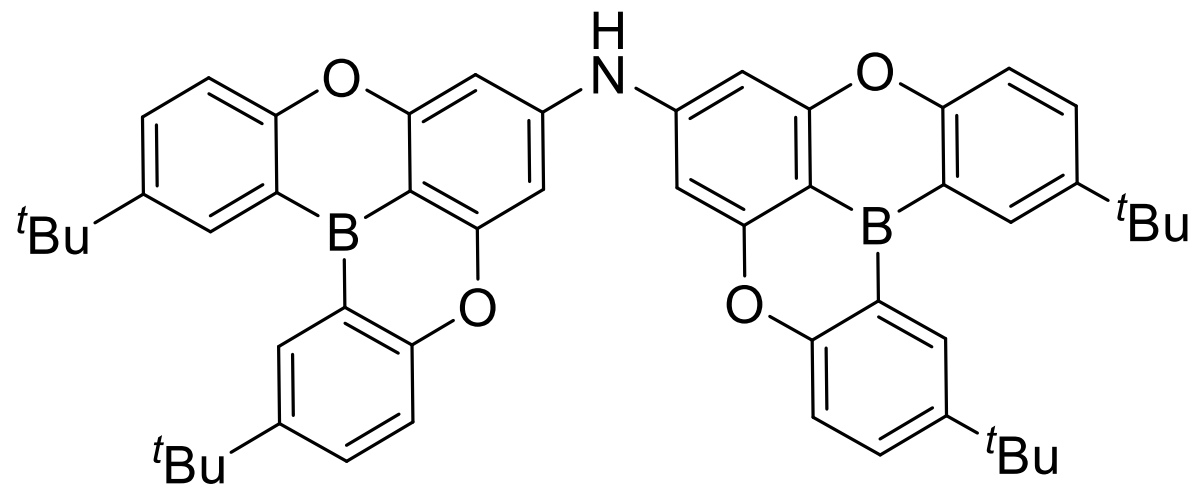

**DOBNABr** (2.15 g, 4.662 mmol, 1.0 equiv.), **DOBNANH2** (1.85 g, 4.662 mmol, 1.0 equiv.), palladium acetate (52.3 mg, 233.1 μmol, 0.05 equiv.), sodium *tert*-butoxide (1.12 g, 11.65 mmol, 2.5 equiv.), xantphos (269.7 mg, 466.2 μmol, 0.1 equiv.) and 65 mL toluene were added to a 250 mL Schlenk tube under nitrogen. The reaction was heated at 120 °C for 24 h. After cooling to room temperature, the mixture was extracted 3× with 200 mL each of brine and DCM. The organic phase was dried over anhydrous $Na_2SO_4$, filtered, then half the solvent was evaporated under reduced pressure. Methanol (50 mL) was then added to obtain an off-white precipitate, which was filtered to afford 2.20 g of off-white product in a yield of 61%. **Mp**: 383-385 °C. **R*f***: 0.46 (EA: hexane = 1:4). **$^1$H NMR** (500 MHz, dichloromethane-*d*$_2$) δ 8.79 (d, *J* = 2.5 Hz, 4H), 7.82 (dd, *J* = 8.7, 2.5 Hz, 4H), 7.52 (d, *J* = 8.7 Hz, 4H), 7.14 (s, 4H), 6.83 (s, 1H), 1.53 (s, 36H). **$^{13}$C NMR** (126 MHz, dichloromethane-*d*$_2$) δ159.04, 158.64, 147.90, 145.31, 131.17, 130.18, 117.79, 97.76, 34.41, 31.27. **HRMS [M$^+$]**: Calculated: 778.4161 ($C_{52}H_{53}B_2NO_4$); Found 778.4237.

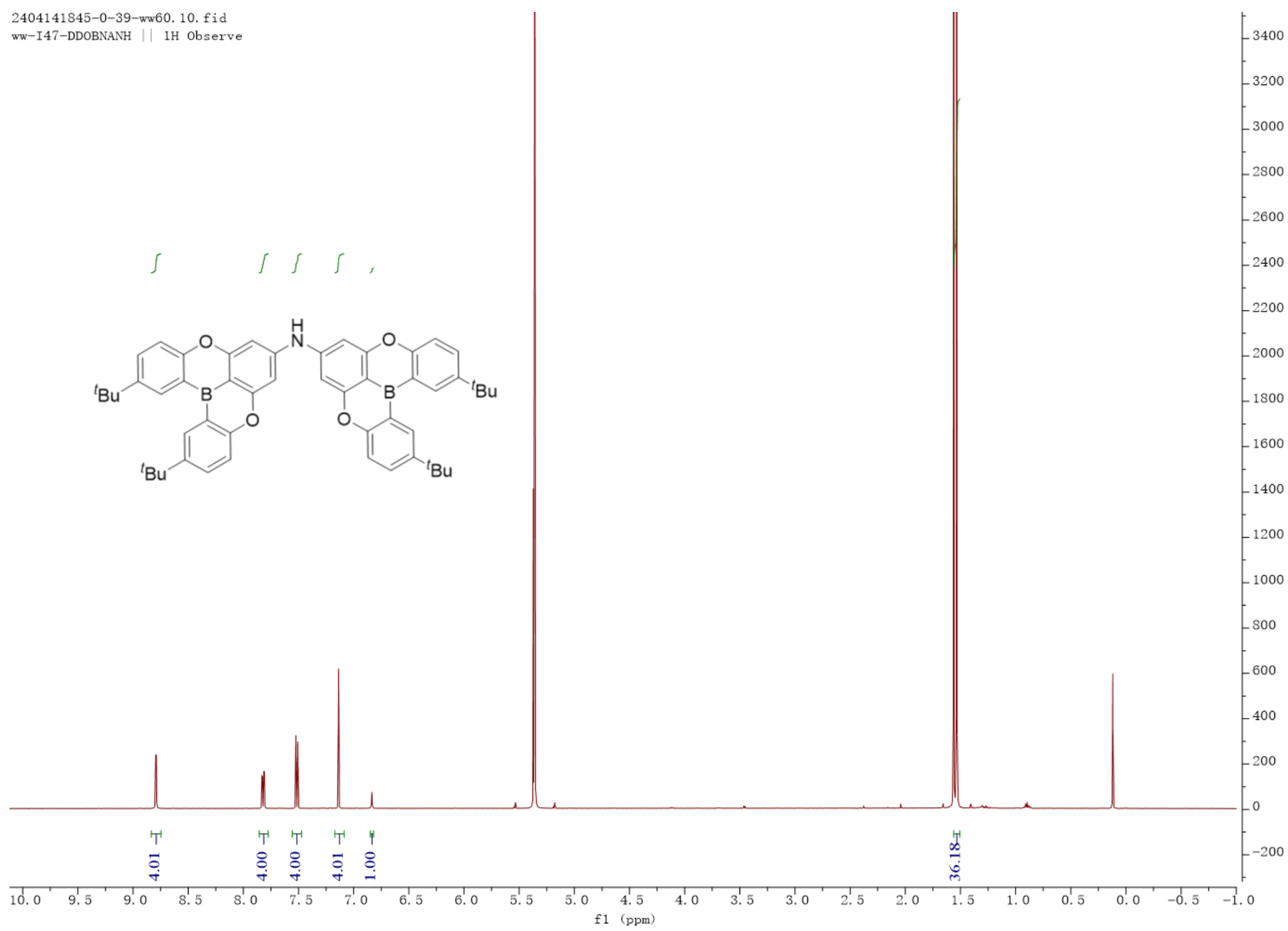


Figure **S16**. $^1$H NMR spectrum of **DDOBNH** in $CD_2Cl_2$

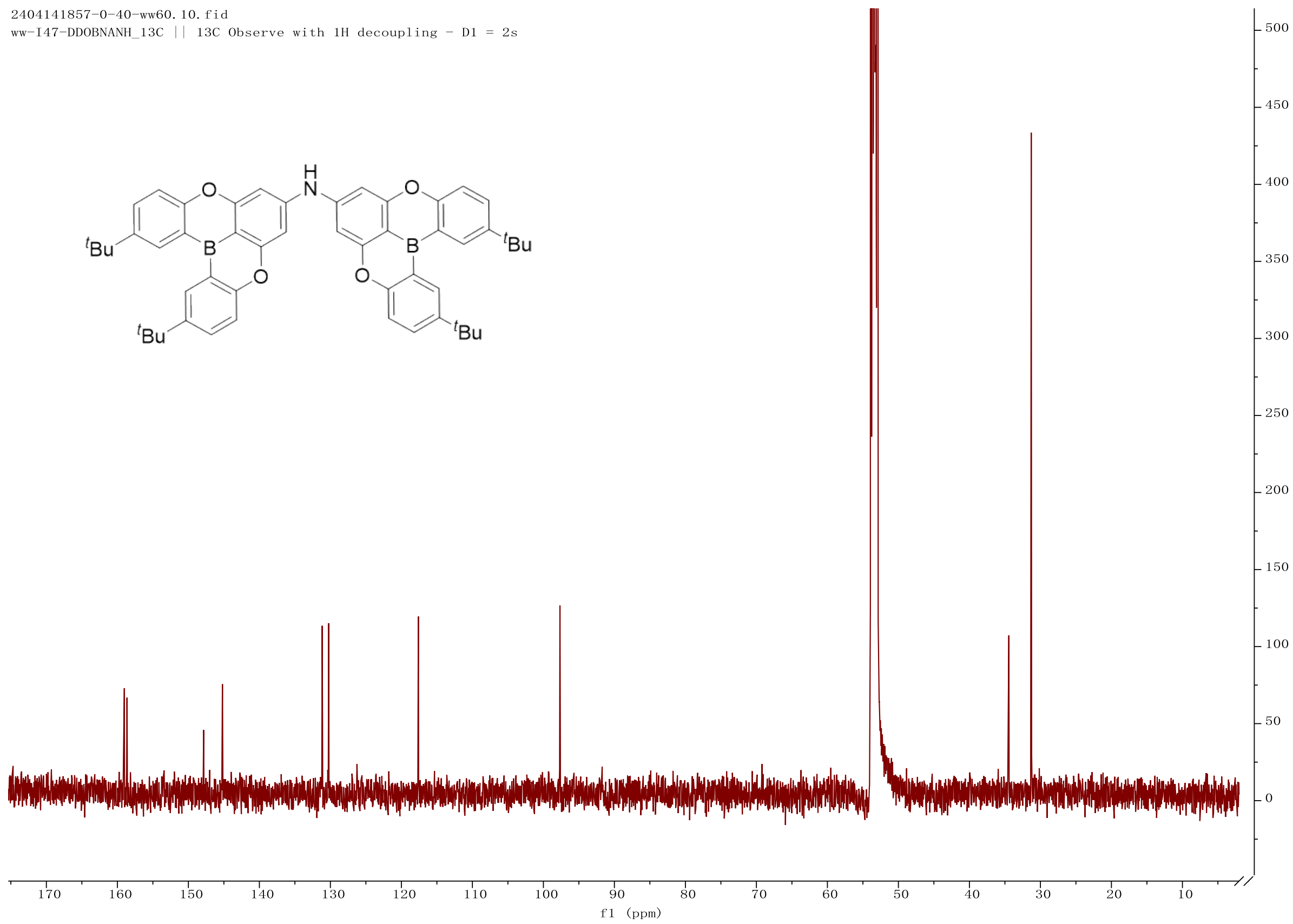


Figure **S17**. $^{13}$C NMR spectrum of **DDOBNH** in $CD_2Cl_2$

## Display Report

**Analysis Info**

| | | | |
|---|---|---|---|
| Analysis Name | D:\Data\Alans Data Sept 2024\STA_Choudhary 20035 (120924) DDOBNH.d | Acquisition Date | 9/12/2024 2:59:44 PM |
| Method | AAlansVGDtune260624_low.m | Operator | demo |
| Sample Name | Choudhary 20035 (120924) DDOBNH | Instrument | micrOTOF 8213750.10408 |
| Comment | Choudhary 20035 (120924) DDOBNH | | |

**Acquisition Parameter**

| | | | | | |
|---|---|---|---|---|---|
| Source Type | ESI | Ion Polarity | Positive | Set Nebulizer | 0.4 Bar |
| Focus | Not active | | | Set Dry Heater | 200 °C |
| Scan Begin | 50 m/z | Set Capillary | 3000 V | Set Dry Gas | 6.0 l/min |
| Scan End | 3000 m/z | Set End Plate Offset | -500 V | Set Divert Valve | Waste |

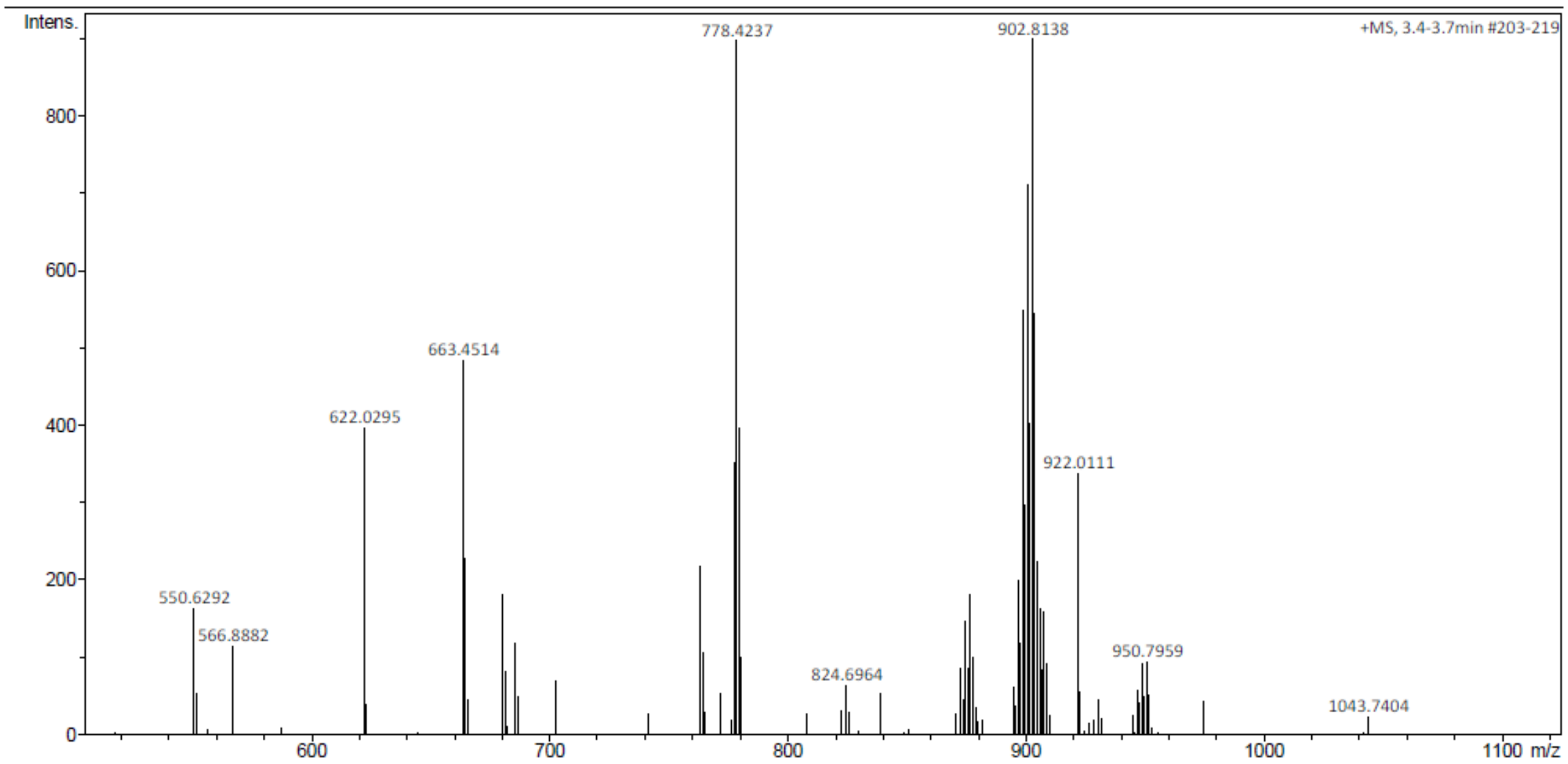


Bruker Compass DataAnalysis 4.1 printed: 9/12/2024 3:05:52 PM by: demo 1 of 1


## Display Report

**Analysis Info**

| | | | |
|---|---|---|---|
| Analysis Name | D:\Data\Alans Data Sept 2024\STA_Choudhary 20035 (120924) DDOBNH.d | Acquisition Date | 9/12/2024 2:59:44 PM |
| Method | AAlansVGDtune260624_low.m | Operator | demo |
| Sample Name | Choudhary 20035 (120924) DDOBNH | Instrument | micrOTOF 8213750.10408 |
| Comment | Choudhary 20035 (120924) DDOBNH | | |

**Acquisition Parameter**

| | | | | | |
|---|---|---|---|---|---|
| Source Type | ESI | Ion Polarity | Positive | Set Nebulizer | 0.4 Bar |
| Focus | Not active | | | Set Dry Heater | 200 °C |
| Scan Begin | 50 m/z | Set Capillary | 3000 V | Set Dry Gas | 6.0 l/min |
| Scan End | 3000 m/z | Set End Plate Offset | -500 V | Set Divert Valve | Waste |


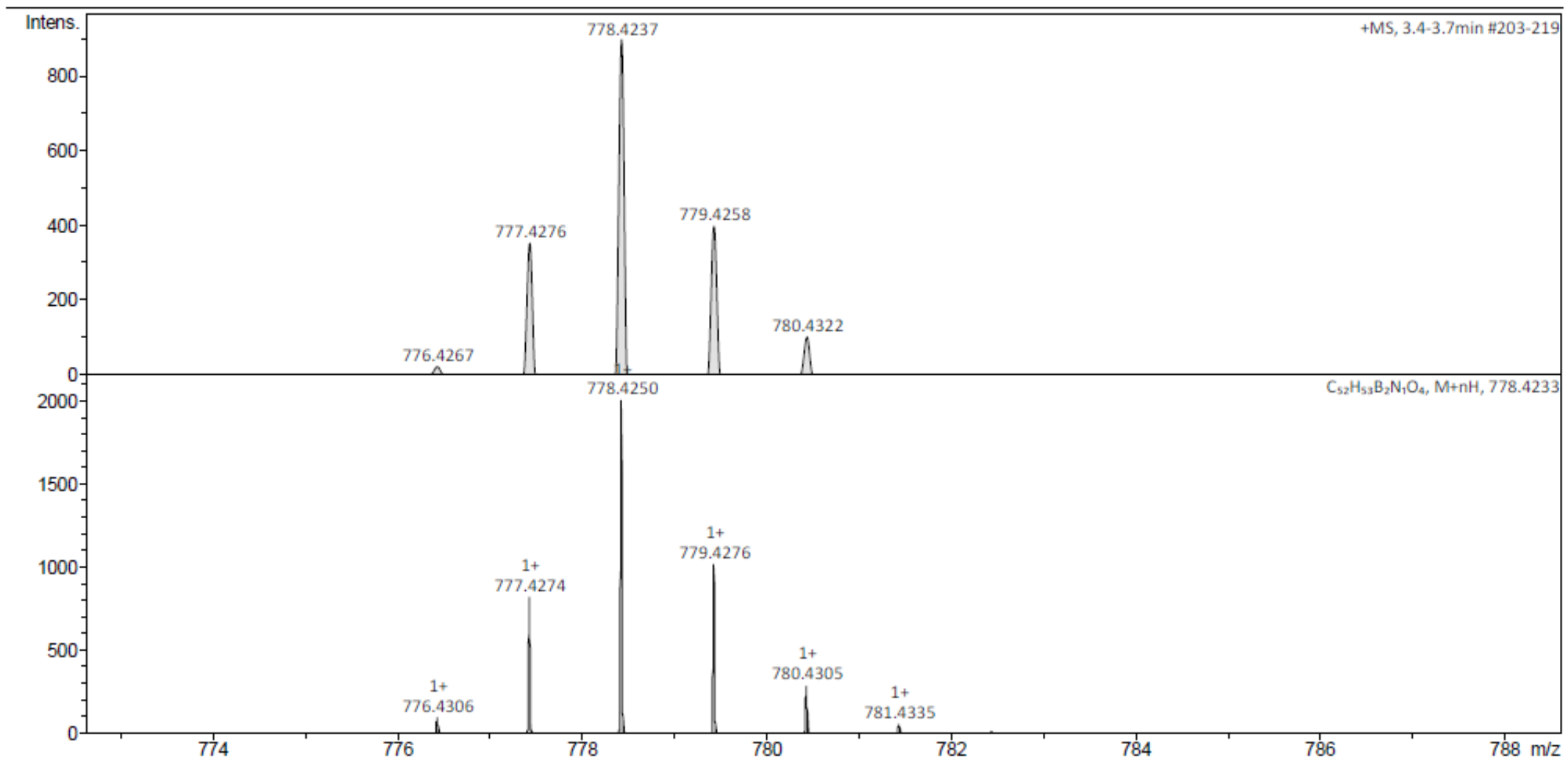


Bruker Compass DataAnalysis 4.1 printed: 9/12/2024 3:07:03 PM by: demo 1 of 1

Figure **S18**. HRMS spectrum of **DDOBNH**

**Dimethyl 2-(bis(2,12-di-*tert*-butyl-5,9-dioxa-13b-boranaphtho[3,2,1-de]anthracen-7-yl)amino)isophthalate (DDOBDE)**

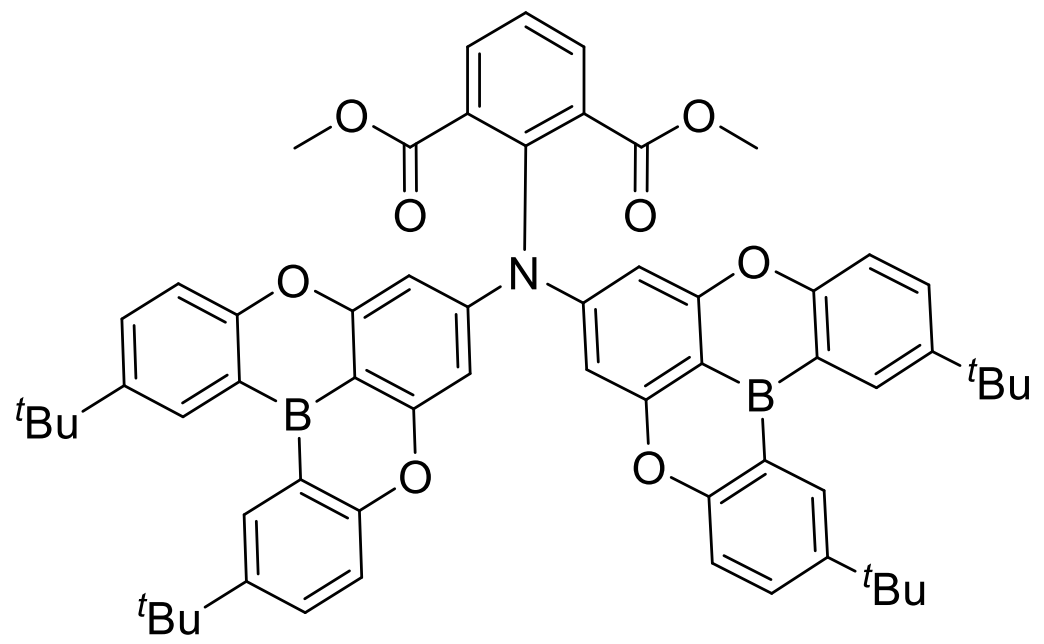


**DDOBNH** (1.00 g, 1.286 mmol, 1.0 equiv.), **Dimethyl 2-bromoisophthalate** (1.40 g, 5.144 mmol, 4.0 equiv.), potassium carbonate (533.2 mg, 3.858 mmol, 3.0 equiv.), 2,2,6,6-tetramethylheptane-3,5-dione (35.5 mg, 192.9 μmol, 0.15 equiv.), copper iodide (36.7 mg, 192.9 μmol, 0.15 equiv.), copper-tin alloy (71.1 mg, 385.8 μmol, 0.3 equiv.) and 30 mL anhydrous dibutyl ether were added to a 100 mL Schlenk tube under nitrogen. The reaction was heated at 160 °C for 4 days. After cooling to room temperature, the mixture was extracted with 3× 150 mL each of brine and 150 mL of DCM, and purified by flash column chromatography (ethyl acetate: hexane =1:4) to afford 0.59 g of the desired product as white powder with yield of 47%. **Mp**: 227-230 °C. **R*f***: 0.40 (EA: hexane = 1:4). **$^{1}$H NMR** (500 MHz, dichloromethane-*d*$_2$) δ 8.78 (d, *J* = 2.5 Hz, 4H), 8.05 (d, *J* = 7.8 Hz, 2H), 7.79 (dd, *J* = 8.8, 2.4 Hz, 4H), 7.63 (t, *J* = 7.8 Hz, 1H), 7.43 (d, *J* = 8.8 Hz, 4H), 6.94 (s, 4H), 3.54 (s, 6H), 1.52 (s, 36H). **$^{13}$C NMR** (126 MHz, dichloromethane-*d*$_2$) δ 166.32, 158.73, 158.16, 151.87, 145.18, 143.14, 134.57, 133.18, 131.30, 130.32, 127.71, 121.70, 117.59, 111.00, 102.31, 34.52, 31.40. **HRMS [M$^+$]**: Calculated: 970.4583 ($C_{62}H_{61}B_2NO_8$); Found 970.4675.

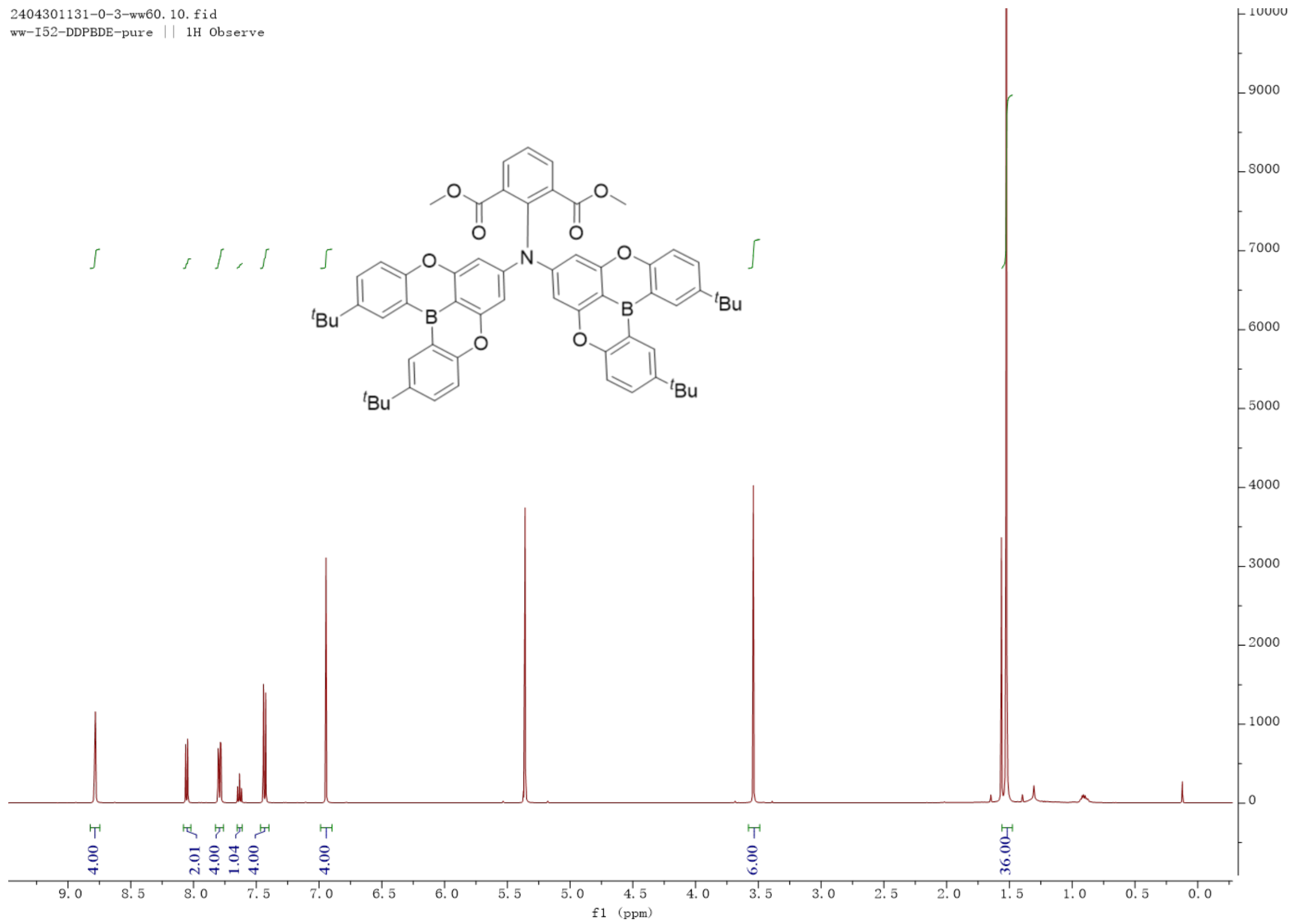


Figure **S19**. $^{1}$H NMR spectrum of **DDOBDE** in $CD_2Cl_2$

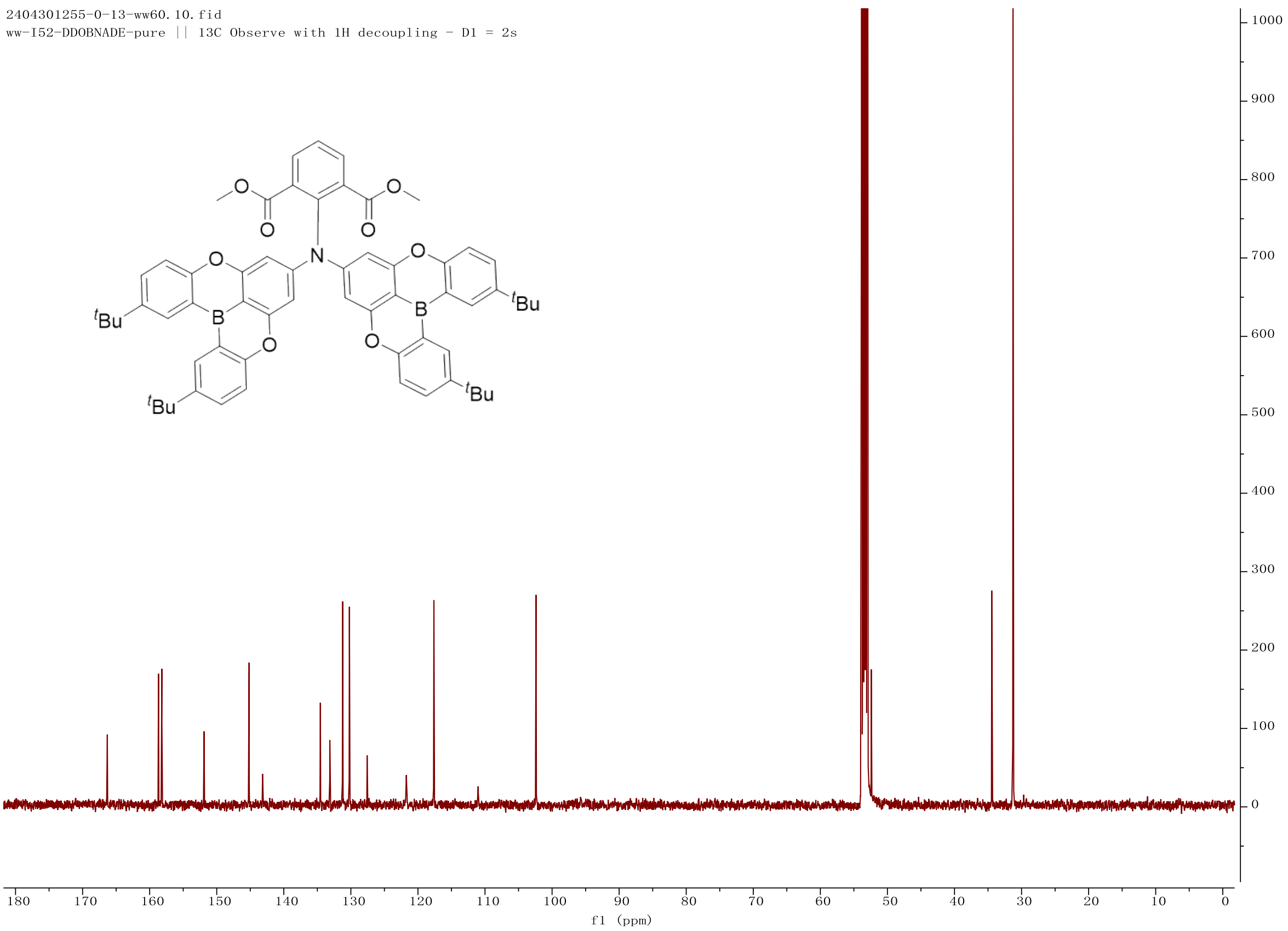


Figure **S20**. $^{13}$C NMR spectrum of **DDOBDE** in $CD_2Cl_2$

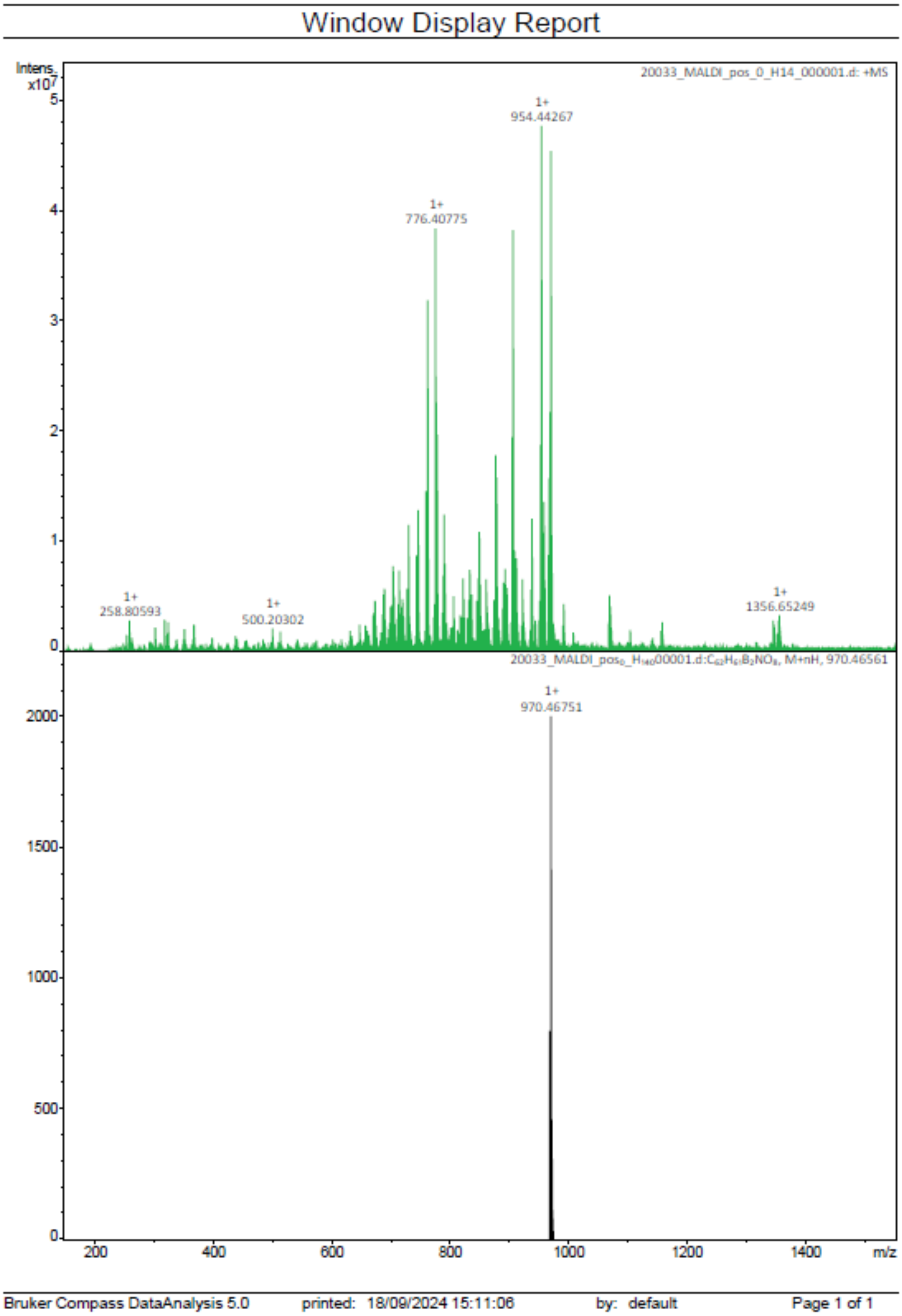


Figure **S21**. HRMS spectrum of **DDOBDE**

**2,14,17,26-tetra-*tert*-butyl-5,11,20,23-tetraoxa-21b-aza-15b,27b-diboranaphtho[1,2,3-qr]naphtho[3',2',1':5,6]tetraceno[3,2,1-de]pentacene-6,10-dione (DDOBDiKTa)**

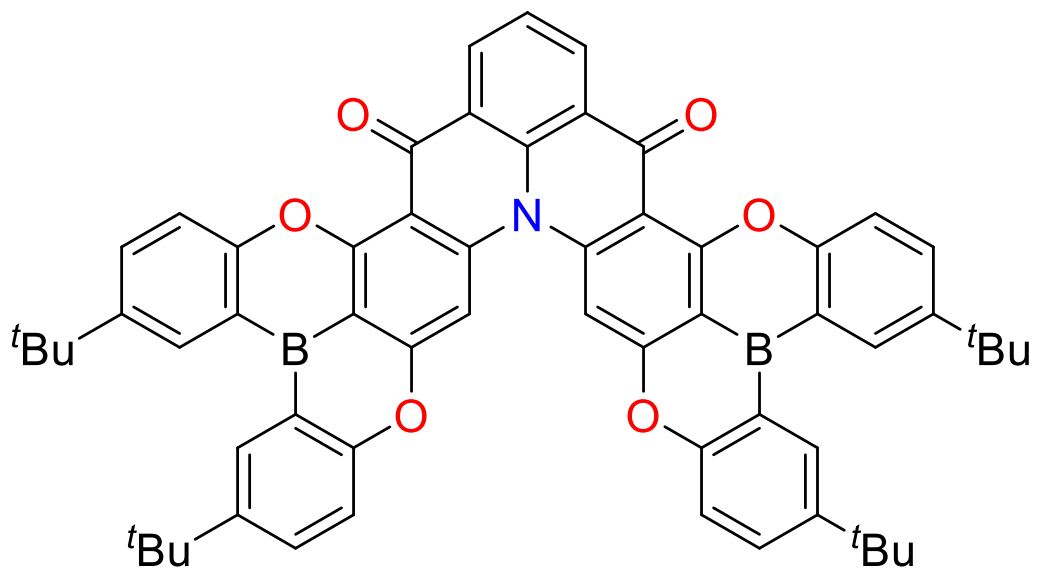


**DDOBDE** (0.70 g, 0.72 mmol, 1.0 equiv.) and sodium hydroxide (173.2 mg, 4.33 mmol, 6.0 equiv.) were added to a flask containing 8 mL of an ethanol/water (1:1) mixture. The reaction was heated at reflux for 16 h. After cooling to room temperature, the pH was adjusted to 2-3 by addition of dilute hydrochloric acid. The white diacid precipitated and was collected by vacuum filtration, washed thoroughly with water, dried under vacuum and used without further purification and characterization. The diacid product (320 mg, 0.34 mmol, 1 equiv.) was added to a two-neck flask, degassed and refilled with nitrogen three times. Then 10 mL of dichloromethane under a nitrogen atmosphere was added, giving a yellow solution. To the reaction mixture were added 15 drops of thionyl chloride (101.1 mg, 0.85 mmol 2.5 equiv.) and 2 drops of DMF dispersed in 1 mL of DCM, giving a dark green solution. Then the reaction was heated to reflux, after 3 h under reflux, the reaction mixture was cooled to room temperature. Under a positive flow of nitrogen, $SnCl_4$ (1.79 mL, 1.79 mmol, 5 equiv.) was added slowly and the mixture was again heated to reflux. After refluxing for 16 h, the reaction mixture was cooled to room temperature and the reaction quenched by the dropwise addition of water. The mixture was combined with dichloromethane (50 mL), and washed with a saturated NaCl aqueous solution (3×50 mL). The organic fractions were combined, and the solvent volume was concentrated under reduced pressure. The crude product was purified by silica gel CombiFlash column chromatography (eluent: DCM: hexane = 95:5) to afford 178 mg of the desired product as a yellow powder in a yield of 58%. **Mp**: 313-315 °C (decomp.). **$R_f$**: 0.42 (EA: hexane = 1:4). **$^1$H NMR** (500 MHz, dichloromethane-$d_2$) δ 8.79 (d, $J$ = 2.4 Hz, 2H), 8.74 (d, $J$ = 2.4 Hz, 2H), 8.60 (d, $J$ = 7.5 Hz, 2H), 8.00 (s, 2H), 7.92 (dd, $J$ = 8.7, 2.4 Hz, 2H), 7.75 (d, $J$ = 8.7 Hz, 2H), 7.69 – 7.64 (m, 3H), 7.29 (d, $J$ = 8.7 Hz, 2H), 1.57 (s, 18H), 1.44 (s, 18H). **$^{13}$C NMR** (126 MHz, dichloromethane-$d_2$) δ 176.33, 159.66, 158.38, 157.79, 146.41, 146.00, 132.09, 131.67, 130.80, 130.51, 129.80, 124.98, 124.02, 118.72, 117.54, 110.17, 100.45, 34.47, 31.30. **HRMS [$M^+$]**: Calculated: 906.4059 ($C_{60}H_{53}B_2NO_6$); Found 906.4150. **HPLC** (100% THF): 99.45% purity, retention time 13.143 min. **Anal. Calcd. For $C_{60}H_{53}B_2NO_6$**: C 79.57%, H 5.90%, N 1.55% Found: C 78.49%, H 6.03%, N 1.41%.

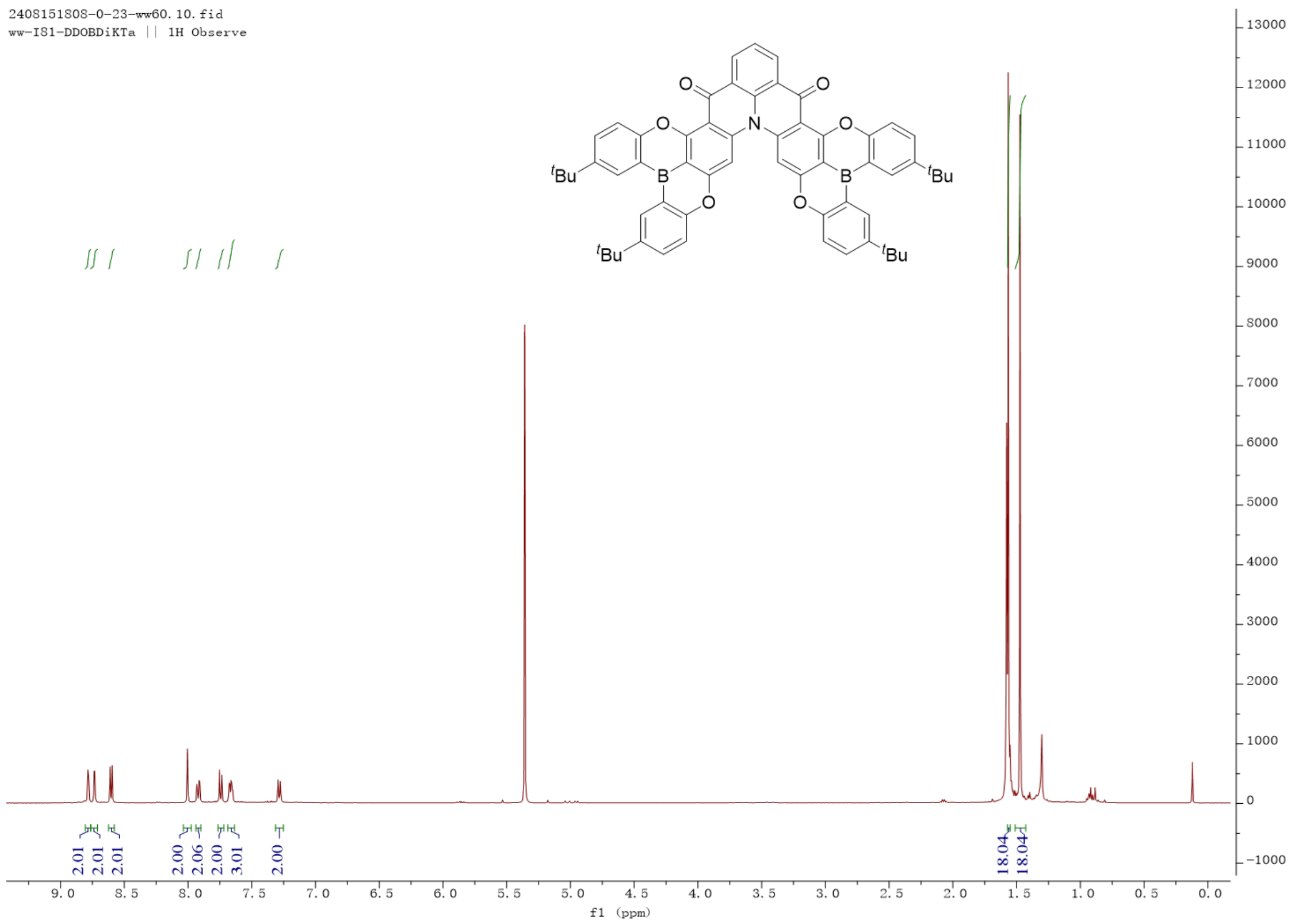


Figure **S22**. $^{1}$H NMR spectrum of **DDOBDiKTa** in $CD_2Cl_2$

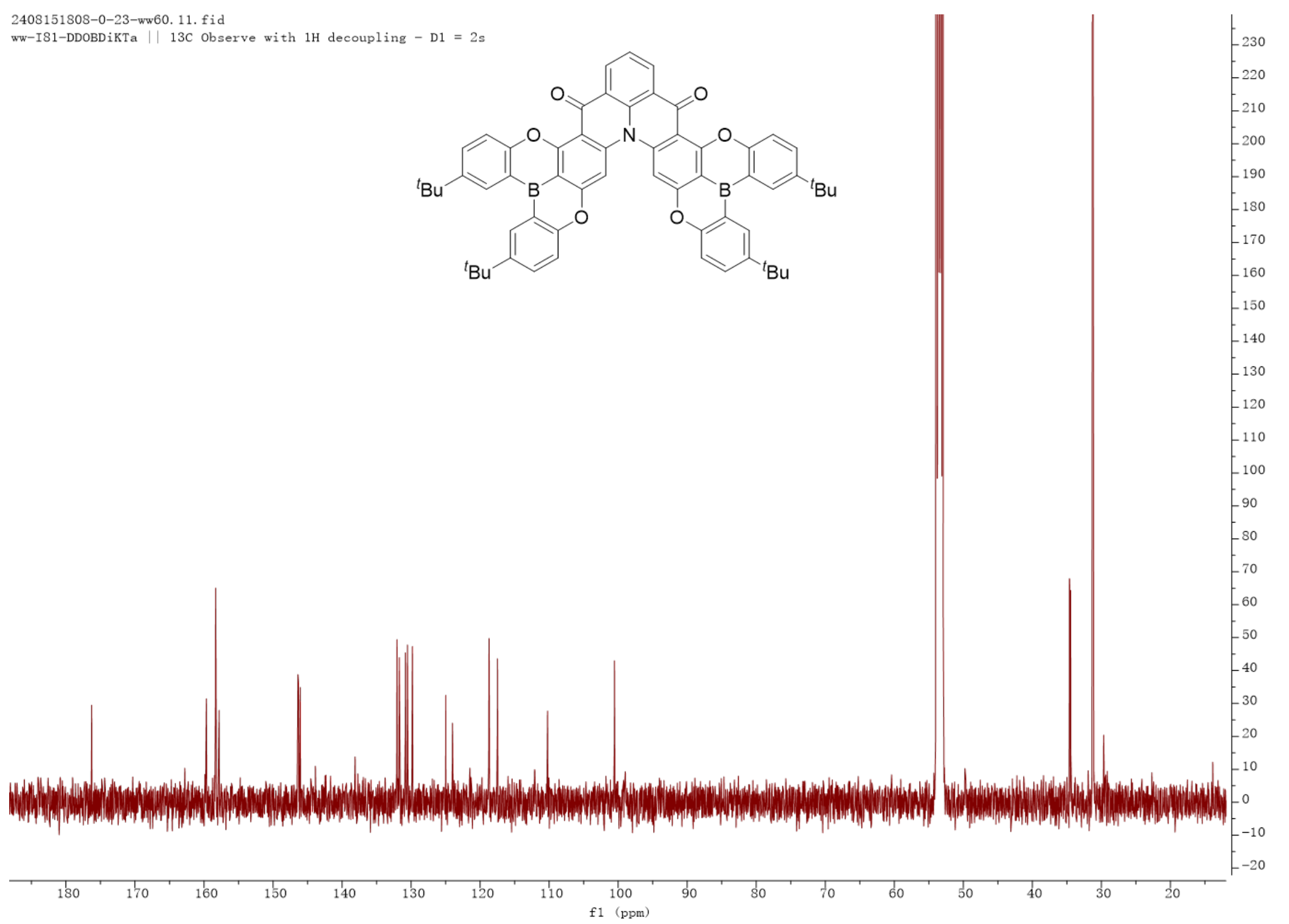


Figure **S23**. $^{13}$C NMR spectrum of **DDOBDiKTa** in $CD_2Cl_2$

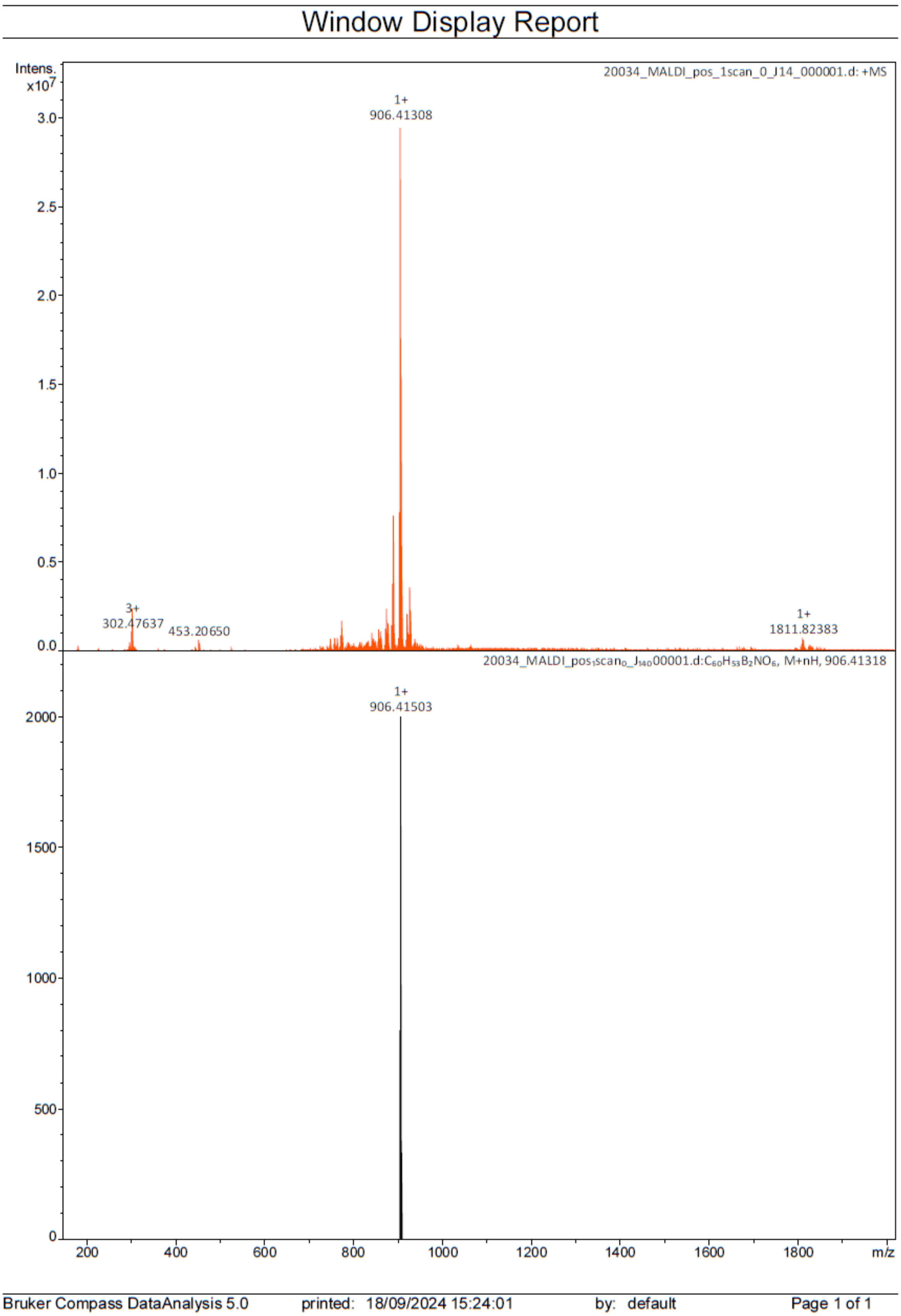


Figure **S24**. HRMS spectrum of **DDOBDiKTa**

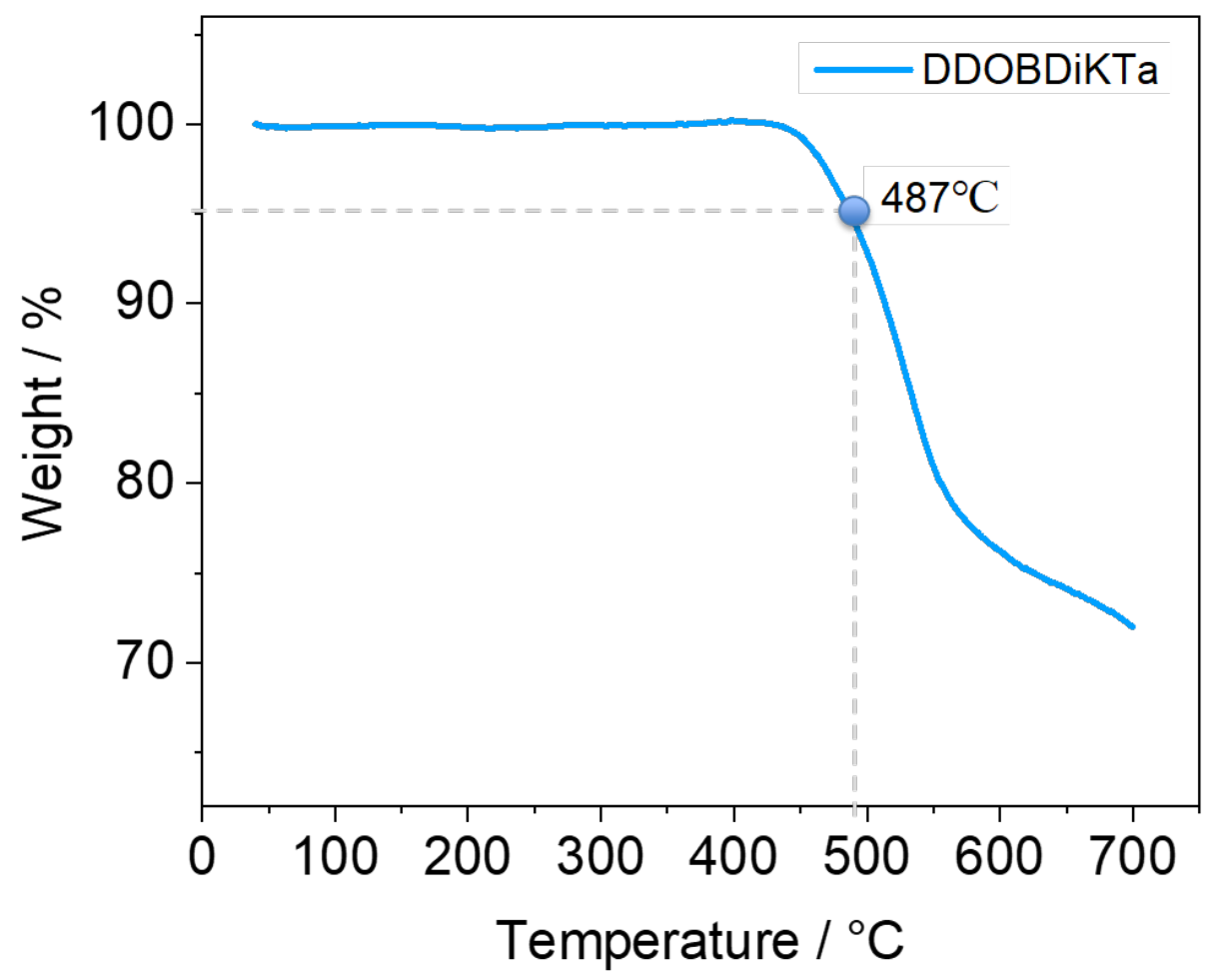


Figure **S25**. Thermogravimetric analysis (TGA) of **DDOBDiKTa**, 5 wt% loss temperature at 487 ℃

# HPLC Trace Report08Oct2024

**<Sample Information>**

| | | | |
|---|---|---|---|
| Sample Name | : DDOBDiKTa | | |
| Sample ID | : | | |
| Method Filename | : 100% THF 20 mins 280nm.lcm | | |
| Batch Filename | : 20241008_GPCprep.lcb | | |
| Vial # | : 1-13 | Sample Type | : Unknown |
| Injection Volume | : 50 uL | | |
| Date Acquired | : 08/10/2024 18:18:54 | Acquired by | : System Administrator |
| Date Processed | : 08/10/2024 18:55:22 | Processed by | : System Administrator |

**<Chromatogram>**

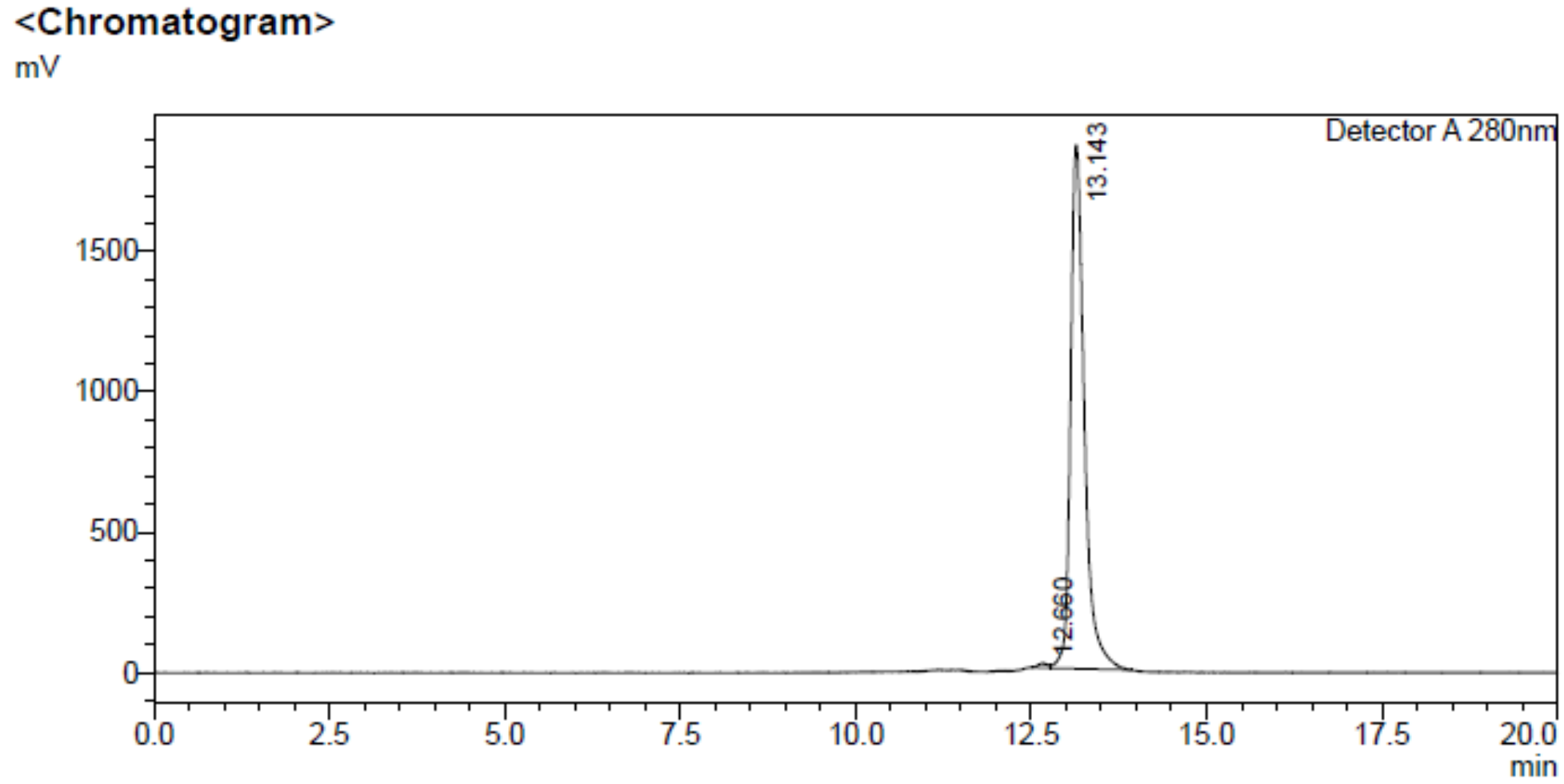


**<Peak Table>**

Detector A 280nm

| Peak# | Ret. Time | Area | Height | Area% | Area/Height | Width at 5% Height |
|---|---|---|---|---|---|---|
| 1 | 12.660 | 141334 | 15096 | 0.550 | 9.363 | -- |
| 2 | 13.143 | 25549252 | 1862598 | 99.450 | 13.717 | 0.517 |
| Total | | 25690585 | 1877694 | 100.000 | | |

Figure **S26**. HPLC spectrum of purity of **DDOBDiKTa**, with 100% THF mobile phase

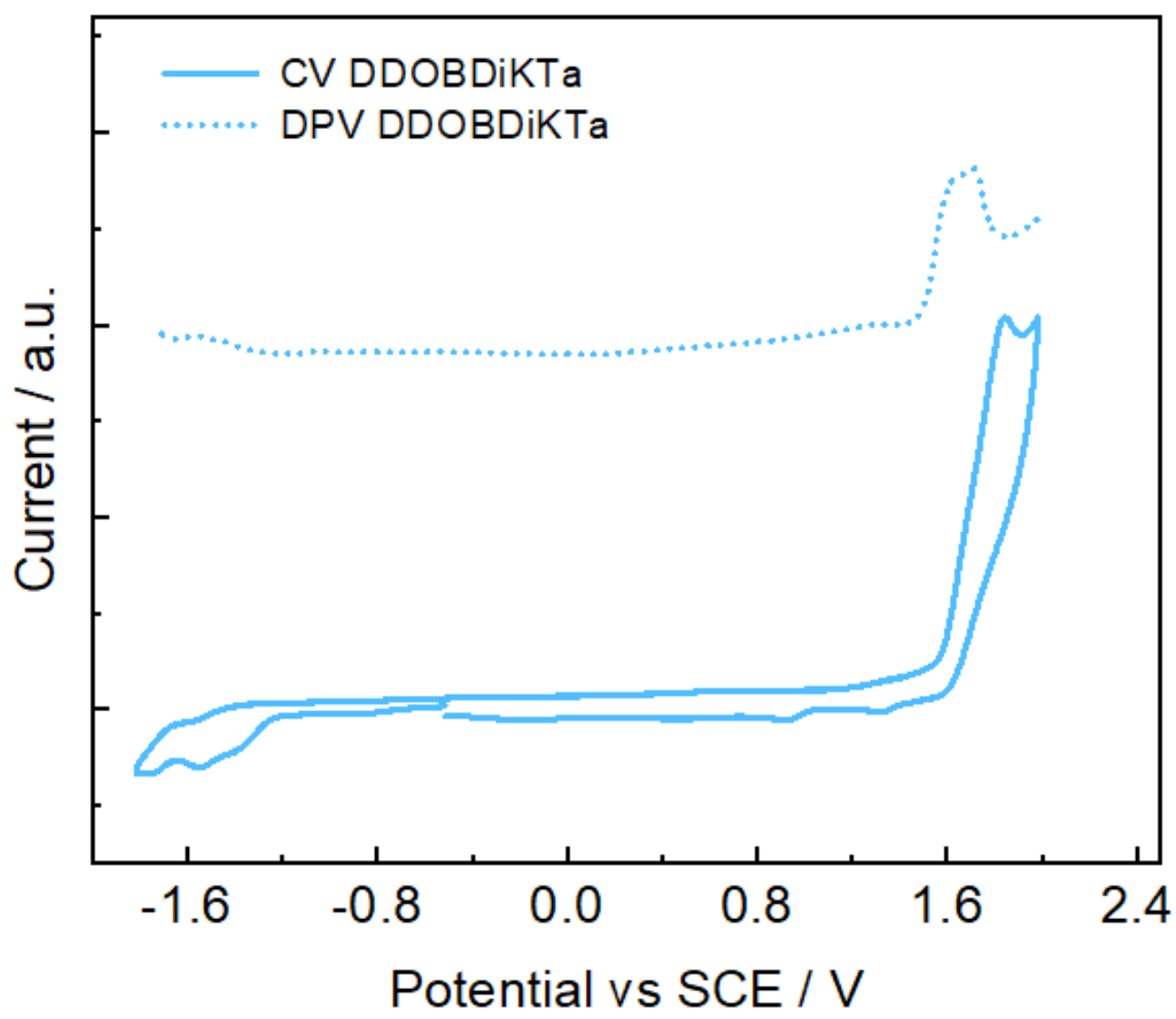


Figure **S27**. Cyclic voltammogram (CV) and differential pulse voltammetry (DPV) in degassed DCM with 0.1 M $[^{S}Bu_4N]PF_6$ as the supporting electrolyte and $Fc/Fc^+$ as the internal reference versus SCE (0.46 V vs. SCE).

Table **S1**. Electrochemical results

| emitter | $E_{OX}$/V[a] | $E_{red}$/V[a] | HOMO/eV[b] | LUMO/eV[b] | $E_g$[c] |
|---|---|---|---|---|---|
| **DDOBDiKTa** | 1.70 | -1.56 | -6.04 | -2.78 | 3.26 |

[a] $E_{ox}$ and $E_{red}$ are the peak of anodic and cathodic potentials from DPV versus SCE. In degassed DCM with 0.1 M $[^{n}Bu_4N]PF_6$ as the supporting electrolyte and $Fc/Fc^+$ as the internal reference (0.46 V vs. SCE). [b] $E_{HOMO/LUMO} = -(E_{ox} / E_{red}$ vs. $Fc/Fc^+ + 4.8)$ eV. [c] $E_g = E_{LUMO} - E_{HOMO}$

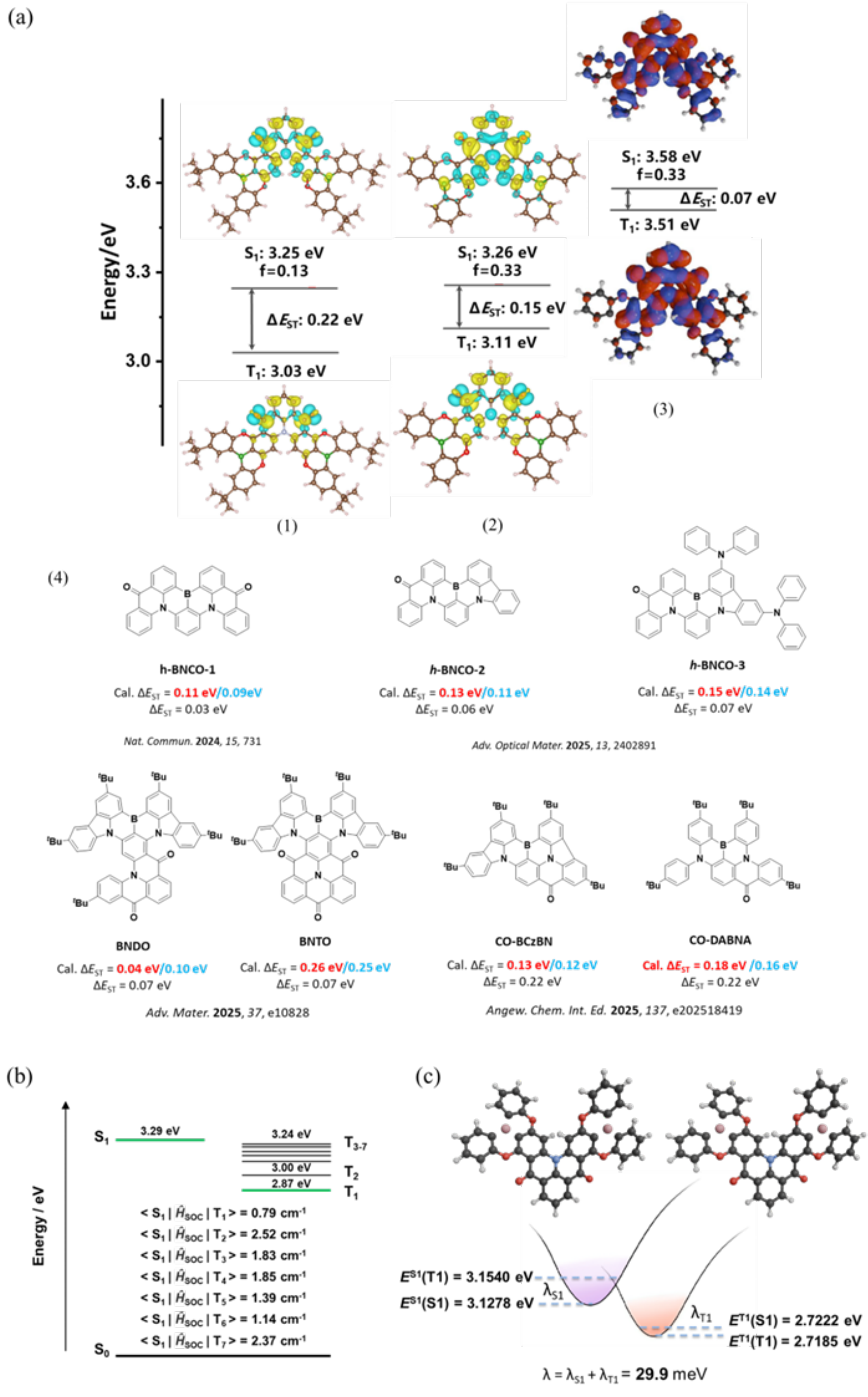

(a)
Energy/eV
3.6
3.3
3.0
S1: 3.25 eV
f=0.13
ΔEST: 0.22 eV
T1: 3.03 eV
S1: 3.26 eV
f=0.33
ΔEST: 0.15 eV
T1: 3.11 eV
S1: 3.58 eV
f=0.33
ΔEST: 0.07 eV
T1: 3.51 eV
(1)
(2)
(3)
(4)
h-BNCO-1
Cal. ΔEST = 0.11 eV/0.09eV
ΔEST = 0.03 eV
Nat. Commun. 2024, 15, 731
h-BNCO-2
Cal. ΔEST = 0.13 eV/0.11 eV
ΔEST = 0.06 eV
h-BNCO-3
Cal. ΔEST = 0.15 eV/0.14 eV
ΔEST = 0.07 eV
Adv. Optical Mater. 2025, 13, 2402891
BNDO
Cal. ΔEST = 0.04 eV/0.10 eV
ΔEST = 0.07 eV
BNTO
Cal. ΔEST = 0.26 eV/0.25 eV
ΔEST = 0.07 eV
Adv. Mater. 2025, 37, e10828
CO-BCzBN
Cal. ΔEST = 0.13 eV/0.12 eV
ΔEST = 0.22 eV
CO-DABNA
Cal. ΔEST = 0.18 eV /0.16 eV
ΔEST = 0.22 eV
Angew. Chem. Int. Ed. 2025, 137, e202518419
(b)
Energy / eV
S1
3.29 eV
3.24 eV
T3-7
3.00 eV
T2
2.87 eV
T1
< S1 | ĤSOC | T1 > = 0.79 cm-1
< S1 | ĤSOC | T2 > = 2.52 cm-1
< S1 | ĤSOC | T3 > = 1.83 cm-1
< S1 | ĤSOC | T4 > = 1.85 cm-1
< S1 | ĤSOC | T5 > = 1.39 cm-1
< S1 | ĤSOC | T6 > = 1.14 cm-1
< S1 | ĤSOC | T7 > = 2.37 cm-1
S0
(c)
ES1(T1) = 3.1540 eV
ES1(S1) = 3.1278 eV
λS1
λT1
ET1(S1) = 2.7222 eV
ET1(T1) = 2.7185 eV
λ = λS1 + λT1 = 29.9 meV

Figure **S28**. (a) Difference density plots of the $S_1$ and $T_1$ excited states, calculated at the SCS-ADC(2)/cc-pVDZ (a1) and SCS-CC2/cc-pVDZ (a2) levels. Natural Transition Orbitals plots of $S_1$ and $T_1$ excited states, calculated at the RI-wPBEPP86/cc-pVDZ level (a3). (b) Spin-orbit coupling matrix element (SOCME) of **DDOBDiKTa** calculated at the PBE0/6-31G(d,p) level based on the optimized T1 geometry. Summary of the calculated $\Delta E_{ST}$ (SCS-CC(2)/cc-pVDZ level in red, SCS-ADC(2)/cc-pVDZ level in blue) and the experimental values of the reported hybrid molecules, in black (a4). (c) The reorganization energy calculated at PBE0 (GD3BJ) /6-31G(d).

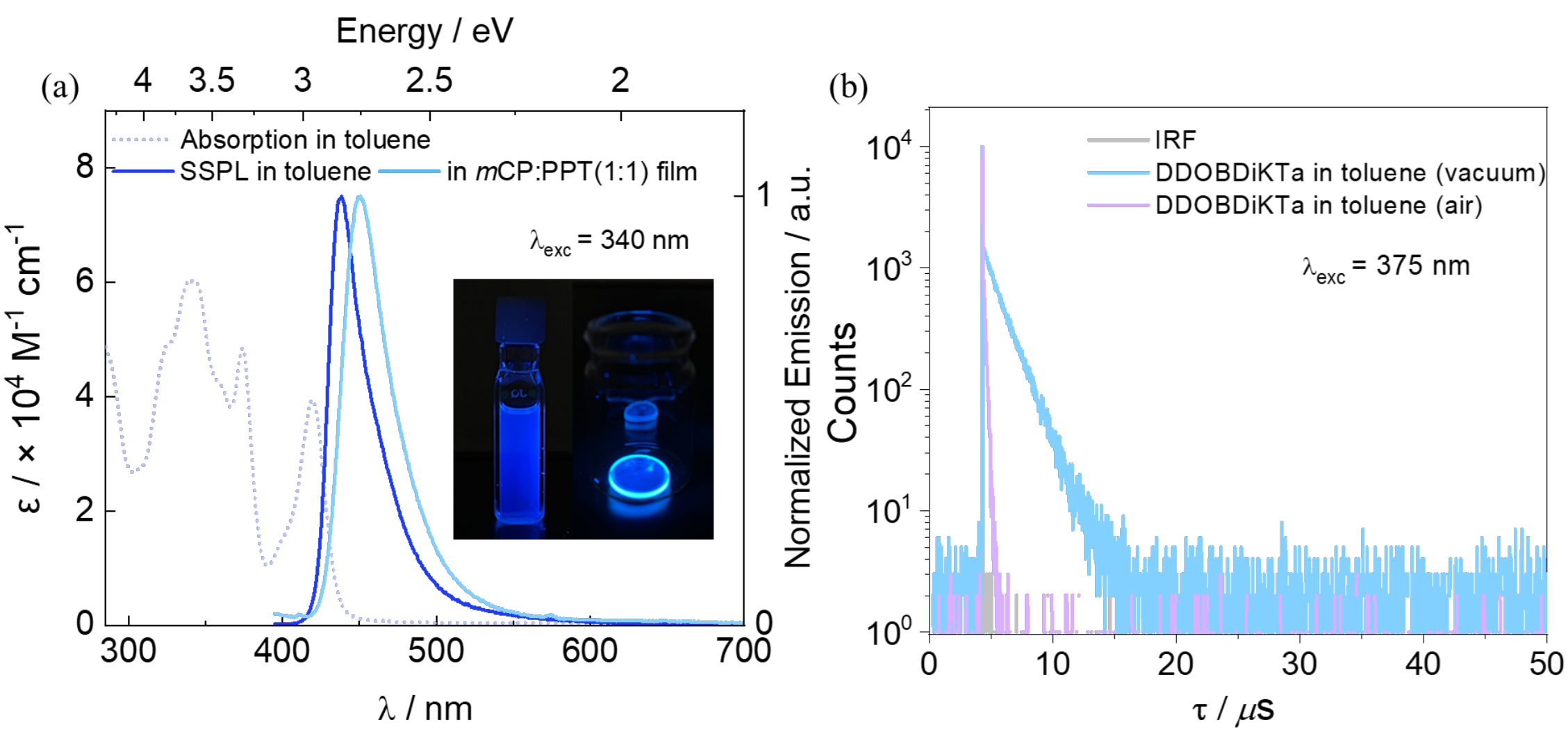


Figure **S29**. (a) The overlay of absorption and emission in toluene and emission in 1.5 wt% doped in *m*CP:PPT film of **DDOBDiKTa** at room temperature, (b) Time-resolved PL decay measured by TCSPC of **DDOBDiKTa** in toluene at room temperature under vacuum and air condition. ($\lambda_{exc}$ = 375 nm).

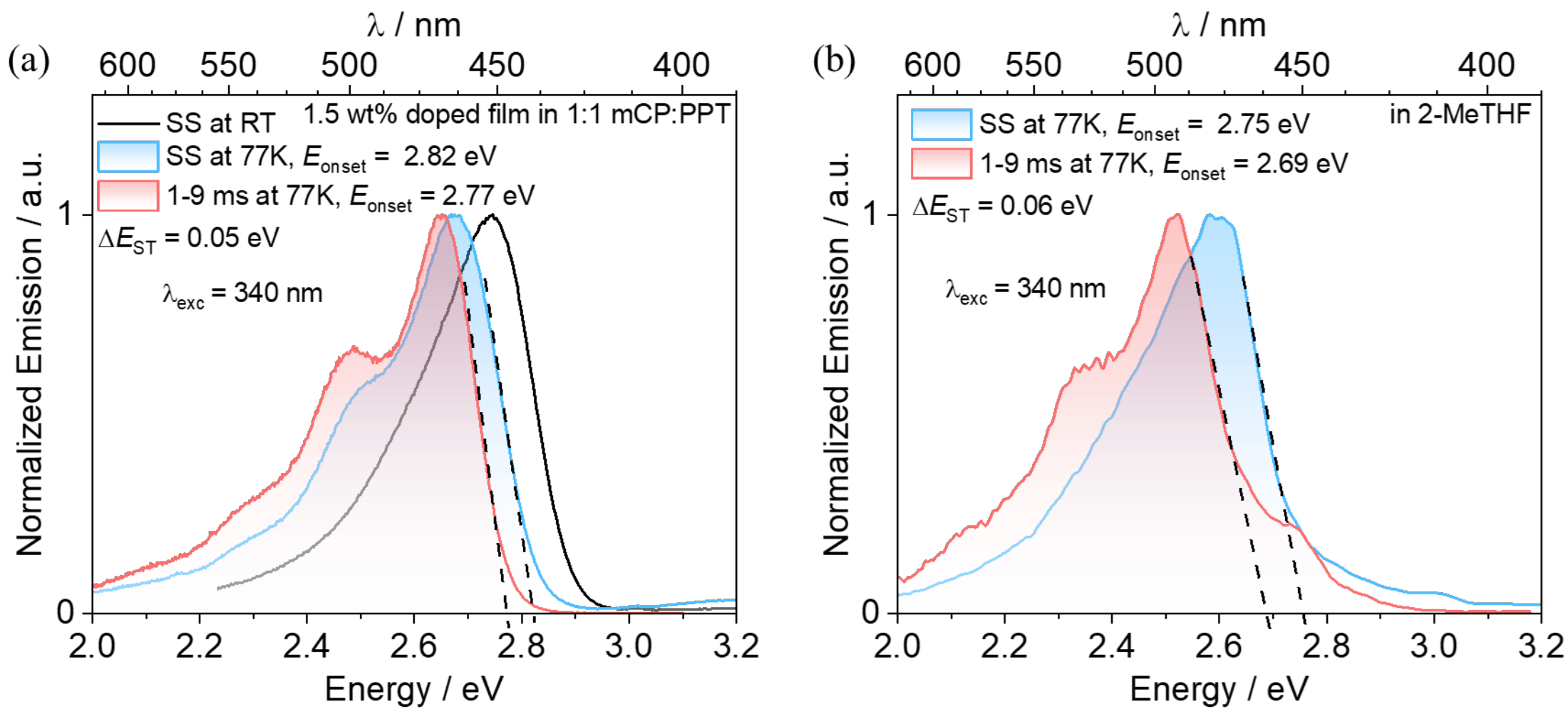


Figure **S30**. (a) Steady state PL and delayed emission spectra in 1.5 wt% doped film in 1:1 *m*CP:PPT and (b) measured in 2-MeTHF

Table **S2**. The singlet-triplet splitting energy $\Delta E_{ST}$ summaries of **DDOBDiKTa**

| | | $S_1$ / eV | $T_1$ / eV | $\Delta E_{ST}$ / eV |
|---|---|---|---|---|
| Sol. | 2-Me THF | 2.75 | 2.69 | 0.06 |
| Film | 1.5 wt% doped in mCP:PPT (1:1) | 2.82 | 2.77 | 0.05 |
| | 5 wt% doped in 26DCzPPy | 2.80 | 2.75 | 0.05 |

Table **S3**. $\Phi_{PL}$ in *m*CP:PPT (1:1) films of **DDOBDiKTa**

| Conc. / wt% | 1 | 1.5 | 2 | 5 | 10 |
|---|---|---|---|---|---|
| $\Phi_{PL}$ / % | 69 | 73 | 68 | 54 | 35 |

Measured using an integrating sphere under positive nitrogen flow at room temperature, $\lambda_{exc}$ = 340 nm.

Table **S4**. Reproducibility verification of solid-state $\Phi_{PL}$ in 5 wt% doped films of **DDOB-DiKTa** in 26DCzPPy

| 5 wt% dope 26DCzPPy films | $\Phi_{PL}$ / % | Average $\Phi_{PL}$ / % | Standard Deviation |
|---|---|---|---|
| Film a | 69 | 70 | 1% |
| Film b | 70 | | |
| Film c | 71 | | |

Measured using an integrating sphere under positive nitrogen flow at room temperature, $\lambda_{exc}$ = 340 nm.

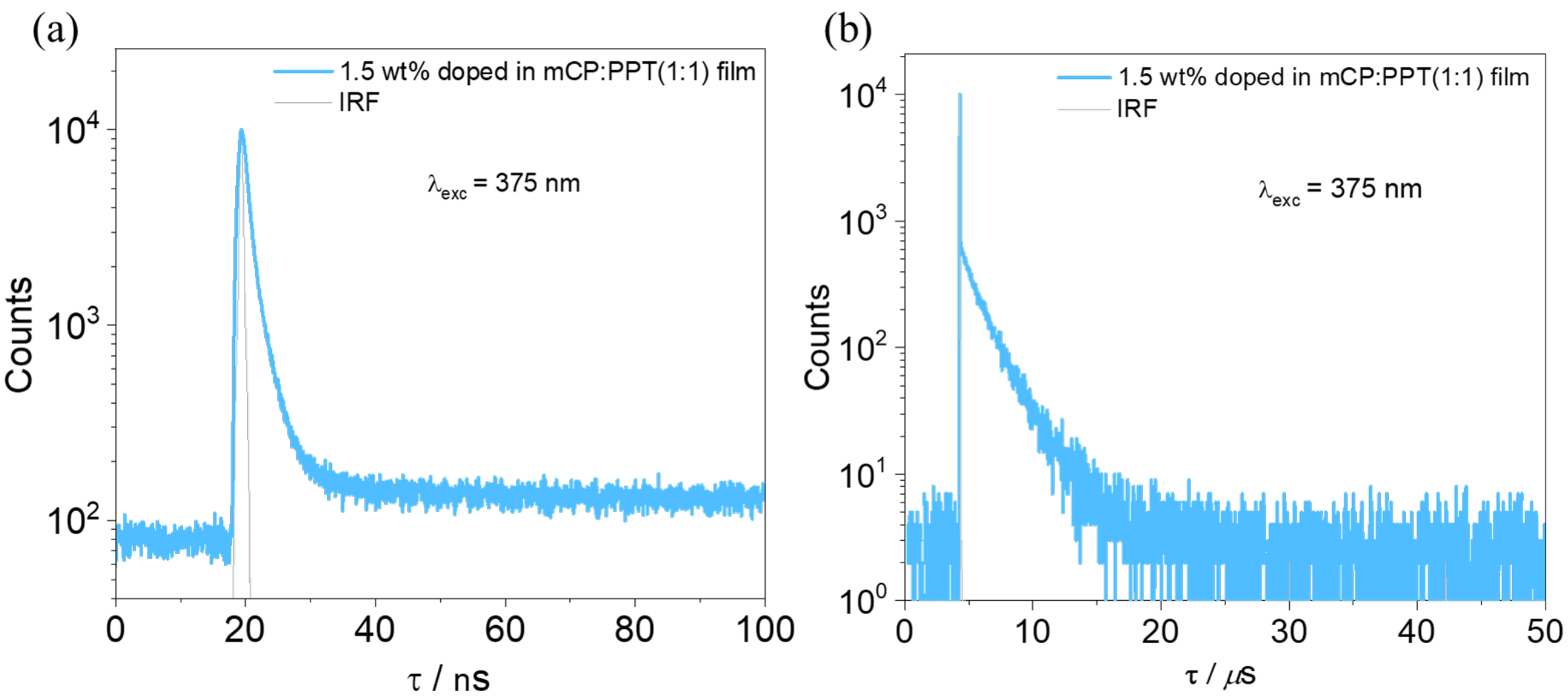


Figure **S31**. Time-resolved PL decays measured by TCSPC of the 1.5 wt% doped film of **DDOBDiKTa** in cohost (*m*CP:PPT, 1:1) at room temperature under vacuum. (a) prompt fluorescence, (b) delayed fluorescence. ($\lambda_{exc}$ = 375 nm).

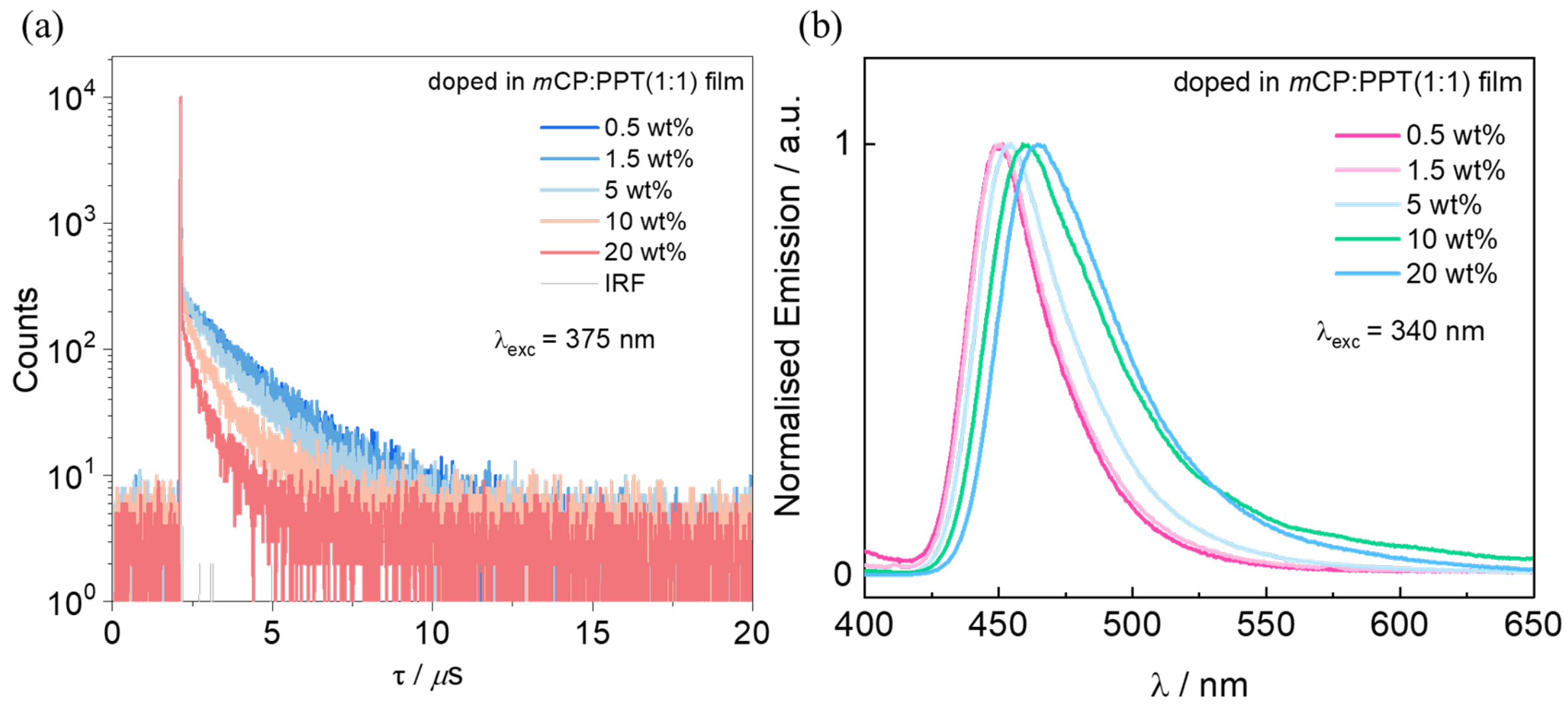


Figure **S32**. (a) Time-resolved PL decay ($\lambda_{exc}$ = 375 nm) and (b) steady-state PL spectra ($\lambda_{exc}$ = 340 nm) of films of various doping concentration of **DDOBDiKTa** in cohost (*m*CP:PPT, 1:1) at room temperature.

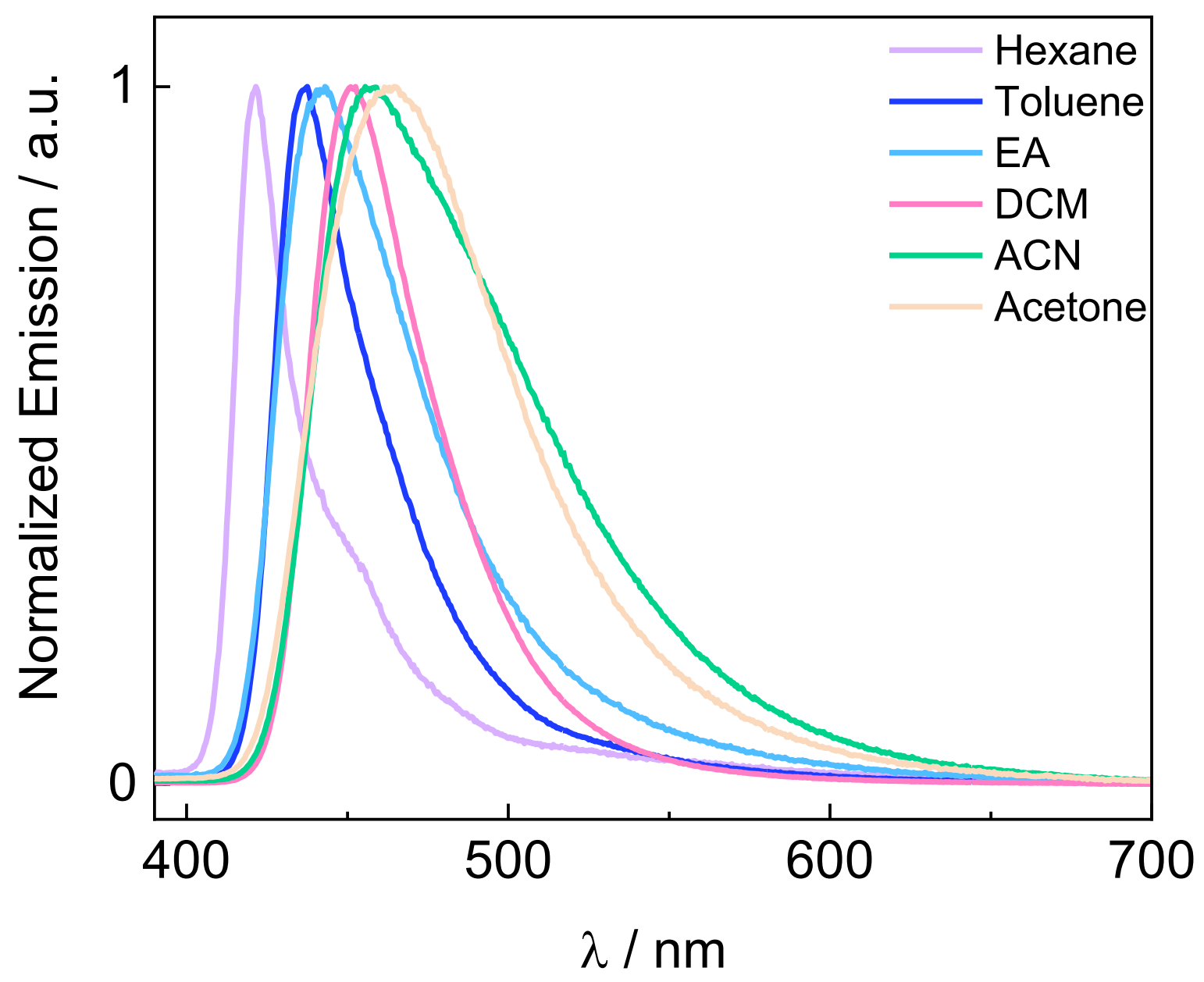


Figure **S33**. Solvatochromism steady-state PL study for **DDOBDiKTa** in solvents of differing polarity ($\lambda_{exc}$ = 340 nm) at room temperature.

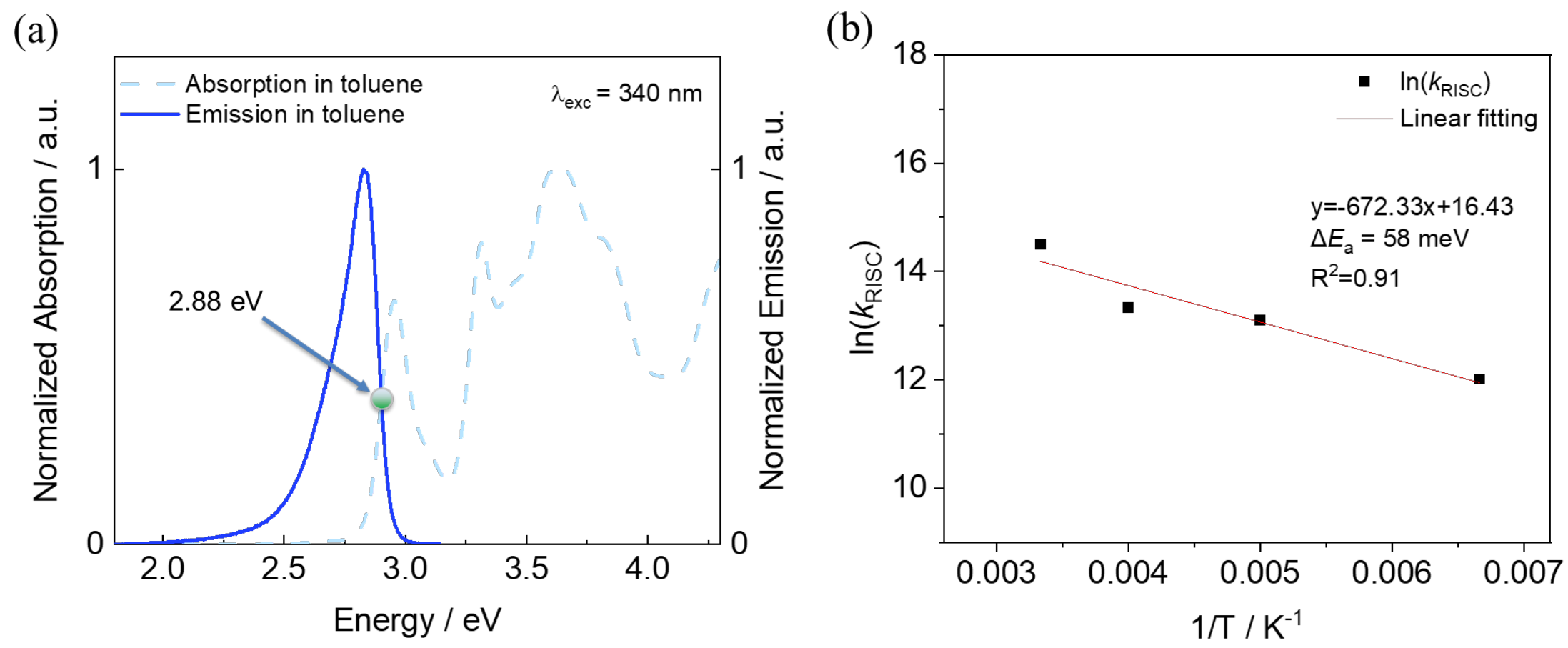


Figure **S34**. (a) Absorption and SS PL spectra of **DDOBDiKTa** in toluene ($\lambda_{exc}$ = 340 nm) after Jacobian transformation at room temperature (b) Arrhenius plot of the 5 wt% doped film of **DDOBDiKTa** in 2,6-DCzPPy, $\lambda_{exc}$ = 375 nm.

The kinetics parameters were calculated according to the following equations and summarized in Table **S5**. [38]

$$\Phi_{PL} = \Phi_P + \Phi_d \tag{S1}$$

$$k_P = \frac{1}{\tau_P} \tag{S2}$$

$$k_d = \frac{1}{\tau_d} \tag{S3}$$

$$k_r^S = k_p \Phi_P \tag{S4}$$

$$k_{ISC} = k_p(1 - \Phi_P) \tag{S5}$$

$$k_{RISC} = \frac{k_p k_d}{k_{ISC}} \frac{\Phi_d}{\Phi_P} \tag{S6}$$

Where the $\Phi_p$ and $\Phi_d$ are the prompt fluorescent and delayed fluorescent quantum efficiency, which are calculated by integrating the transient PL curves; $k_p$ is the rate constant of prompt fluorescence; $k_d$ is the rate constant of delayed fluorescence; $k_r^S$ is the radiative decay rate constant of $S_1$; $k_{ISC}$ is the intersystem crossing rate constant; $k_{RISC}$ is the reverse intersystem crossing rate constant.

Table **S5**. Kinetics parameters of **DDOBDiKTa**

| DDOBDiKTa | $\Phi_{PL}$/ % | $\Phi_{P}$/ % | $\Phi_{d}$/ % | $\tau_p$/ns | $\tau_d$/μs | $k_p$ /$10^8$ $s^{-1}$ | $k_d$ /$10^5$ $s^{-1}$ | $k_r^S$ / $10^7$ $s^{-1}$ | $k_{nr}^T$ / $10^5$ $s^{-1}$ | $k_{ISC}$ / $10^8$ $s^{-1}$ | $k_{RISC}$ / $10^6$ $s^{-1}$ |
|---|---|---|---|---|---|---|---|---|---|---|---|
| Toluene | 30 | 3 | 27 | 0.77 | 1.54 | 13.0 | 6.49 | 3.67 | 4.68 | 12.6 | 6.43 |
| 1.5 wt% in *m*CP:PPT(1:1) | 73 | 12 | 61 | 1.9 | 1.88 | 5.18 | 5.32 | 5.98 | 1.60 | 4.58 | 3.22 |
| 5 wt% in 26DCzPPy | 70 | 21 | 49 | 2.3 | 1.48 | 4.44 | 6.76 | 9.41 | 2.57 | 3.50 | 1.98 |

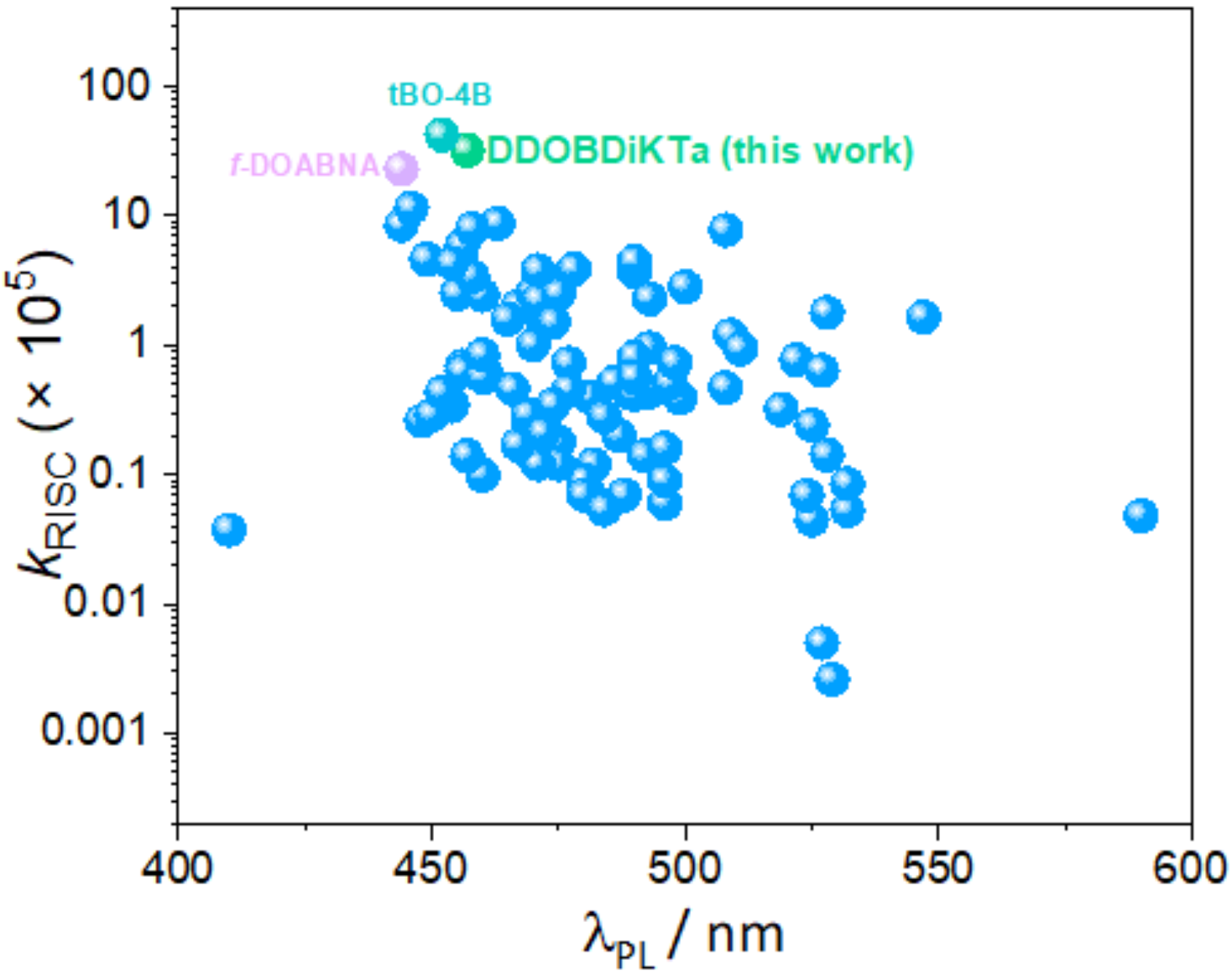


Figure **S35**. A summary of $\lambda_{PL}$ and $k_{RISC}$ values for selected MR-TADF emitters that do not contain heavy atoms (data for this figure are provided in Table **S6**).

Table **S6**. Data table from which Figure **S35** was derived.

| Emitter | $\lambda_{PL}$ / nm | $k_{RISC}$ /$10^5$ $s^{-1}$ | Reference |
|---|---|---|---|
| **DABNA-1** | 460 | 0.099 | 39 |
| **DABNA-2** | 469 | 0.15 | |
| **v-DABNA** | 467 | 2.00 | 40 |
| **TW-BN** | 488 | 0.07 | 41 |
| **TPh-BN** | 492 | 0.14 | |
| **PCz-BN** | 496 | 0.06 | |
| **mCz-BN** | 496 | 0.16 | |
| **3F-BN** | 499 | 0.39 | 42 |
| **4F-BN** | 493 | 0.44 | |

| | | | |
|---|---|---|---|
| **CzBNO** | 454 | 0.34 | 43 |
| **DMAcBNO** | 472 | 0.14 | |
| **DPAcBNO** | 468 | 0.17 | |
| **ν-DABNA-O-Me** | 465 | 1.60 | 44 |
| **DABNA-NP-TB** | 457 | 0.14 | 45 |
| **Cz2DABNA-NP-M/TB** | 477 | 0.73 | |
| **CzB2-M/P** | 497 | 0.51 | |
| **OBN-2CN-BN** | 496 | 0.09 | 46 |
| **BBCz-DB** | 469 | 0.22 | 47 |
| **BBCz-SB** | 487 | 0.20 | |
| **γ-Cb-B** | 461 | 0.58 | 48 |
| **Cz-B** | 482 | 0.12 | |
| **BOBS-Z** | 456 | 4.30 | 49 |
| **BSBS-Z** | 463 | 8.80 | |
| **PAB** | 456 | 0.69 | 50 |
| **2tPAB** | 456 | 0.63 | |
| **3tPAB** | 460 | 0.57 | |
| **B-O-Cz** | 481 | 0.40 | 51 |
| **B-O-dmAc** | 475 | 0.18 | |
| **B-O-dpAc** | 473 | 0.31 | |
| **ADBNA-Me-Mes** | 481 | 0.07 | 52 |
| **ADBNA-Me-Tip** | 480 | 0.09 | |
| **DPACzBN1** | 475 | 0.12 | 53 |
| **DPACzBN2** | 469 | 0.29 | |
| **DPACzBN3** | 472 | 0.21 | |
| **CzBN** | 471 | 0.12 | 54 |
| **CzBN-Cz** | 480 | 0.07 | |
| **TPD4PA** | 455 | 2.51 | 55 |
| **tBu-TPD4PA** | 460 | 2.44 | |
| **h-BNCO-1** | 528 | 1.79 | 56 |
| **f-DOABNA** | 444 | 23.0 | 57 |
| **NOBNacene** | 410 | 0.04 | 58 |
| **m-DBCz** | 547 | 1.65 | 59 |
| **ν-DABNA** | 470 | 2.55 | 60 |
| **Cz-DABNA** | 471 | 1.79 | |
| **t-BuCz-DABNA** | 471 | 2.24 | |
| **NO-DBMR** | 470 | 1.01 | 61 |
| **Cz-DBMR** | 490 | 3.72 | |
| **ω-DABNA** | 509 | 1.20 | 62 |
| **ν-DABNA-Az3** | 458 | 3.40 | 63 |
| **BNCz-pTPA** | 490 | 4.45 | 64 |
| **BNCz-mTPA** | 493 | 2.29 | |
| **CzBN-tDPA** | 478 | 3.87 | 65 |
| **CzBN-mCP** | 475 | 2.52 | |
| **Cz-DBCz** | 489 | 0.43 | 66 |
| **Cz-DBTPA** | 484 | 0.28 | |

| | | | |
|---|---|---|---|
| **PhO-DBCz** | 477 | 0.45 | |
| **DOBDiKTa** | 460 | 0.84 | 67 |
| **DDOBDiKTa** | 457 | 3.22 | This work |
| **DBNO** | 450 | 0.29 | 68 |
| **DMBNO** | 449 | 4.62 | |
| **CNBN** | 508 | 0.47 | 69 |
| **MCNBN** | 519 | 0.32 | |
| **PCNBN** | 511 | 0.94 | |
| **PMCNBN** | 522 | 0.77 | |
| **BO5TCz** | 456 | 5.90 | 70 |
| **MBO5TCz** | 454 | 4.30 | |
| **IDCz-B-I** | 486 | 0.52 | 71 |
| **IDCz-B-II** | 490 | 0.81 | |
| **tBN-Trz** | 525 | 0.0446 | 72 |
| **tBN-NPTrz** | 527 | 0.005 | |
| **tBN-DNPTrz** | 529 | 0.0026 | |
| **NBO-Ac** | 467 | 0.17 | 73 |
| **NBO-PXZ** | 500 | 2.84 | |
| **tBCQBN** | 484 | 0.054 | 74 |
| **BN-Q-Ph** | 474 | 1.50 | |
| **BBCz-DB** | 474 | 0.35 | 75 |
| **4tCz2B** | 508 | 7.80 | |
| **m-PAz-BNCz** | 528 | 0.142 | 76 |
| **m-SFAz-BNCz** | 532 | 0.053 | |
| **m-DPAcPAz-BNCz** | 524 | 0.069 | |
| **m-TPAz-BNCz** | 532 | 0.085 | |
| **model-C-NPh2** | 458 | 8.0 | 77 |
| **model-A-NPh2** | 446 | 11.5 | |
| **model-B-NPh2** | 471 | 3.80 | |
| **DBN-amICz** | 490 | 0.42 | 78 |
| **DBN-bmlCz** | 490 | 0.57 | |
| **BN-PhAzCz** | 527 | 0.64 | 79 |
| **BN-tCzAzPh** | 525 | 0.24 | |
| **tBO-4B** | 452 | 42.3 | 80 |

## 3. **X-ray Crystal Structure Figures**

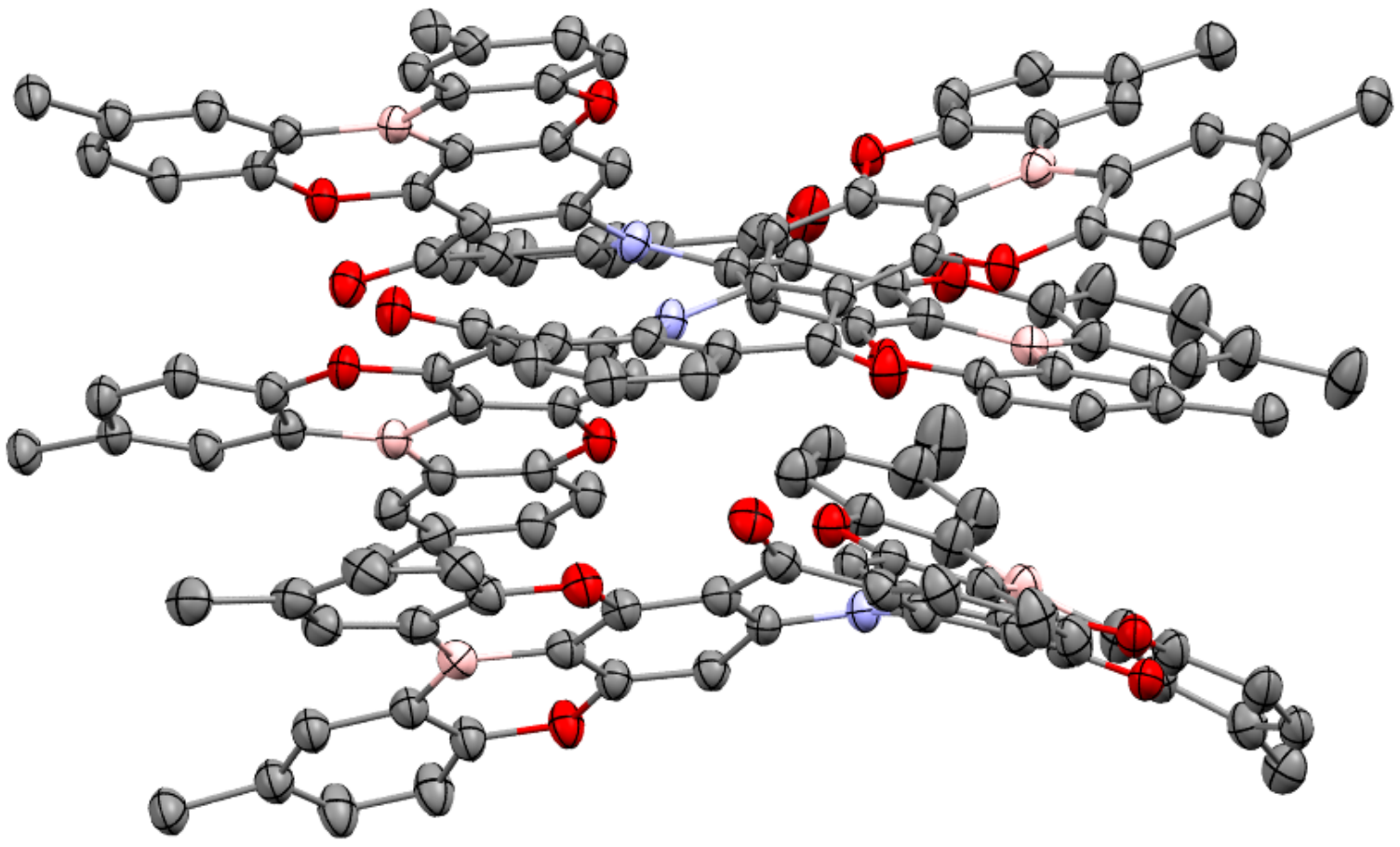

Figure **S36**. Structure of **DDOBDiKTa** showing 3 independent molecules in the asymmetric unit with ellipsoids drawn at 30% probability. Terminal methyl groups of *tert*-butyl moieties, solvate, hydrogen atoms, and minor parts of disorder omitted for clarity.

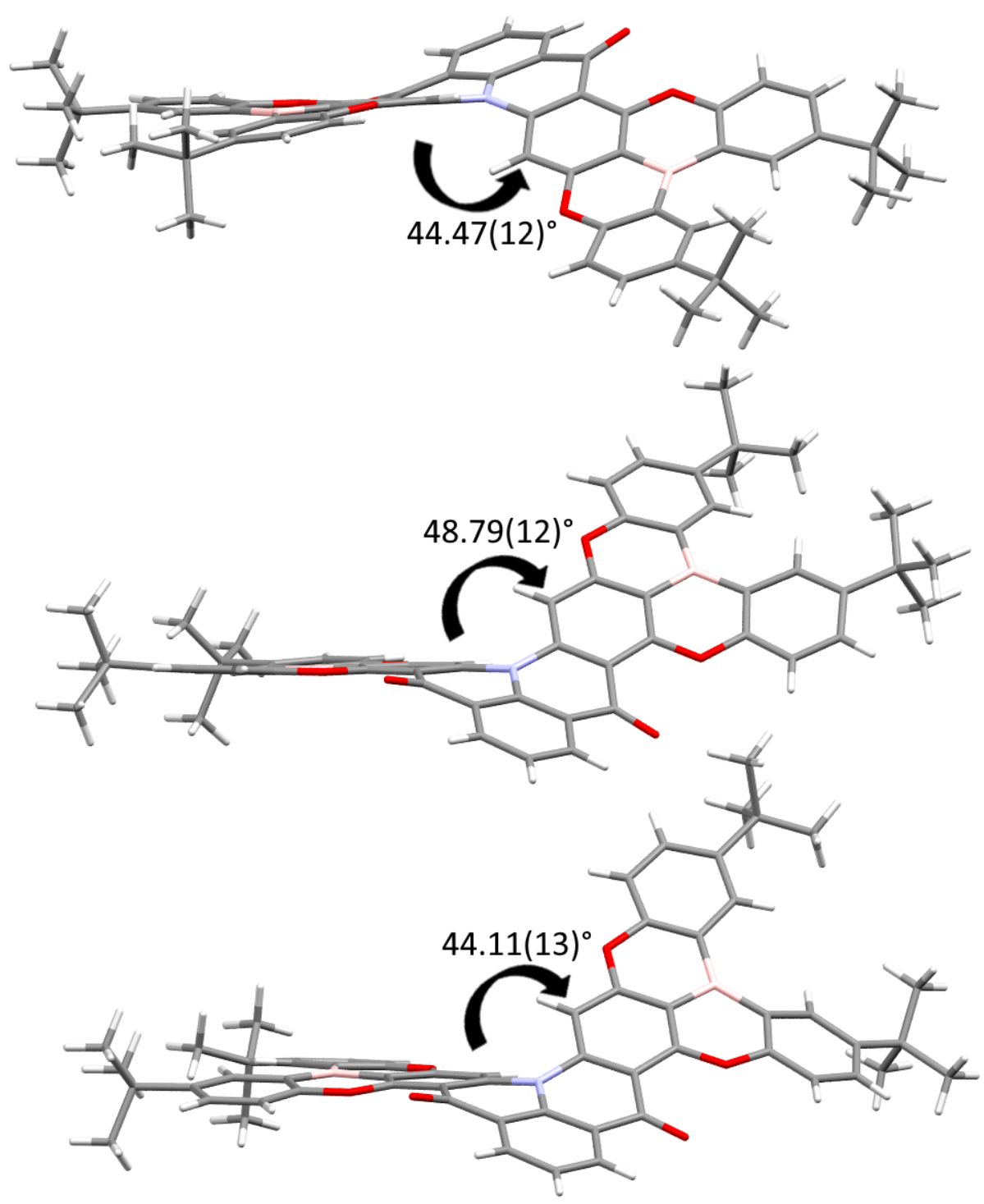


Figure **S37**. The dihedral angles across each independent molecule in the structure of **DDOB-DiKTa**. Angles are between the planes [C109, C111, C113] to [C95, C97, C99] (top), [C177, C179, C181] to [C163, C165, C167] (middle), and [C42, C44, C46] to [C28, C30, C32] (bottom). Molecules spaced apart and minor parts of disorder omitted for clarity.

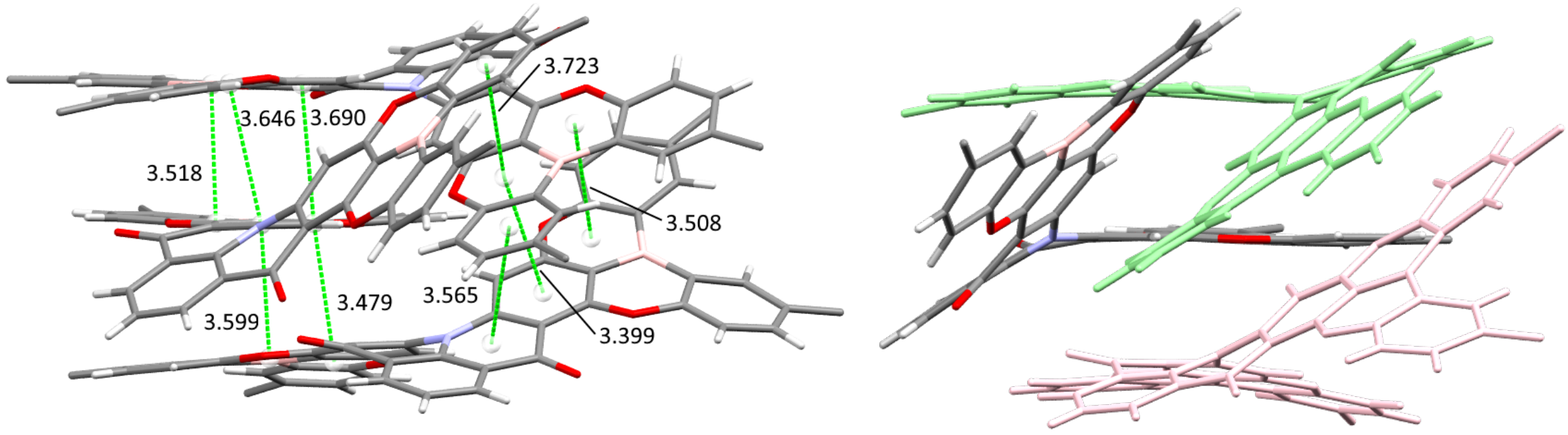


Figure **S38**. ABB stacked unit of **DDOBDiKTa** (left) with π···π stacking distances (centroid···centroid, Å) indicated (green dashed-lines) and rotated view (right) with independent A (green) and B2 (pink) in single colours. Terminal methyl groups of *tert*-butyl moieties, solvates, and minor parts of disorder omitted for clarity.

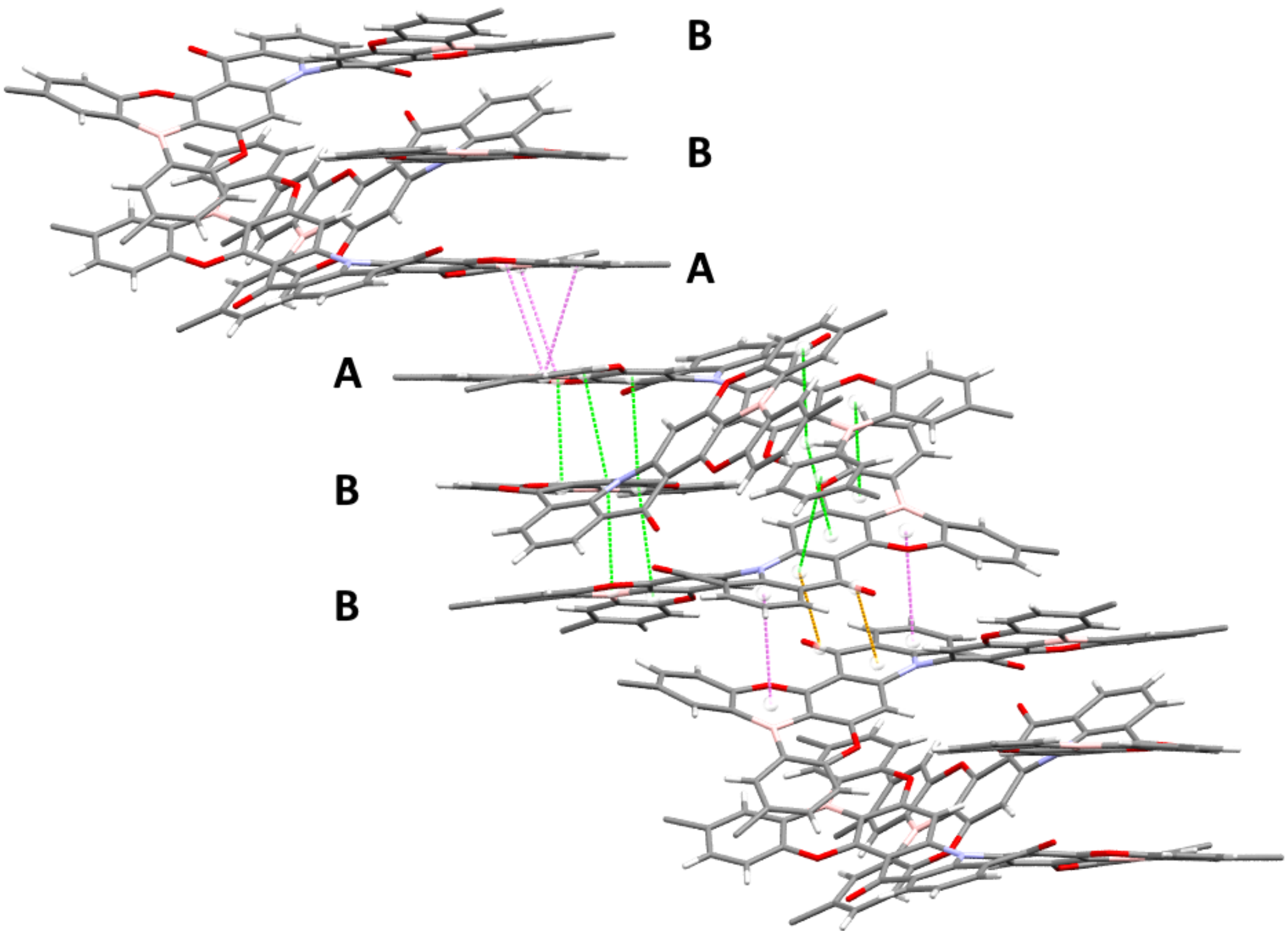


Figure **S39**. Repeating BBA·ABB stacks along [01-1] (top left to bottom right) with π···π (violet dashed-lines) and C=O···π (orange dashed-lines) between units. Terminal methyl groups of *tert*-butyl moieties, solvates, and minor parts of disorder omitted for clarity.

CCDC 2497380 contains the supplementary crystallographic data for this paper. These data can be obtained free of charge from The Cambridge Crystallographic Data Centre via www.ccdc.cam.ac.uk/structures.

## References


[1] M. J. Frisch, G. W. Trucks, H. B. Schlegel, G. E. Scuseria, M. A. Robb, J. R. Cheeseman, G. Scalmani, V. Barone, G. A. Petersson, H. Nakatsuji, X. Li, M. Caricato, A. V. Marenich, J. Bloino, B. G. Janesko, R. Gomperts, B. Mennucci, H. P. Hratchian, J. V. Ortiz, A. F. Izmaylov, J. L. Sonnenberg, Williams, F. Ding, F. Lipparini, F. Egidi, J. Goings, B. Peng, A. Petrone, T. Henderson, D. Ranasinghe, V. G. Zakrzewski, J. Gao, N. Rega, G. Zheng, W. Liang, M. Hada, M. Ehara, K. Toyota, R. Fukuda, J. Hasegawa, M. Ishida, T. Nakajima, Y. Honda, O. Kitao, H. Nakai, T. Vreven, K. Throssell, J. A. Montgomery Jr., J. E. Peralta, F. Ogliaro, M. J. Bearpark, J. J. Heyd, E. N. Brothers, K. N. Kudin, V. N. Staroverov, T. A. Keith, R. Kobayashi, J. Normand, K. Raghavachari, A. P. Rendell, J. C. Burant, S. S. Iyengar, J. Tomasi, M. Cossi, J. M. Millam, M. Klene, C. Adamo, R. Cammi, J. W. Ochterski, R. L. Martin, K. Morokuma, O. Farkas, J. B. Foresman, D. J. Fox, *Gaussian 16 Revision C.01*, Wallingford, CT, **2016**.

[2] C. Adamo, V. Barone, *J. Chem. Phys.* **1999**, *110*, 6158.

[3] G. A. Petersson, M. A. Al-Laham, *J. Chem. Phys.* **1991**, *94*, 6081.

[4] S. Grimme, J. Antony, S. Ehrlich, H. Krieg, *J. Chem. Phys.* **2010**, *132*, 154104.

[5] R. Dennington, T. A. Keith, J. M. Millam, *GaussView, Version 6*, Semichem Inc., Shawnee Mission, KS: **2016**.

[6] S. Hirata, M. Head-Gordon, *Chem. Phys. Lett.* **1999**, *314*, 291.

[7] X. Gao, S. Bai, D. Fazzi, T. Niehaus, M. Barbatti, W. Thiel, *J. Chem. Theory Comput.* **2017**, *13*, 515.

[8] W. Humphrey, A. Dalke, K. Schulten, *J. Mol. Graph.* **1996**, *14*, 33.

[9] *TURBOMOLE V7.5* ***2020****, a development of University of Karlsruhe and Forschungszentrum Karlsruhe GmbH,* ***1989-2007****,*, 7.5; TURBOMOLE GmbH, since **2007**; available from https://www.turbomole.org.: **2020**.

[10] S. G. Balasubramani, G. P. Chen, S. Coriani, M. Diedenhofen, M. S. Frank, Y. J. Franzke, F. Furche, R. Grotjahn, M. E. Harding, C. Hattig, A. Hellweg, B. Helmich-Paris, C. Holzer, U. Huniar, M. Kaupp, A. Marefat Khah, S. Karbalaei Khani, T. Muller, F. Mack, B. D. Nguyen, S. M. Parker, E. Perlt, D. Rappoport, K. Reiter, S. Roy, M. Ruckert, G. Schmitz, M. Sierka, E. Tapavicza, D. P. Tew, C. van Wullen, V. K. Voora, F. Weigend, A. Wodynski, J. M. Yu, *J. Chem. Phys.* **2020**, *152*, 184107.

[11] C. Hättig, F. Weigend, *J. Chem. Phys.* **2000**, *113*, 5154.

[12] A. Hellweg, S. A. Grün, C. Hättig, *Phys. Chem. Chem. Phys.* **2008**, *10*, 4119.

[13] J. Schirmer, *Phys. Rev. A,* **1982**, *26*, 2395.

[14] A. B. Trofimov, J. Schirmer, *J. Phys. B: At. Mol. Opt. Phys.* **1995**, *28*, 2299.

[15] T. H. Dunning, Jr., *J. Chem. Phys.* **1989**, *90*, 1007.

[16] C. Møller, M. S. Plesset, *Phys. Rev.* **1934**, *46*, 618.

[17] A. Tajti, L. Tulipan, P. G. Szalay, *J. Chem. Theory Comput.* **2020**, *16*, 468.

[18] C. Hättig, A. Köhn, *J. Chem. Phys.* **2002**, *117*, 6939.

[19] C. Hättig, K. Hald, *Phys. Chem. Chem. Phys.* **2002**, *4*, 2111.

[20] C. Hättig, A. Köhn, K. Hald, *J. Chem. Phys.* **2002**, *116*, 5401.

[21] O. S. Lee, E. Zysman-Colman, *Digichem (version 6) InSilico Computing*. St Andrews, Scotland: **2024**.

[22] O. Lee, M. Gather, E. Zysman-Colman, *Digit. Discov.* **2024**, *3*, 1695.

[23] N. M. O'Boyle, A. L. Tenderholt, K. M. Langner, *J. Comput. Chem.* **2008**, *29*, 839.

[24] J. E. Stone. An efficient library for parallel ray tracing and animation. University of Missouri - Rolla, **1998**.

[25] J. D. Hunter, *Comput. Sci. Eng.* **2007**, *9*, 90.

[26] N. M. O'Boyle, M. Banck, C. A. James, C. Morley, T. Vandermeersch, G. R. Hutchison, *J. Cheminform.* **2011**, *3*, 1.

[27] N. M. O'Boyle, G. R. Hutchison, *Chem. Cent. J.* **2008**, *2*, 24.

[28] G. A. Crosby, J. N. Demas, *J. Phys. Chem.* **1971**, *75*, 991.

[29] W. H. Melhuish, *J. Phys. Chem.* **1961**, *65*, 229.

[30] V. V. Pavlishchuk, A. W. Addison, *Inorg. Chim. Acta.* **2000**, *298*, 97.

[31] *CrysAlisPro* v1.171. 44.121a Rigaku Oxford Diffraction, Rigaku Corporation, *Tokyo*, Japan, **2025**.

[32] G. M. Sheldrick, SHELXT – Integrated space-group and crystal structure determination. *Acta Crystallogr., Sect. A: Found. Adv.* **2015**, *71*, 3-8.

[33] G. M. Sheldrick, Crystal structure refinement with SHELXL. *Acta Crystallogr., Sect. C: Struct. Chem.* **2015**, *71*, 3-8.

[34] A. L. Spek, PLATON SQUEEZE: a tool for the calculation of the disordered solvent contribution to the calculated structure factors. *Acta Crystallogr. Sect C: Struct. Chem.* **2015**, *71*, 9-18.

[35] A. L. Spek, Structure validation in chemical crystallography. *Acta Crystallogr. Sect D: Biol. Crystallogr.* **2009**, *65*, 148-155.

[36] O. V. Dolomanov, L. J. Bourhis, R. J. Gildea, J. A. K. Howard, H. Puschmann, OLEX2: a complete structure solution, refinement and analysis program. *J. Appl. Crystallogr.* **2009**, *42*, 339-341.

[37] I. Sokolovs, N. Mohebbati, R. Francke, E. Suna, *Angew. Chem. Int. Ed.* **2021**, *60*, 15832–15837.

[38] Y. Tsuchiya, S. Diesing, F. Bencheikh, Y. Wada, P. L. dos Santos, H. Kaji, E. Zysman-Colman, I. D. W. Samuel, C. Adachi, *J. Phys. Chem. A* **2021**, *125*, 8074–8089.

[39] T. Hatakeyama, K. Shiren, K. Nakajima, S. Nomura, S. Nakatsuka, K. Kinoshita, J. Ni, Y. Ono, T. Ikuta, *Adv. Mater.* **2016**, *28*, 2777–2781.

[40] Y. Kondo, K. Yoshiura, S. Kitera, H. Nishi, S. Oda, H. Gotoh, Y. Sasada, M. Yanai, T. Hatakeyama, *Nat. Photonics* **2019**, *13*, 678–682.

[41] F. Liu, Z. Cheng, L. Wan, Z. Feng, H. Liu, H. Jin, L. Gao, P. Lu, W. Yang, *Small* **2022**, *18*, 2106462.

[42] Y. Zhang, D. Zhang, J. Wei, Z. Liu, Y. Lu, L. Duan, *Angew. Chem. Int. Ed.* **2019**, *58*, 16912–16917.

[43] J. Han, Z. Huang, X. Lv, J. Miao, Y. Qiu, X. Cao, C. Yang, *Adv. Opt. Mater.* **2022**, *10*, 2102092.

[44] H. Tanaka, S. Oda, G. Ricci, H. Gotoh, K. Tabata, R. Kawasumi, D. Beljonne, Y. Olivier, T. Hatakeyama, *Angew. Chem. Int. Ed.* **2021**, *60*, 17910–17914.

[45] S. Oda, W. Kumano, T. Hama, R. Kawasumi, K. Yoshiura, T. Hatakeyama, *Angew. Chem. Int. Ed.* **2021**, *60*, 2882–2886.

[46] Y. Xu, Q. Wang, X. Cai, C. Li, Y. Wang, *Adv. Mater.* **2021**, *33*, 2100652.

[47] M. Yang, I. S. Park, T. Yasuda, *J. Am. Chem. Soc.* **2020**, *142*, 19468–19472.

[48] M. Yang, S. Shikita, H. Min, I. S. Park, H. Shibata, N. Amanokura, T. Yasuda, *Angew. Chem. Int. Ed.* **2021**, *60*, 23142–23147.

[49] I. S. Park, M. Yang, H. Shibata, N. Amanokura, T. Yasuda, *Adv. Mater.* **2022**, *34*, 2107951.

[50] Y. Wang, Y. Duan, R. Guo, S. Ye, K. Di, W. Zhang, S. Zhuang, L. Wang, *Org. Electron.* **2021**, *97*, 106275.

[51] J. Park, J. Lim, J. H. Lee, B. Jang, J. H. Han, S. S. Yoon, J. Y. Lee, *ACS Appl. Mater. Interfaces* **2021**, *13*, 45798–45805.

[52] S. Oda, B. Kawakami, R. Kawasumi, R. Okita, T. Hatakeyama, *Org. Lett.* **2019**, *21*, 9311–9314.

[53] Y. Qiu, H. Xia, J. Miao, Z. Huang, N. Li, X. Cao, J. Han, C. Zhou, C. Zhong, C. Yang, *ACS Appl. Mater. Interfaces* **2021**, *13*, 59035–59042.

[54] Y. T. Lee, C. Y. Chan, M. Tanaka, M. Mamada, U. Balijapalli, Y. Tsuchiya, H. Nakanotani, T. Hatakeyama, C. Adachi, *Adv. Electron. Mater.* **2021**, *7*, 2001090.

[55] K. R. Naveen, H. Lee, L. H. Seung, Y. H. Jung, C. P. Keshavananda Prabhu, S. Muruganantham, J. H. Kwon, *Chem. Eng. J.* **2023**, *451*, 138498.

[56] Y. C. Cheng, X. Tang, K. Wang, X. Xiong, X. C. Fan, S. Luo, R. Walia, Y. Xie, T. Zhang, D. Zhang, J. Yu, X. K. Chen, C. Adachi, X. H. Zhang, *Nat. Commun.* **2024**, *15*, 731.

[57] R. W. Weerasinghe, S. Madayanad Suresh, D. Hall, T. Matulaitis, A. M. Z. Slawin, S. Warriner, Y. T. Lee, C. Y. Chan, Y. Tsuchiya, E. Zysman-Colman, C. Adachi, *Adv. Mater.* **2024**, *36*, 2402289.

[58] S. Madayanad Suresh, L. Zhang, D. Hall, C. Si, G. Ricci, T. Matulaitis, A. M. Z. Slawin, S. Warriner, Y. Olivier, I. D. W. Samuel, E. Zysman-Colman, *Angew. Chem. Int. Ed.* **2023**, *62*, e202215522.

[59] X. Cai, Y. Pu, C. Li, Z. Wang, Y. Wang, *Angew. Chem. Int. Ed.* **2023**, *62*, e202304104.

[60] K. Zhang, X. Wang, Y. Chang, Y. Wu, S. Wang, L. Wang, *Angew. Chem. Int. Ed.* **2023**, *62*, e202313084.

[61] K. R. Naveen, J. H. Oh, H. S. Lee, J. H. Kwon, *Angew. Chem. Int. Ed.* **2023**, *62*, e202306768.

[62] S. Uemura, S. Oda, M. Hayakawa, R. Kawasumi, N. Ikeda, Y. T. Lee, C. Y. Chan, Y. Tsuchiya, C. Adachi, T. Hatakeyama, *J. Am. Chem. Soc.* **2023**, *145*, 1505–1511.

[63] M. Mamada, A. Aoyama, R. Uchida, J. Ochi, S. Oda, Y. Kondo, M. Kondo, T. Hatakeyama, *Adv. Mater.* **2024**, *36*, 2402905.

[64] G. Chen, J. Wang, W. C. Chen, Y. Gong, N. Zhuang, H. Liang, L. Xing, Y. Liu, S. Ji, H. L. Zhang, Z. Zhao, Y. Huo, B. Z. Tang, *Adv. Funct. Mater.* **2023**, *33*, 2211893.

[65] S. Wang, Y. Xu, J. Miao, T. Hua, X. Cao, N. Li, Z. Huang, C. Yang, *Chem. Eng. J.* **2023**, *471*, 144664.

[66] G. Meng, J. Zhou, T. Huang, H. Dai, X. Li, X. Jia, L. Wang, D. Zhang, L. Duan, *Angew. Chem. Int. Ed.* **2023**, *62*, e202309923.

[67] S. Wu, L. Zhang, J. Wang, A. Kumar Gupta, I. D. W. Samuel, E. Zysman-Colman, *Angew. Chem. Int. Ed.* **2023**, *62*, e202305182..

[68] Z. Xue, Z. Xiao, Y. Zou, Z. Chen, J. Liu, Z. Huang, C. Yang, *Chem. Sci.* **2025**, *16*, 3655–3661.

[69] Z. Xue, Y. Hu, S. Xiao, J. Liu, J. Miao, C. Yang, *Angew. Chem. Int. Ed.* **2025**, *64*, e202500108.

[70] Z. Xiao, Y. Zou, Z. Chen, J. Miao, Y. Qiu, Z. Huang, X. Cao, X. Peng, C. Yang, *Adv. Mater.* **2025**, *37*, 2419601.

[71] H. Min, T. Yasuda, *Adv. Opt. Mater.* **2025**, *13*, 2500384.

[72] H. U. Kim, T. H. Ha, P. Palanisamy, S. W. Kang, J. G. Shin, J. Y. Yoo, K. M. Park, O. P. Kumar, C. W. Lee, J. H. Kwon, *Chem. Eng. J.* **2025**, *507*, 160656.

[73] C. L. Hou, S. Guo, Y. F. Sun, J. X. Hu, H. J. Liao, X. Li, F. L. Lin, L. Meng, X. L. Chen, C. Z. Lu, *Chem. Eng. J.* **2025**, *508*, 160880.

[74] H. Xie, M. Li, Z. Li, Z. Chen, Z. Yang, K. Liu, S. J. Su, *J. Mater. Chem. C* **2025**, *13*, 7537–7543.

[75] L. Guo, W. Cui, L. Li, Y. Pu, K. Wang, P. Zheng, Y. Wang, *Adv. Mater.* **2025**, *37*, 2500269.

[76] T. Huang, Y. Xu, Y. Qu, X. Lu, K. Ye, X. Zhuang, Y. Wang, *Adv. Mater*. **2025**, *37*, 2503383.

[77] J. Ochi, Y. Yamasaki, S. Oda, M. Kondo, A. Ikeno, Y. Kondo, T. Hatakeyama, *Adv. Opt. Mater.* **2025**, *13*, 2402939.

[78] L. Li, T. Huang, Y. Xu, Y. Qu, W. Cui, L. Xu, C. Li, Y. Wang, *Angew. Chem. Int. Ed.* **2025**, *64*, e202504002.

[79] Y. Qu, Y. Xu, T. Huang, X. Song, K. Ye, Y. Wang, *Angew. Chem. Int. Ed.* **2025**, *64*, e202506201.

[80] Y. C. Cheng, X. Tang, R. Walia, T. Y. Zhang, X. C. Fan, J. Yu, K. Wang, C. Adachi, X. K. Chen, X. H. Zhang, *Adv. Mater.* **2025**, *37*, 2500010.
